# Limiting future warming reduces drought exposure for terrestrial vertebrates

**DOI:** 10.1038/s41467-026-73229-3

**Published:** 2026-05-14

**Authors:** Yuchuan He, Jian Sun, Yanqiang Wei, Yanxu Liu, Michael E. Meadows, Josep Peñuelas

**Affiliations:** 1 State Key Laboratory of Tibetan Plateau Earth System, Environment and Resources (TPESER), Institute of Tibetan Plateau Research, Chinese Academy of Sciences, Beijing, China; 2 University of Chinese Academy of Sciences, Beijing, China; 3 State Key Laboratory of Cryospheric Science and Frozen Soil Engineering, Key Laboratory of Remote Sensing of Gansu Province, Northwest Institute of Eco-Environment and Resources, Chinese Academy of Sciences, Lanzhou, China; 4 State Key Laboratory of Earth Surface Processes and Hazards Risk Governance, Faculty of Geographical Science, Beijing Normal University, Beijing, China; 5 School of Geography and Oceanographic Sciences, Nanjing University, Nanjing, China; 6 Department of Environmental & Geographical Science, University of Cape Town, Rondebosch, South Africa; 7 CREAF, Cerdanyola del Valles, Barcelona, Spain; 8 CSIC, Global Ecology Unit CREAF-CSIC-UAB, Barcelona, Spain

**Keywords:** Climate-change ecology, Biodiversity

## Abstract

Ambitious conservation efforts are needed to curb biodiversity loss as drought severity intensifies globally. Here, we assess the exposure of resident terrestrial vertebrates within global biodiversity hotspots to drought severity surpassing the extremes experienced during their pre-industrial history. We show that 22.5% of threatened terrestrial vertebrates (especially reptiles and amphibians) have recently experienced drought severity exceeding their historical extremes across at least half of their current geographic range. Under an intermediate greenhouse gas emission scenario (Shared Socioeconomic Pathway 2–4.5), this proportion is projected to reach 36.5% by the latter half of the 21st century, with mid-latitude dryland biodiversity hotspots facing the most severe drought exposure. Importantly, a low-warming future (Shared Socioeconomic Pathway 1–2.6) will reduce exposure estimates of species by 8.5% compared to Shared Socioeconomic Pathway 2–4.5, highlighting the urgency of ambitious climate mitigation. However, as future drought exposure is projected to increase across most biodiversity hotspots, and many exposed regions face inadequate protection and substantial social burdens, expanding adaptive conservation without compromising local well-being is essential. Our findings offer spatial guidance for prioritizing conservation and adaptive strategies in biodiversity hotspots, contributing to global biodiversity targets.

## Abstract (web summary)

Drought extremes have considerable impacts on ecosystems. Here, the results show that limiting future warming can reduce the exposure of terrestrial vertebrates to drought severity exceeding their historical extremes.

## Introduction

**Fig. 1:**
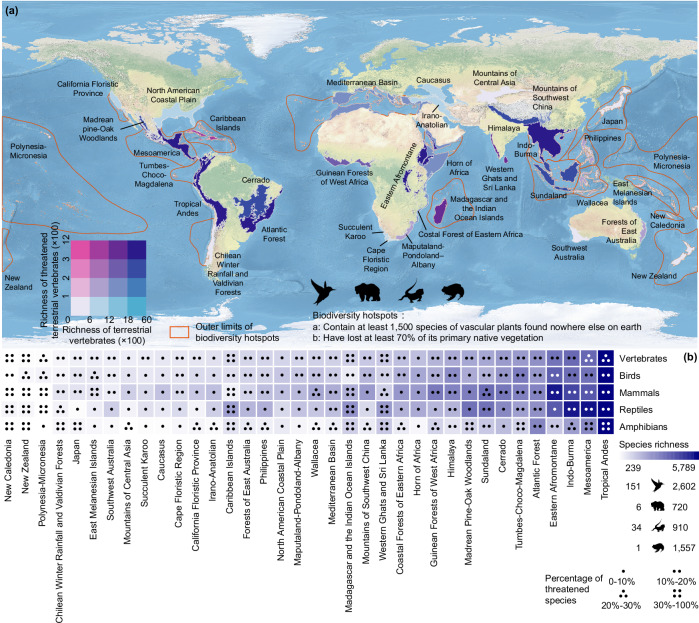
Richness of terrestrial vertebrates in biodiversity hotspots (BHs). **a** Map of 36 BHs, with the bivariate map showing the species richness of all and threatened terrestrial vertebrates, with the orange lines representing the outer limits of BHs. The background image is from Natural Earth (https://www.naturalearthdata.com/). **b** Species richness of all terrestrial vertebrates, birds, mammals, reptiles, and amphibians in each BH, with dots representing the percentage of threatened species in that category. One, two, three, and four dots denote 0–10%, 10–20%, 20–30%, and 30–100%, respectively. Richness of terrestrial vertebrates was extracted from distribution ranges of year-round resident species provided by the International Union for Conservation of Nature (IUCN) and BirdLife International. Threatened species are those classified as vulnerable, endangered, or critically endangered by the IUCN Red List of Threatened Species. Source data are provided as a Source Data file.

Prioritizing and safeguarding global Biodiversity Hotspots (BHs, the most species-rich yet highly threatened terrestrial regions^1–3^, Fig. 1) is central to the Kunming-Montreal Global Biodiversity Framework (GBF)’s ambitions of halting and reversing biodiversity loss^4^. However, the conservation priorities for BHs under accelerating climate warming remain unclear^3,5,6^. Anthropogenic climate change is exacerbating the frequency, duration, and intensity of drought events globally at an unprecedented rate and is expected to further increase in the future^7–13^. Recurring droughts degrade habitat quality, directly affecting species survival strategies, increasing physiological stresses such as heat stress and dehydration, causing reproduction failure and population decrease^14–19^. Recent extreme droughts have already resulted in habitat loss and population die-offs in localized areas within BHs^3,17,20,21^. Therefore, evaluating the impacts of intensifying drought on biodiversity in critical yet vulnerable BHs is fundamental to guiding pre-emptive conservation efforts^3,22^.

Although the threat of droughts to species and ecosystem viability is widely recognized^14–21^, their ecological consequences may be further amplified by reduced opportunities for species to adapt and survive due to anthropogenic habitat loss and fragmentation^23–25^. In fact, most BHs are located in densely populated and underdeveloped regions^2,3^, where increasing livelihood needs often compete with biodiversity conservation in terms of land use and financial resources^5,26,27^. Habitat deterioration caused by extensive urbanization^28^, agricultural and renewable energy expansion^23,29^, deforestation^5^, or transboundary fencing^30^ is undermining potential climate refuges for species and hindering their dispersal to track their climate envelopes^23,31^. Consequently, identifying the species and regions most affected by intensifying drought in BHs, and clarifying their conservation gaps and governance challenges, can inform decisions to develop or modify equitable and adaptive conservation measures^5,32^.

Previous studies integrating climate change simulations with species distribution modeling demonstrate that species, both globally and within BHs, are increasingly exposed to droughts and other climate extremes^14,33–39^. However, most existing assessments quantify these impacts using generalized climate anomalies or fixed historical thresholds^19,39^. While such approaches capture intensifying drought conditions, they often overlook the species-specific differences in drought vulnerability, which are critical determinants of survival^19,39^. Given the scarcity of physiological data for most species^39^, we approximate drought tolerance limits using the historical extremes of species realized climatic niches. We then assess the exposure of species’ geographic ranges to recent and future drought severity exceeding their historical extremes (see Supplementary Fig. 1 and Methods). Focusing on year-round resident threatened terrestrial vertebrates (as identified by the International Union for Conservation of Nature^40^) within BHs, this analysis identifies critical conservation gaps and governance challenges in regions facing drought exposure, providing spatially explicit guidance for conservation planning.

## Results

### Intensifying drought severity

**Fig. 2:**
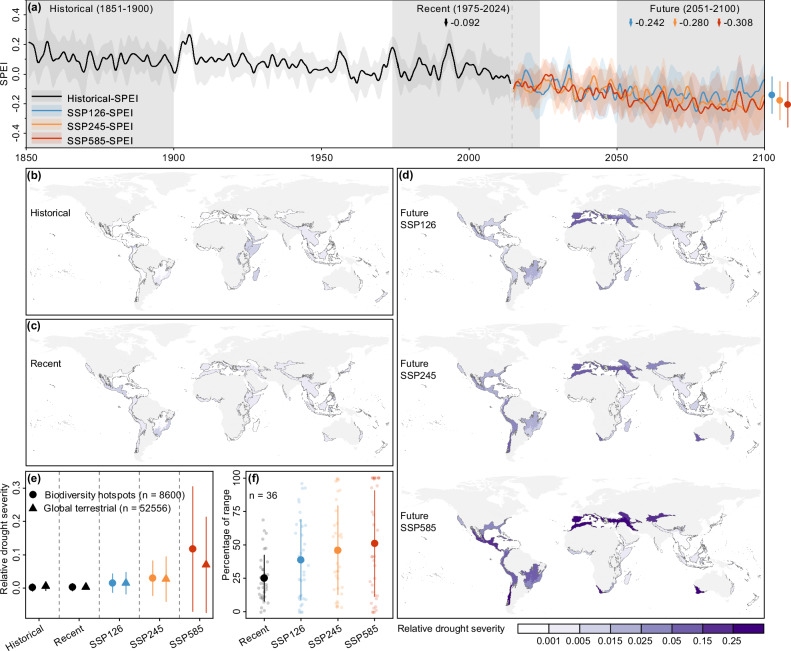
Quantitative synthesis of drought severity in biodiversity hotspots (BHs). **a** Temporal dynamics of the standardized precipitation evapotranspiration index (SPEI) in BHs. SPEI is used to calculate drought severity. SPEI trajectories for 1851 to 2100 are medians from five Global Climate Model estimates, with shading indicating standard deviation. Arrows in the corresponding periods represent variations of SPEI relative to the historical period. Error bars on the right illustrate the potential range of SPEI in the latter half of the 21st century under the three future scenarios. Black represents historical and recent periods, and blue, orange, and red represent future scenarios under Shared Socioeconomic Pathway 1–2.6 (SSP126), SSP245, and SSP585, respectively. Spatial patterns of drought severity in BHs under historical (**b** 1851 to 1900), recent (**c** 1975 to 2024), and three future scenarios (**d** SSP126, SSP245, and SSP585 for 2051 to 2100). **e** Average drought severity in BHs and global terrestrial regions for historical, recent, and future periods. Data are presented as mean values +/– standard deviation, and the numbers of *n* represent the number of data entries in the corresponding region. **f** Average percentage of range where recent and future drought severity exceeds historical levels in BHs. Data are presented as mean values +/− standard deviation, and the number of *n* represents the number of biodiversity hotspots (*n* = 36). Source data are provided as a Source Data file.

Drought severity was defined as the cumulative sum of drought intensity exceeding the drought threshold for a given period^9–11^ (see Methods). To characterize drought dynamics in BHs following large-scale anthropogenic warming, we quantified and compared the drought severity for historical (pre-industrial, 1851 to 1900), recent (1975 to 2024), and future (2051 to 2100) periods. To cover the potential range of future anthropogenic climate change, we estimated projections under three warming scenarios: Shared Socioeconomic Pathway 1–2.6 (SSP126), a low greenhouse gas emission scenario; SSP245, an intermediate-emission scenario; and SSP585, a high-emission scenario (see Methods for details). The results demonstrate that climate change is persistently intensifying the drought severity in BHs and globally (Fig. 2 and Supplementary Figs. 2–4). Relative to the historical baseline, average drought severity in BHs has recently increased by 48.9% and is projected to exceed the global terrestrial average in the future (Fig. 2e). Under SSP245, nearly half (46.0%) of the areas in BHs will experience drought severity above historical levels, increasing to 51.0% under SSP585 (Fig. 2f and Supplementary Fig. 5). Even under the stringent climate mitigation scenario (SSP126), this proportion remains substantial at 38.8%. BHs primarily located in mid-latitude drylands such as deserts, grasslands, shrublands, and savannas are projected to experience more substantial increases in drought severity (Fig. 2d, and Supplementary Figs. 6–7).

### Exposure to drought severity exceeding historical extremes

**Fig. 3:**
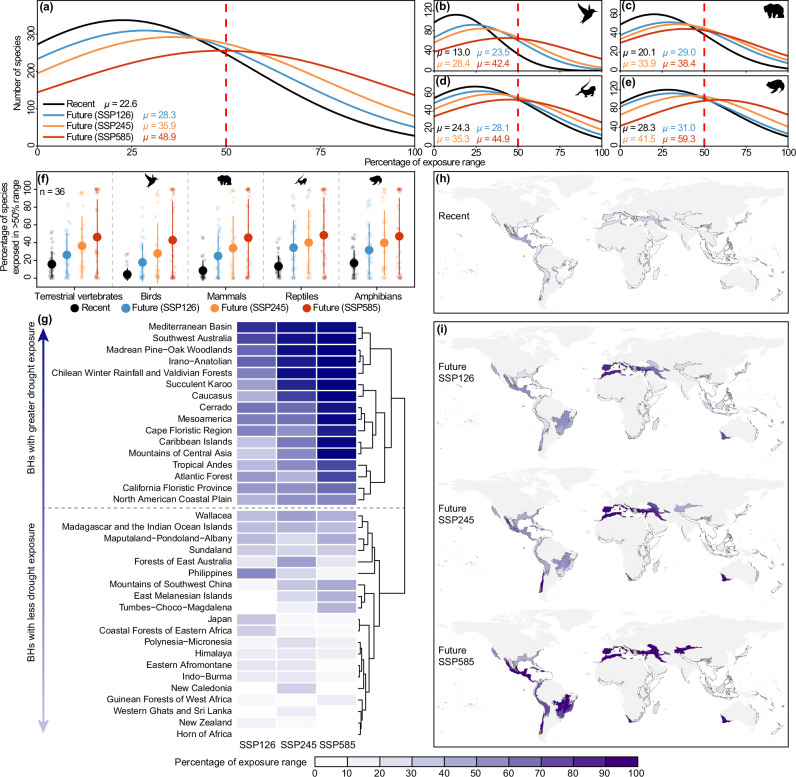
Species geographic range exposure to drought severity exceeding historical extremes in biodiversity hotspots (BHs). Distribution of percentage of exposure range to drought severity exceeding historical extremes for threatened terrestrial vertebrates (**a**), birds (**b**), mammals (**c**), reptiles (**d**), and amphibians (**e**) in BHs under recent and three future scenarios, with *μ* denoting the overall mean of the distribution. Black represents recent period, and blue, orange, and red represent future scenarios under Shared Socioeconomic Pathway 1–2.6 (SSP126), SSP245, and SSP585, respectively. **f** The percentage of species exposed to drought severity exceeding historical extremes in ≥ 50% of their geographic range for threatened terrestrial vertebrates, birds, mammals, reptiles, and amphibians in BHs under recent and three future scenarios. Data are presented as mean values +/– standard deviation, and the number of *n* represents the number of biodiversity hotspots (*n* = 36). **g** Average percentage of exposure range to drought severity exceeding historical extremes for threatened terrestrial vertebrates in each BH under three future scenarios, clustering all SSPs into greater and less drought exposure. Spatial patterns of average percentage of exposure range to drought severity exceeding historical extremes for threatened terrestrial vertebrates in BHs under recent (**h**) and three future scenarios (**i**). Source data are provided as a Source Data file.

We assessed the geographic range exposure of year-round resident terrestrial vertebrates in BHs to recent and future drought severity exceeding their historical extremes (see Supplementary Fig. 1 and Methods). The estimates indicate that an average of 22.6% of geographic range in BHs for threatened terrestrial vertebrates has been exposed to drought severity exceeding their historical extremes in recent decades (Fig. 3a). Under SSP245, by the latter half of the 21st century, the average percentage of exposure range is projected to increase to 35.9%, as high as 48.9% under SSP585. Although the average estimate is lower for SSP126 (28.3%), it remains above recent levels. Across taxa, the average percentage of exposure range for threatened amphibians (recent: 28.3%; SSP245: 41.5%; SSP585: 59.3%; SSP126: 31.0%, Fig. 3e) and reptiles (recent: 24.3%; SSP245: 35.3%; SSP585: 44.9%; SSP126: 28.1%, Fig. 3d) are higher compared to threatened mammals (recent: 20.1%; SSP245: 33.9%; SSP585: 38.4%; SSP126: 29.0%, Fig. 3c) and birds (recent: 13.0%; SSP245: 28.4%; SSP585: 42.4%; SSP126: 23.5%, Fig. 3b). Notably, the average exposure estimates for threatened terrestrial vertebrates are higher than other terrestrial vertebrates (Supplementary Fig. 8). Overall, 22.5% of threatened terrestrial vertebrates in BHs have recently experienced drought severity exceeding their historical extremes across at least half ( ≥ 50%) of their current geographic range, and this percentage of species will respectively rise to 36.5% (SSP245), 50.0% (SSP585), and 28.0% (SSP126) in the future (Fig. 3f).

In the future, BHs facing greater drought exposure are mainly in mid-latitude drylands (especially under SSP245), followed by some tropical American BHs (Fig. 3g, i). Specifically, under SSP245, the Mediterranean Basin, Southwest Australia, Madrean Pine-Oak Woodlands, Irano-Anatolian BH, Chilean Winter Rainfall and Valdivian Forests, Succulent Karoo, and Caucasus are projected to experience considerable exposure of species’ geographic ranges to drought severity exceeding their historical extremes. Drought exposure in the Cerrado, Mesoamerica, Cape Floristic Region, Mountains of Central Asia, Caribbean Islands, Tropical Ande, and Atlantic Forest will surge under SSP585. Spatial patterns of exposure vary with threatened terrestrial vertebrate classes (Supplementary Figs. 9–12). On average, threatened reptiles and amphibians face greater drought exposure than birds and mammals in most BHs, especially in the Mountains of Central Asia, California Floristic Province, Cape Floristic Region, Caucasus, Madagascar and the Indian Ocean Islands, and Forests of East Australia. Nonetheless, threatened mammals face greater exposure than other taxa in the North American Coastal Plain and Irano-Anatolian BHs.

### Benefits of limiting future warming

The drought exposure of species in BHs is sensitive to future anthropogenic warming trajectories, and limiting warming will reduce drought exposure for threatened terrestrial vertebrates (Fig. 3). Generally, limiting mean global warming to below 1.8 °C above pre-industrial levels by the end of the 21st century (SSP126) can effectively reduce the percentage of species that face drought severity exceeding historical extremes in ≥ 50% of their geographic range respectively by 8.5% from 36.5% under SSP245 (2.7 °C warming) and 22.0% from 50.0% under SSP585 (4.4 °C warming) (Fig. 3f). In terms of reduced average exposure ranges, limiting future warming provides greater benefits for threatened amphibians and reptiles than for threatened birds and mammals (Fig. 3b–e). Drought exposure in most American BHs (such as the Chilean Winter Rainfall and Valdivian Forests, Madrean Pine-Oak Woodlands, Caribbean Islands, Tropical Andes), as well as in the Succulent Karoo, Caucasus, and Mountains of Central Asia, will be substantially mitigated under SSP126 (Fig. 3g, i). These findings provide quantitative evidence that ambitious climate policies can mitigate the impacts of intensifying drought on biodiversity. However, even under an optimistic scenario (SSP126), future drought exposure in several BHs (such as the Southwest Australia and Mediterranean Basin, Fig. 3g) cannot be substantially mitigated, and most BHs are projected to experience greater exposure than recent levels (Fig. 3a–f). Moreover, the expansion of bioenergy cropland^41^ and renewable energy^29^ promoted by climate mitigation policies may accelerate habitat losses in BHs, thereby risking the offset of biodiversity gains from slowing warming.

### Conservation gaps and challenges

**Fig. 4:**
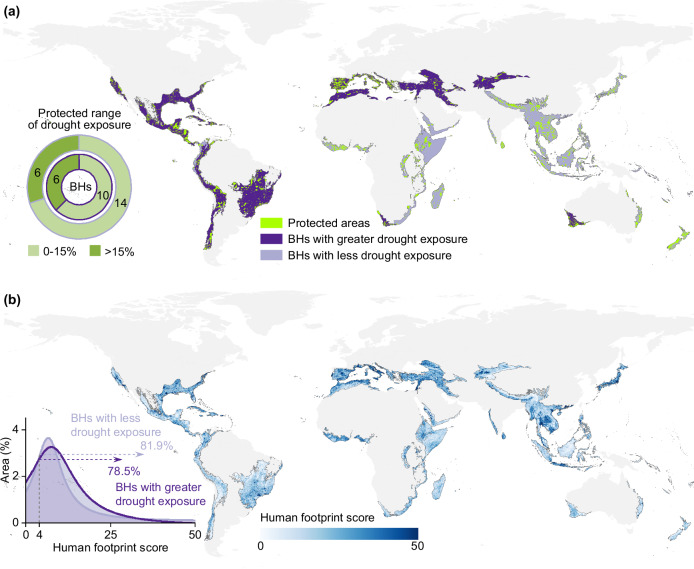
Protected areas and human pressure in biodiversity hotspots (BHs). **a** Distribution of protected areas in BHs. The left panel shows the current protection status of regions that will face future drought exposure: light green, 0–15% range protected; green, > 15% range protected. The percentage of protected area coverage is the average across the three SSPs. The inner circle shows the number of BHs with greater drought exposure, and the outer circle shows the number of BHs with less drought exposure. **b** Distribution of human footprints across BHs in 2020. The left graph shows the frequency distribution of human footprints in BHs with different future drought exposure levels: light purple, BHs with less drought exposure; purple, BHs with greater drought exposure. It also shows the percentage of area under intense human pressure (i.e., human footprint score > 4). Source data are provided as a Source Data file.

Since climate mitigation strategies cannot fully reverse the future increases in drought exposure for species, spatially tailoring conservation efforts is crucial for facilitating species adaptation to drought exposure^42–44^. By overlaying protected area networks, we found that only ~11% (recent: 12.1%; SSP245: 10.8%; SSP585: 11.1%; SSP126: 11.6%) of areas facing drought exposure in BHs are currently protected (Fig. 4a and Supplementary Fig. 13). Most individual BHs projected to experience greater drought exposure have protected-area coverage below 15%, well below the global target of protecting at least 30% of the key ecological areas by 2030 (30 × 30 target)^4^, particularly for the Caucasus, Irano-Anatolian BH, Atlantic Forest, and Mountains of Central Asia. This inadequate protection is presumably due to intense human competition for land^45^. Indeed, BHs with greater drought exposure are situated mainly in transboundary areas of developing countries (Supplementary Fig. 14a, b, and Supplementary Table 1), where nearly 1 billion people (Supplementary Fig. 14c) impose intensive human pressure on 78.5% of the area (human footprint score > 4^28^, Fig. 4b). Furthermore, numerous studies demonstrate that anthropogenic habitat conversion in BHs will be further amplified in the future^24,25,38^. Habitat loss and fragmentation from expanding human activities are reducing connectivity across BHs, undermining the potential adaptive strategies of species to drought (such as range shifts)^23,24,31^. Therefore, while expanding protected networks does not insulate against drought exposure driven by climate warming, it can support species persistence by providing refuge and maintaining habitat integrity and connectivity^42,44^. However, protected areas have shown mixed outcomes in resisting habitat loss^46^, and the implementation of conservation actions often entails trade-offs with other social priorities, including food production and economic development^26,47^. Additionally, applying top-down approaches to create protected areas may trigger conflicts with local livelihoods^26,27,47^. Consequently, understanding and minimizing these trade-offs in conservation decisions will be key to coping with intensifying drought exposure without compromising social well-being in BHs.

## Discussion

Here, we assess the threat posed by intensifying drought to resident threatened terrestrial vertebrates in Biodiversity Hotspots (BHs). Drought severity exceeding historical extremes experienced by species is detrimental to their survival^14–19^. Using species-specific historical drought severity extremes estimated from realized climate niches, we found that 22.5% of threatened terrestrial vertebrates in BHs have recently been exposed to drought severity exceeding their historical extremes across at least half of their current geographic range, with this proportion projected to reach 36.5% under an intermediate greenhouse gas emission future (SSP245). Mid-latitude dryland BHs will face severe drought exposure, where coupled thermal extremes may amplify thermodynamic responses^35^, overwhelming species that are already at the physiological limits of heat and water stress^15,25^. Analyses across taxa highlight that threatened amphibians and reptiles face greater drought exposure than birds and mammals in BHs, potentially due to the smaller geographic ranges occupied by most threatened amphibians and reptiles (Supplementary Fig. 15). The impacts of drought exposure are compounded by divergent drought vulnerability and adaptability across taxa^17,39^. In contrast, most birds and mammals possess more flexible drought-adaptation strategies, such as utilizing climate refuges^48^, thermoregulating^15,35^, or altering dietary structure and breeding rhythms^49,50^. Aquatic-dependent amphibians exposed to intensifying drought are more susceptible to physiological dehydration, impaired reproduction, or mortality^14,51^. While reptiles dominate drylands due to their drought tolerance^25^, their ectothermic nature renders their physiology tightly coupled to environmental temperatures^52^, making them especially sensitive to thermal extremes^53^. Importantly, drought-induced shifts in species survival strategies and ranges may reshape community structures and functions^17,18,54^, reshuffling competition, predation, symbiosis, or other species interactions^17,36,55^. For example, decreased productivity due to drought may constrain food sources for herbivores^14,15,56^, intensify competition resulting in population declines^17^, triggering prey shortages for predators^17,36^, and ultimately simplifying food networks^15^. Such cascading effects may permeate the threats of drought exposure across trophic levels, undermining biodiversity and other ecosystem services in BHs^14–18,36^.

The projected drought exposure in BHs demands ambitious actions for mitigation and adaptation. Our assessments show that limiting future warming could mitigate drought exposure for species, underscoring the urgent need to reduce greenhouse gas emissions and enhance carbon sequestration^7,57^. Even so, under an optimistic warming scenario, future drought exposure in BHs remains greater than recent exposure levels, and most exposed areas currently lack protection. Thus, prioritizing the protection, restoration, and connectivity of exposed habitats (such as removing fences^30^) may benefit species in tracking suitable climate niches^31,42,44,57^. Given that the dispersal capacity of many native or habitat-limited species (such as reptiles, amphibians, and small mammals) may lag during rapid drought expansion^34,58^, complementary measures (including identifying and protecting microhabitats and refuges^6,48^, rewilding to restore community integrity after extreme droughts^59^, and establishing new water sources to reduce abrupt heat and water stress^14^) may be critical for enhancing species resilience to droughts. In terms of policy, meeting the GBF’s 30×30 target may benefit vulnerable BHs^4^, but specific conservation decisions should prioritize BHs in mid-latitude drylands and tropical Americas with greater drought exposure. Emphatically, these BHs are also bearing substantial social burdens, implying that conservation efforts may be constrained by local governance capacity and livelihood needs^26,47^, such as planning ecological corridors on land also valuable for agriculture or infrastructure^27^. Therefore, expanding protection networks to adapt to intensifying drought exposure entails integrating social well-being. To this end, promoting land-use intensification or de-intensification in suitable areas could simultaneously restore habitats while matching socioeconomic demands^60,61^. Moreover, strengthening cost-sharing and fiscal transfer mechanisms for conservation^62^, facilitating transboundary collaboration^63^, and recognizing conservation approaches rooted in indigenous and local communities^64^ are essential for reducing conflicts and improving governance, particularly in BHs that share habitats or water systems.

Our analyses revealed which BHs and species are most threatened by intensifying drought, thus enabling pre-emptive conservation actions. However, geographic range exposure to drought severity exceeding historical extremes may not necessarily imply local biodiversity loss. Our assessments of species drought tolerance are based on their realized climatic niche limits rather than direct physiological measurements. The fundamental niche limits of species may be wider than their realized niche limits^37,54^. Some species may buffer drought exposure through behavioral adaptations or phenotypic plasticity, such as utilizing microhabitats^48^, shifting seasonal activities^65^, adapting new habitats to alter current range^54^, or evolving physiological tolerance to drought^14^. The effects of species interactions^55^ and other climate extremes, such as heatwaves and wildfires^10,66^, may compromise exposure estimates. Furthermore, drought-threshold effect may also influence our results, which determines the drought severity (see Methods). Nevertheless, our assessment may still be a credible approximation of species exposure to potentially hazardous drought conditions, as recent unprecedented droughts have already caused fatal impacts on some dryland animals whose realized geographic range edges closely match their drought tolerance limits^15–17^. This study did not include plants and invertebrates that perform key ecosystem functions such as food provisioning and nutrient cycling^36^ because most of their global range maps are unavailable, potentially introducing taxonomic bias into the assessment of overall impacts on biodiversity. Importantly, our analysis focused on BHs defined by the diversity of endemic vascular plants, potentially overlooking certain terrestrial vertebrate diversity hotspots (such as the Amazon basin)^39,67^. To this end, we performed an additional analysis based on terrestrial vertebrate diversity hotspots (Supplementary Fig. 16), which similarly reveals and confirms that threatened terrestrial vertebrates are increasingly exposed to drought severity exceeding their historical extremes, while limiting future warming will reduce this drought exposure. Future research priorities are recommended to refine the investigation and validation of species-specific physiological drought tolerance and adaptation mechanisms across different climate scenarios, and explicitly estimate the consequences of drought exposure, including habitat range shifts and impacts on interactions and trophic dynamics across taxa in food networks^7,55,66^.

Ambitious global targets necessitate protecting critical yet vulnerable BHs and conserving species threatened by climate changes and human activities^4^. As climate warming intensifies drought severity, threatened terrestrial vertebrates in BHs face increasing drought exposure beyond their realized climate niche limits. This threat underscores the urgent need for both ambitious climate mitigation strategies to reduce drought exposure, including immediate reductions in greenhouse gas emissions, alongside proactive adaptive conservation actions. These adaptive measures must prioritize protecting, restoring, and connecting exposed regions, while crucially balancing local well-being. Prioritizing and safeguarding drought-vulnerable BHs and species holds the potential to prevent intensifying drought from undermining the achievement of global biodiversity targets.

## Methods

### Biodiversity hotspots

Conservation International (https://www.conservation.org/) has identified 36 Biodiversity Hotspots (BHs) globally (Fig. 1 and Supplementary Table 2), which are strictly defined as terrestrial regions that contain at least 1500 species of vascular plants found nowhere else on Earth and have lost at least 70% of their primary native vegetation^1–3^. These most biologically rich yet threatened regions cover only about 16% of the Earth’s terrestrial surface but support nearly half of the world’s endemic plants and terrestrial vertebrates, representing global conservation priorities in the context of accelerating biodiversity loss and limited conservation resources^1–3^.

### Climate data

We used 1851 to 2100 monthly simulations for near-surface air temperature (mean, maximum, and minimum), precipitation, surface shortwave radiation, near-surface wind speed, and surface air pressure from five Global Climate Models (GCMs) for the Coupled Model Intercomparison Project Phase 6 (CMIP6) to derive the dynamics in drought severity (see Quantifying drought severity). The updated CMIP6 models have enhanced the prediction of climatic extremes compared to their former CMIP5 models^7,68^, and have been widely applied to assess the impacts of drought on vegetation productivity^69^, biodiversity^33,35^, agricultural crops^9,12^, and socio-ecological systems^10,13^. We used five GCMs including: the Alfred Wegener Institute, Germany (AWI-CM-1-1-MR)^70^; the Beijing Climate Center, China (BCC-CSM2-MR)^71^; the European Community Earth System Model (EC-Earth3)^72^; the Marchuk Institute for Numerical Mathematics of the Russian Academy of Sciences (INM-CM5-0)^73^; and the Meteorological Research Institute, Japan (MRI-ESM2.0)^74^. These models adequately represent the ‘cold’ (INM-CM5-0; BCC-CSM2-MR), ‘intermediate’ (AWI-CM-1-1-MR; MRI-ESM2.0), and ‘warm’ (EC-Earth3) models for integrating equilibrium climate sensitivity and transient climatic response to account for the inherent variability in climate projections and uncertainty in model simulations whenever possible^75^. We resampled all data to a spatial resolution of 0.5° ( ~ 55.5 km at the equator) using bilinear interpolation^12^ to reduce scale effects in bioclimatic response assessments^76^.

To explore the impact of future anthropogenic climate policy decisions on drought exposure estimates, all GCMs outputs include three future climate scenarios that integrate Representative Concentration Pathways (RCPs) and Shared Socioeconomic Pathways (SSPs), including SSP126, SSP245, and SSP585^7,77^. SSP126 is an optimistic low-emission scenario (with global mean warming below 1.8 °C above pre-industrial levels by the end of the 21st century), representing an international commitment to achieving the Paris Agreement goals through strong strategies to mitigate climate change^7^. SSP245 represents an intermediate-emission scenario, with socioeconomic trends largely consistent with historical trajectories^78^. Under SSP245, global mean warming is projected to reach 2.7 °C by 2100, which is the most likely outcome under current policies^7,78^. SSP585 is a pessimistic high-emission scenario (projected mean warming of 4.4 °C), characterized by social development that heavily relies on fossil fuel-intensive economies^77,78^. Although recent developments in alternative energy and other climate mitigation measures render high-emission scenarios like SSP585 less realistic^7,79^, we still considered SSP585 because it encompasses all possible future anthropogenic radiative forcing^79^.

To validate the performance of GCMs from CMIP6 for simulating and projecting climate change, we examined the correlation between simulated climate data from GCMs and reanalyzed climate data derived from meteorological stations and satellites (WorldClim 2.1 and ERA5-Land). The strong positive relationship (most Pearson’s correlation coefficients (*r*) > 0.8, *p* < 0.001, Supplementary Fig. 17) between the simulated and observed data confirms that the CMIP6 models performed well.

### Geographic range data of terrestrial vertebrates

We used species occurrence range polygons from the International Union for Conservation of Nature (IUCN, v.6.3) for mammals, reptiles, and amphibians and BirdLife International (BirdLife, v.8.0) for birds. These expert-validated species ranges provide global geographic distribution information for almost all terrestrial vertebrates and are likely a better alternative to point-locality data for species coverage^80^. However, such data are susceptible to commissions, omissions, and biases^81^. We used only the ranges of year-round resident terrestrial vertebrates (primarily excluding migratory birds), and omitted vertebrates in both marine and terrestrial habitats (e.g., *Laridae*, *Phocidae*, and *Cheloniidae*), to maintain consistency in drought exposure assessments^39^. We focused primarily on vulnerable, endangered, and critically endangered species threatened with extinction, as identified by IUCN^40^. The biases in classification criteria of species threat levels may introduce uncertainty into this study^40^. By April 2024, we had collected geographic range data for 28,050 species of terrestrial vertebrates occurring in BHs, representing 83.0% of currently known terrestrial vertebrates globally, including 9952 birds, 4556 mammals, 7413 reptiles, and 6129 amphibians; and 5845 species of threatened terrestrial vertebrates, including 1335 threatened birds, 961 threatened mammals, 1260 threatened reptiles, and 2289 threatened amphibians.

We gridded the collected species range polygons into 0.5° equal-area grid cells to match the resolution of climate data to capture the local climate variation across species ranges without inducing false species presence wherever possible^76,81^. Note that marginal grid cells with lower overlap with the species range may inflate species’ niche limits. For instance, some species bordering deserts are more likely to disperse to humid refuges rather than remain at the drier range margins during droughts^15,18^. Therefore, for species with an overall range polygon larger than one grid cell area, we considered the species presence in a grid cell only if its range occupied more than 10% of this grid cell. Nevertheless, we examined the sensitivity of species-specific historical drought severity extremes to overlap thresholds of 1%, 5%, 20%, and 50%, respectively. The strong correlation (Pearson’s *r* > 0.95, *p* < 0.001, Supplementary Fig. 18) between results from the 10% overlap threshold and other thresholds indicates that historical drought severity extremes for most species are insensitive to overlap thresholds. Given that narrow-range species with limited dispersal capacity (particularly many threatened and endemic species) are more vulnerable to climate extremes^35,82^, we did not exclude species with an overall range polygon smaller than one grid cell area and retained any grid cells they occupied, although this may be affected by the spatial scale explored^76,81^.

### Protected area coverage

To assess the coverage of protected areas in BHs, we utilized polygons from the World Database on Protected Areas (WDPA, downloaded in December 2024 from www.protectedplanet.net/). Notably, we merged the full 2016 WDPA dataset with the 2024 WDPA dataset to address the outdated coverage of protected areas in China and compiled data for incompletely protected areas for India and Turkey using the OpenStreetMap database (downloaded in December 2024 from http://www.openstreetmap.org/). We excluded ‘Other Effective Conservation Measures (OECMs)’ beyond protected areas, because most countries have not yet provided data on OECMs to the WDPA^83^. We consider a 0.5° resolution grid cell to be protected if at least 20% of its area is covered by protected areas^37^.

### Quantifying drought severity

We used the standardized precipitation-evapotranspiration index (SPEI) to quantify drought severity under climate change. SPEI tracks deficits and surpluses between precipitation and evapotranspiration across multiple time scales, characterizing the relative deviation of a drought condition from the long-term average climate, and is considered a robust indicator for monitoring and analyzing drought dynamics^8,12,84^. SPEI can be quantified as the difference between precipitation and potential evapotranspiration (PET), namely, climatic water balance^84^. PET formulation usually employs the Penman-Monteith equation (PET_PM)^85^ and one of its variants that accounts for decreased evapotranspiration due to increased surface resistance driven by rising atmospheric CO₂ concentrations (PET_CO_2_)^86^, which are respectively parameterized as:

1 $${{{\rm{PET}}}}\_{{{\rm{PM}}}}=\frac{0.408\varDelta \left({R}_{n}-G\right)+\gamma \frac{900}{{T}_{{avg}}+273}{u}_{2}\left({e}_{s}-{e}_{a}\right)}{\varDelta+\gamma \left(1+0.34{u}_{2}\right)}$$

2 $${{{\rm{PET}}}}\_{{{{\rm{CO}}}}}_{2}=\frac{0.408\varDelta \left({R}_{n}-G\right)+\gamma \frac{900}{{T}_{{avg}}+273}{u}_{2}\left({e}_{s}-{e}_{a}\right)}{\varDelta+\gamma \left(1+\left(0.34+2.4\times {10}^{-4}\left(\left[{{{{\rm{CO}}}}}_{2}\right]-300\right)\right){u}_{2}\right)}$$

where $\varDelta$ is the gradient of the saturated vapor pressure curve, ${R}_{n}$ is the net radiation at the crop surface, $G$ is the soil heat flux density, $\gamma$ is the psychrometric constant, ${T}_{{avg}}$ is the near-surface temperature, ${u}_{2}$ is the near-surface wind speed, ${e}_{s}-{e}_{a}$ is the saturated and actual vapor pressure deficit, these parameters can be derived from the climate variables collected from GCMs of CMIP6. Atmospheric CO₂ concentration ([CO_2_]) grid data were obtained from the GFDL-ESM4 model in CMIP6, which show a strong correlation with observations from the Global Monitoring Laboratory (Pearson’s *r* > 0.99, *p* < 0.001, Supplementary Fig. 19). Notably, the CO₂ effect on surface resistance in the PET_CO_2_ was modeled on non-water-limited surfaces, and applying it to drylands may underestimate PET increases^86^. Previous research on dryland expansion under climate warming suggests that PET in drylands should fall between estimates with and without CO_2_ effect^13^. Therefore, we estimated PET using the average of the PET_PM and PET_CO_2_ models for global drylands (as defined by UNEP-WCM), while employing the PET_CO_2_ model for other regions. The SPEI is further calculated by transforming the empirical probability of the difference between monthly precipitation and PET into the standard normal distribution function^84^. We chose the 12-month SPEI scale to analyze drought dynamics^12,84^, as ecological processes such as growth, reproduction, and migration of many species follow interannual cycles^65,66^. We calculated the monthly SPEI for the global terrestrial (excluding Antarctica) domain from 1851 to 2100 for each GCM and reported the median and standard deviation of SPEI across models. Note that SPEI primarily characterizes meteorological drought^84^, and incorporating other drought perspectives (such as soil drought, hydrological drought, and ecological drought) can refine our understanding of the overall impacts of intensifying drought on biodiversity^8,13^.

The impacts of droughts on ecosystems depend on the strength of drought frequency, duration, and intensity, as well as their combination (that is, drought severity)^8,9,56^. We categorized a drought event as an occurrence with the SPEI value at least two consecutive months below the drought threshold (SPEI < –1.5), which corresponds to the lower threshold for severe drought as defined by the commonly used U.S. Drought Monitor classification scheme^9,87^. Drought frequency is defined as the number of non-consecutive drought events occurring in a given time period; drought duration is defined as the consecutive number of months for each drought event; drought intensity is defined as the absolute value of the SPEI value associated with drought months; and drought severity is further measured as the cumulative sum of all drought intensities in a single drought event (Supplementary Fig. 20). It must be stressed that when SPEI values within a time series cluster near drought threshold, such methods may amplify drought severity by overestimating drought frequency. Based on the median of SPEI time series across GCMs, we calculated the total drought severity for the historical baseline (pre-industrial, 1851 to 1900), recent (1975 to 2024), and future (2051 to 2100) periods using a 50-year timescale, further normalized to relative drought severity, in order to assess drought dynamics since the onset of large-scale anthropogenic warming. The patterns of our quantified drought severity are largely aligned with other studies of drought expansion^7–12^. Notably, the dominant cycles of extreme weather are becoming increasingly unstable under global warming^88^, and historical megadroughts with recurrence intervals longer than 50 years may still shape ecosystems^89^.

### Defining species-specific historical drought severity extremes

The intensification of drought severity will force organisms to endure unprecedented environmental stresses^14,15,18^, but the exact impacts are contingent upon species-specific drought vulnerability^19,39^. However, physiological data on drought vulnerability for most species remain unavailable^39^. In fact, a species’ geographic distribution results from long-term adaptation to climatic conditions^54^, and the climatic extremes experienced across its realized distribution likely represent its climatic niche limits^37,54,90,91^. Therefore, we applied methods similar to studies on temperature extremes^35,37^, approximating species-specific drought tolerance limits by estimating historical drought severity extremes within species’ realized niches. Specifically, species-specific historical drought severity extremes were calculated as the 99th percentile of drought severity experienced across all grid cells within each species’ expert-validated global distribution during the historical baseline (Supplementary Figs. 1 and 21). This percentile-based method reduces the effects of extreme outliers and overestimated species ranges^35,81^. Importantly, considering recent droughts have already imposed significant impacts on ecosystems^15–17,21,66^, we use the pre-industrial period (1851 to 1900, characterized by relatively stable climate conditions) as the historical baseline. It must be underlined that species drought tolerance limits estimated through realized niches may be conservative, as species may not have reached their actual physiological limits in the pre-industrial period. Furthermore, some species’ range maps may underestimate the extent of their climatic niche due to limited long-term historical records for calibration^80^. Phenotypic plasticity of species to drought stress and species interactions may lead to misjudgments of drought tolerance limits by applying historical drought severity extremes^14,55^.

### Estimating geographic range exposure

Using species-specific historical drought severity extremes, we assessed the precise locations where intensifying drought threatens biodiversity. We mapped drought exposure for each species by identifying whether recent or future drought severity in grid cells across its current geographic range exceeds its historical extremes. Drought exposure of species was synthesized in two complementary metrics: (1) the average percentage of current geographic range in BHs exposed to drought severity exceeding historical extremes across all species; and (2) the percentage of species exposed to drought severity exceeding their historical extremes across at least half ( ≥ 50%) of their current geographic range in BHs, providing a comparable summary indicator of biodiversity range loss^36^. Given the uncertainty in future socioeconomic development pathways, we also employed hierarchical clustering analysis^92^ to create an ensemble of projections according to exposure estimates under three scenarios (SSP126, SSP245, and SSP585) for each BH, thereby more conservatively identifying which BHs are vulnerable to drought exposure in the future. Notably, complex ecological processes, such as species utilizing climate refuges or dispersing into new habitats, may alter exposure estimates^54,55^.

### Spatial scale effect

We assessed the species exposure to intensifying drought using climate model data at 0.5° resolution, aiming to balance the need to capture spatial climate details while avoiding false species presence^76,81^. However, 0.5° grid cells may contain considerable spatial climate heterogeneity, causing discrepancies between our analyzed drought conditions and the local microclimates that influence organism survival^93^. To this end, we examined the relationship between drought exposure in each grid cell and its internal spatial climate heterogeneity. Spatial climate heterogeneity was calculated as the standard deviation of the 1-km resolution aridity index within each grid cell, reflecting regional long-term climate characteristics^94^. Habitats with higher spatial climate heterogeneity in BHs exhibit more moderate drought exposure (Supplementary Fig. 22), indicating that our findings still identified those habitats with more diverse climatic conditions (such as mountain communities) as better buffers against drought threats. Importantly, while the use of finer-resolution climate data may introduce biases in estimates of drought exposure, it does not change the central conclusion that species are increasingly exposed to drought severity beyond their realized niche limits, and that limiting future warming can reduce exposure. The reason is that drought exposure tends to be greater in habitats with relatively low spatial climate heterogeneity. Nevertheless, adopting finer-scale climate data that more closely reflects the conditions actually experienced by organisms may improve the accuracy with which the impacts of intensifying drought on biodiversity are represented^76,81^.

### Reporting summary

Further information on research design is available in the Nature Portfolio Reporting Summary linked to this article.

## Supplementary information

Supplementary information

Reporting Summary

Transparent Peer Review file

## Source data

Source Data

## Data Availability

The historical, recent, and future relative drought severity data generated in this study have been deposited in the Institute of Tibetan Plateau Research, Chinese Academy of Sciences, with open access 10.11888/Terre.tpdc.302324. Geographic range data for biodiversity hotspots is available at 10.5281/zenodo.3261807. CMIP6 climate data is available at https://metagrid.esgf-west.org/search/cmip6/. Geographic distribution data for birds is available at https://datazone.birdlife.org/contact-us/request-our-data (BirdLife v.8.0), and for mammals, reptiles, and amphibians at https://www.iucnredlist.org/resources/spatial-data-download (IUCN v.6.3). Map for drylands is available at https://data-gis.unep-wcmc.org/portal/home/item.html?id=789fcac8959943ab9ed7a225e5316f08. Protected area coverage data is available at www.protectedplanet.net and www.openstreetmap.org/. Human footprint data in 2020 is available at 10.5061/dryad.ttdz08m1f. Source data are provided with this paper.

## Code availability

The related data analyses and modeling were performed using Python 3.8. The code is publicly available at 10.11888/Terre.tpdc.302324.
