# Supplementary material for: Limiting future warming reduces drought exposure for terrestrial vertebrates
Source: Nat Commun. 2026 May 14;17:6443. doi: 10.1038/s41467-026-73229-3 (PMC13376502; doi:10.1038/s41467-026-73229-3)
Supplement: Supplementary file 1 — Supplementary information [file 41467_2026_73229_MOESM1_ESM.pdf]

# Supplementary information

# Limiting future warming reduces drought exposure for terrestrial vertebrates


## Authors and Affiliations

Yuchuan He[1,2], Jian Sun[1,2*], Yanqiang Wei[3], Yanxu Liu[4], Michael E. Meadows[5,6], Josep Peñuelas[7,8]

[1]State Key Laboratory of Tibetan Plateau Earth System, Environment and Resources (TPESER), Institute of Tibetan Plateau Research, Chinese Academy of Sciences, Beijing 100101, China.

[2]University of Chinese Academy of Sciences, Beijing 100049, China.

[3]State Key Laboratory of Cryospheric Science and Frozen Soil Engineering, Key Laboratory of Remote Sensing of Gansu Province, Northwest Institute of Eco-Environment and Resources, Chinese Academy of Sciences, Lanzhou 730000, China.

[4]State Key Laboratory of Earth Surface Processes and Hazards Risk Governance, Faculty of Geographical Science, Beijing Normal University, Beijing 100875, China.

[5]School of Geography and Oceanographic Sciences, Nanjing University, Nanjing 210023, China.

[6]Department of Environmental & Geographical Science, University of Cape Town, Rondebosch 7701, South Africa.

[7]CREAF, Cerdanyola del Valles, Barcelona 08193, Spain.

[8]CSIC, Global Ecology Unit CREAF-CSIC-UAB, Bellaterra, Barcelona 08193, Spain.

* Corresponding authors: J.S.: sunjian@itpcas.ac.cn

**Sections**

**1. Supplementary Figures 1-22**

**2. Supplementary Tables 1 and 2**

## Supplementary Figures

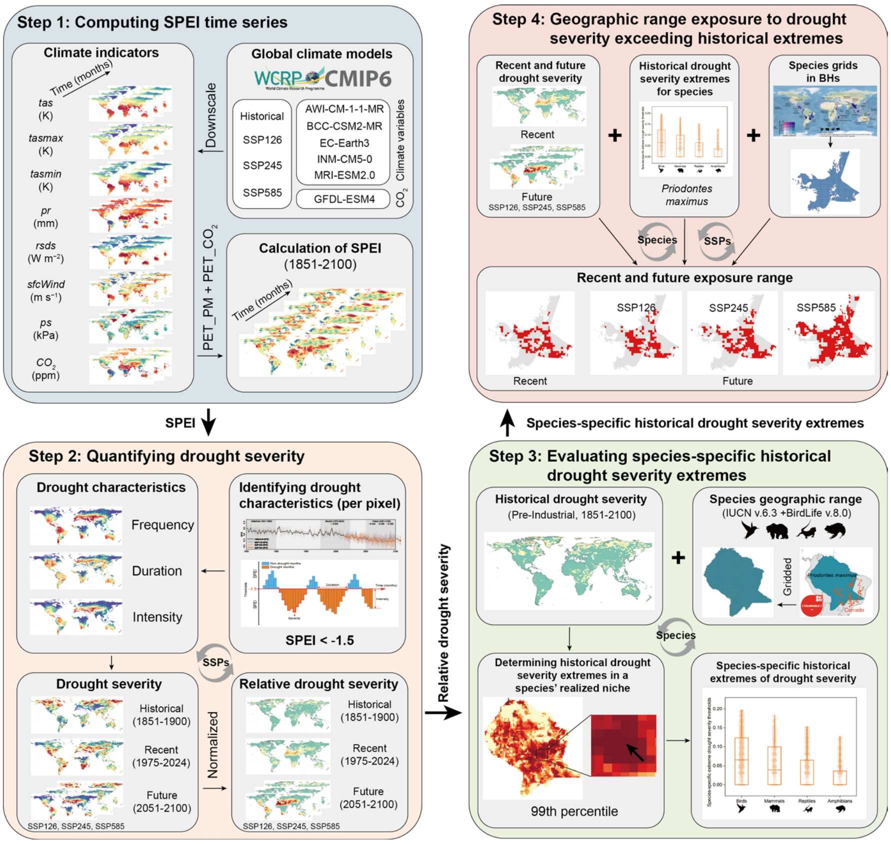


**Supplementary Fig. 1 Overview of methods used for assessing species geographic range exposure to drought severity beyond realized niche limits.** Illustration using the geographic range of *Priodontes maximus* as a vulnerable species found in the Cerrado. **Step 1:** calculate monthly time series of the standardized precipitation-evapotranspiration index (SPEI) using GCMs data from CMIP6, where *tas* represents the monthly mean near-surface air temperature, *tasmax* represents the monthly maximum near-surface air temperature, *tasmin* represents the monthly minimum near-surface air temperature, pr represents the monthly total precipitation, *rsds* represents the monthly total surface shortwave radiation, *sfcWind* represents the monthly mean near-surface wind speed, *ps* represents the monthly mean surface air pressure, and $CO_2$ represents atmospheric carbon dioxide concentration; **Step 2:** quantify drought severity with a threshold of SPEI < –1.5; **Step 3:** evaluate species-specific historical drought severity extremes based on historical realized climate niche limits; **Step 4:** assess species geographic range exposure to drought severity exceeding historical extremes.

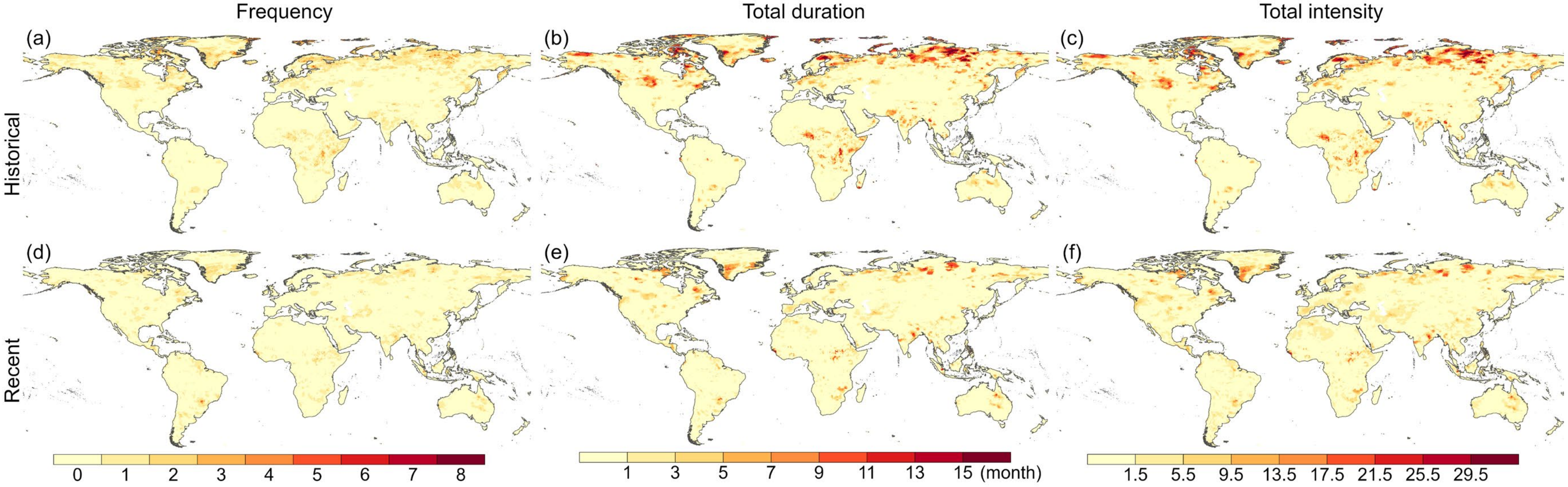


**Supplementary Fig. 2 Global terrestrial drought characteristics in historical (1851 to 1900) and recent (1975 to 2024) periods. a-c**, Frequency (**a**), total duration (**b**), and total intensity (**c**) of drought events for historical period. **d-f**, Frequency (**d**), total duration (**e**), and total intensity (**f**) of drought events for the recent period. Source data are available at https://doi.org/10.11888/Terre.tpdc.302324.

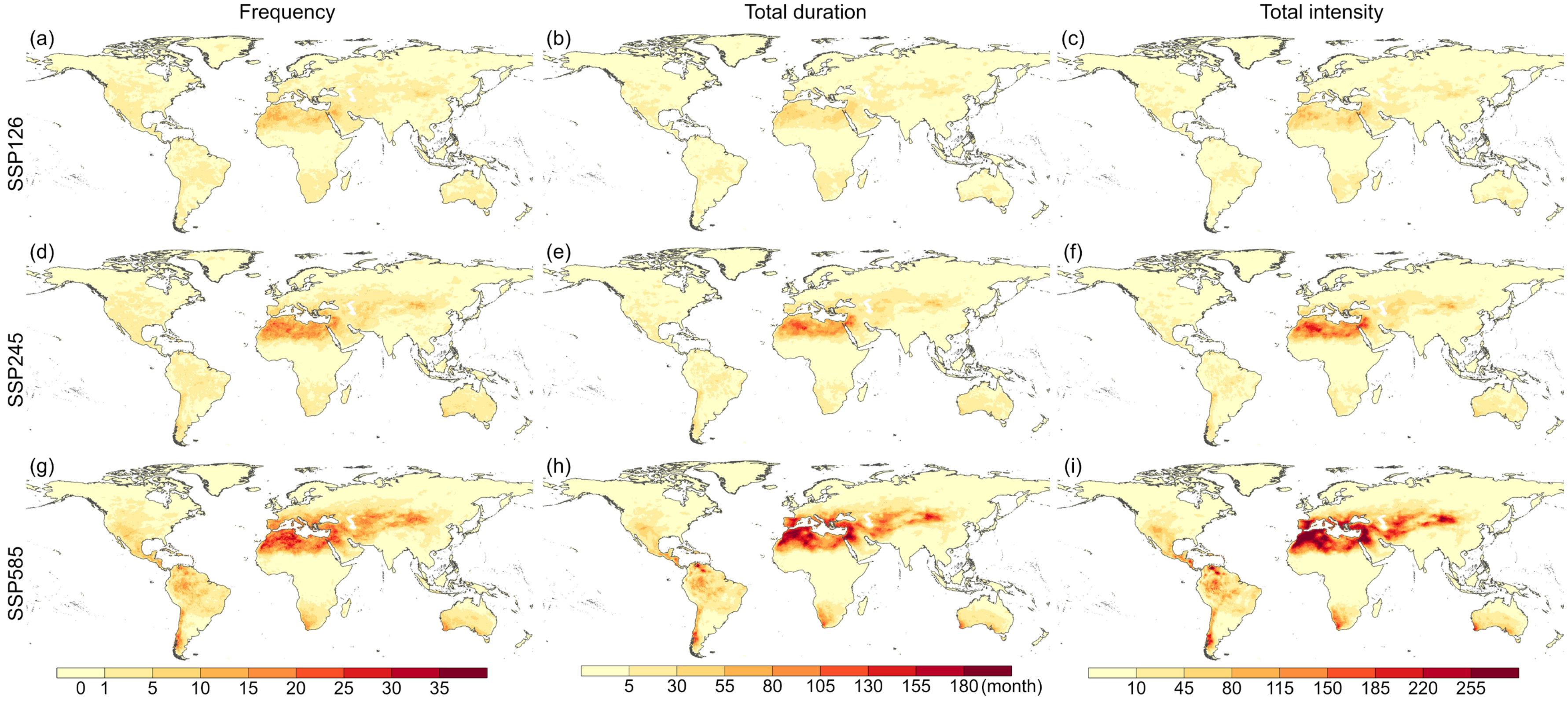


**Supplementary Fig. 3 Global terrestrial drought characteristics in future (2051 to 2100) periods. a-c**, Frequency (**a**), total duration (**b**), and total intensity (**c**) of drought events under Shared Socioeconomic Pathway 1–2.6 (SSP126). **d-f**, Frequency (**d**), total duration (**e**), and total intensity (**f**) of all drought events under SSP245. **g-i**, Frequency (**g**), total duration (**h**), and total intensity (**i**) of all drought events under SSP585. Source data are available at https://doi.org/10.11888/Terre.tpdc.302324.

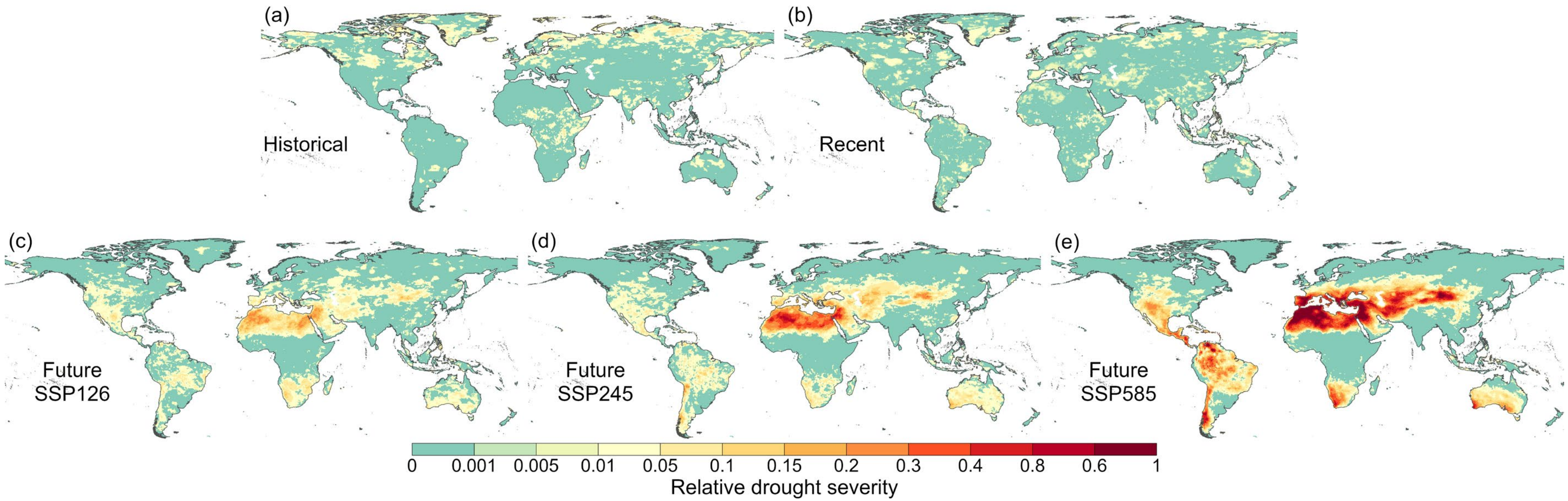


**Supplementary Fig. 4 Global terrestrial relative drought severity in historical, recent, and future periods. a**, Relative drought severity in the historical period. **b**, Recent relative drought severity in the recent period. **c-e**, Relative drought severity under Shared Socioeconomic Pathway 1–2.6 (SSP126) (**c**), SSP245 (**d**), and SSP585 (**e**). Source data are available at https://doi.org/10.11888/Terre.tpdc.302324.

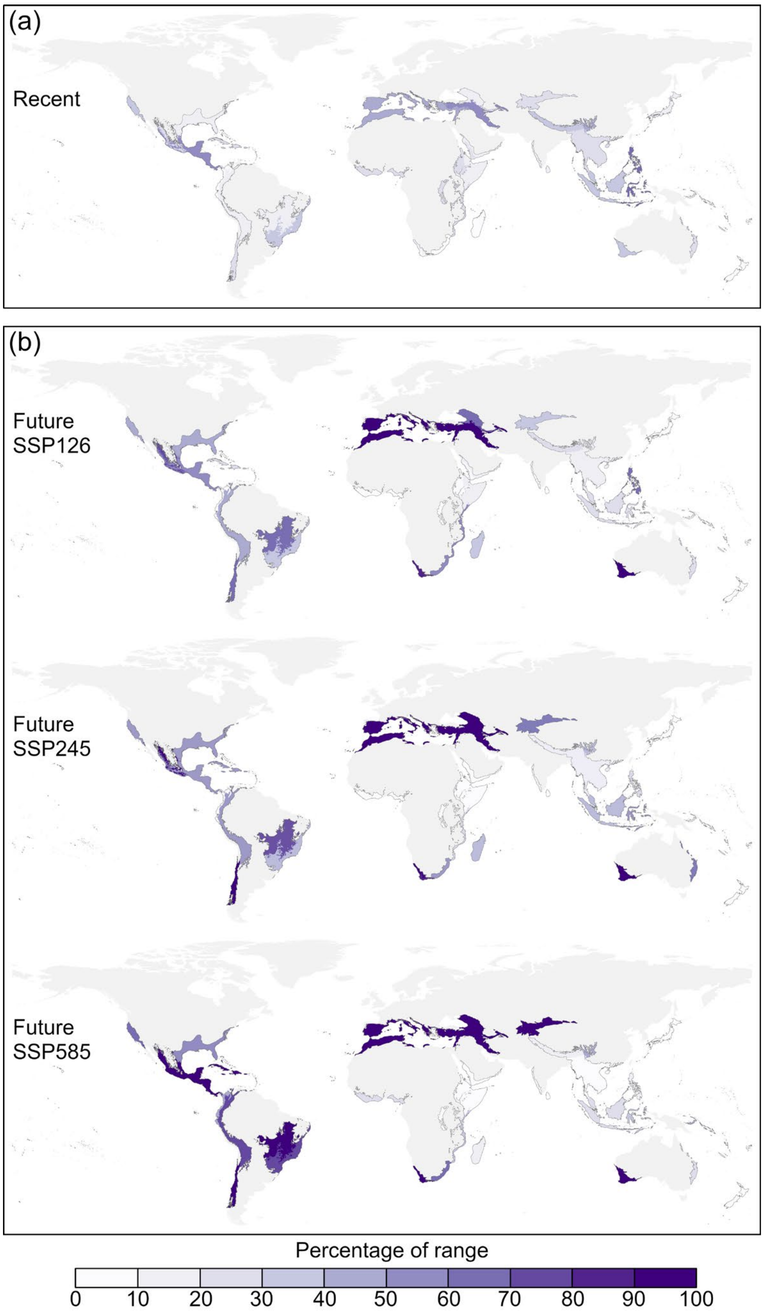


**Supplementary Fig. 5 Percentage of range in BHs where drought severity exceeds historical levels. a,** Percentage of range in the recent period. **b,** Percentage of range under Shared Socioeconomic Pathway 1–2.6 (SSP126), SSP245, and SSP585. Source data are provided as a Source Data file.

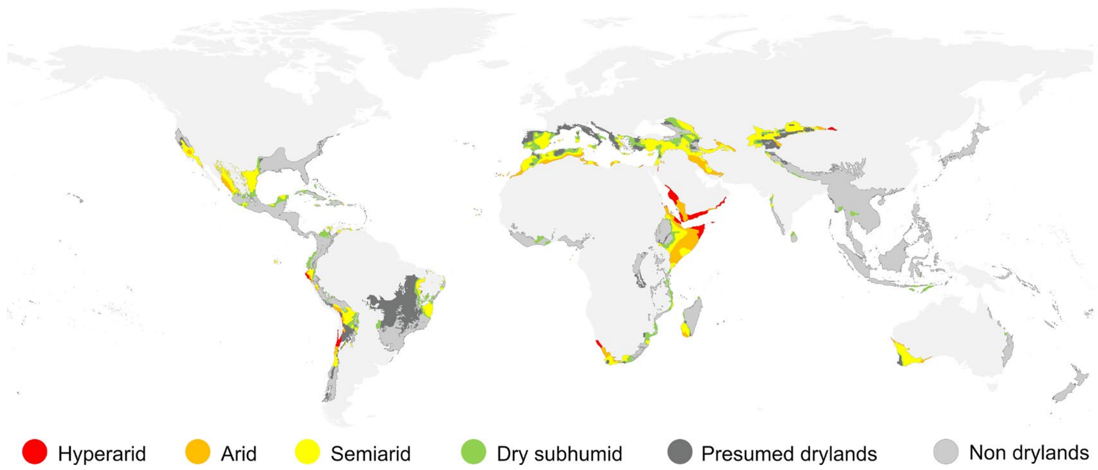


**Supplementary Fig. 6 Map of drylands in BHs.** Mapped using dryland spatial data from UNEP-WCMC (2007) (https://data-gis.unep-wcmc.org/portal/home/item.html?id=789fcac8959943ab9ed7a225e5316f08).

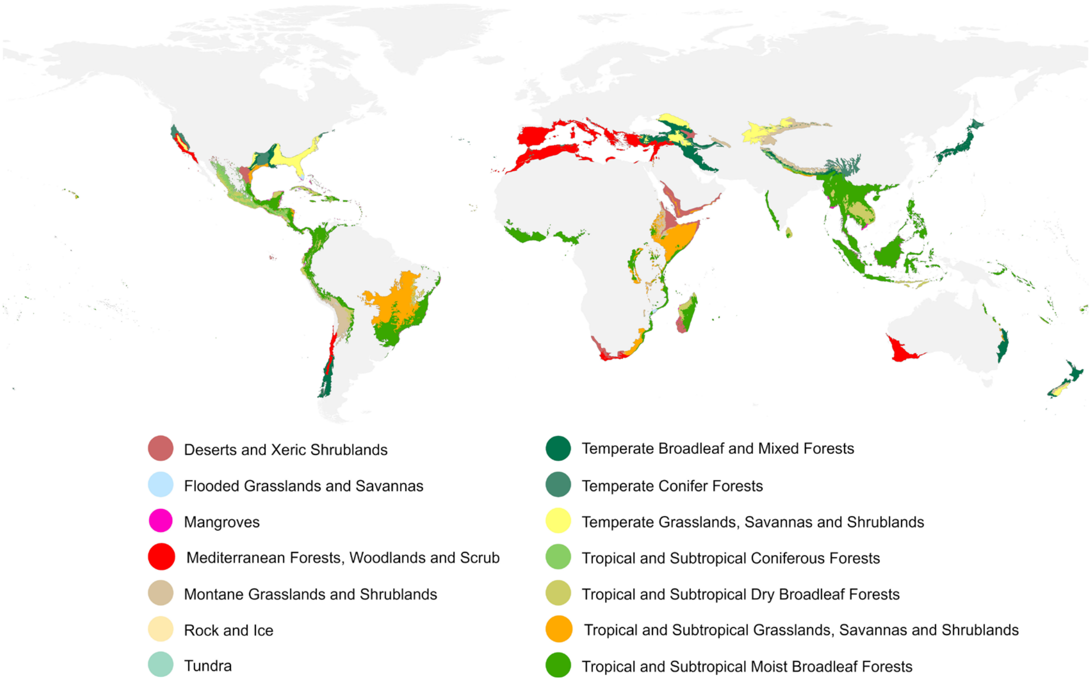


**Supplementary Fig. 7 Map of biomes in BHs.** The map of biomes is from the One Earth Bioregions framework (https://www.oneearth.org/bioregions-2023).

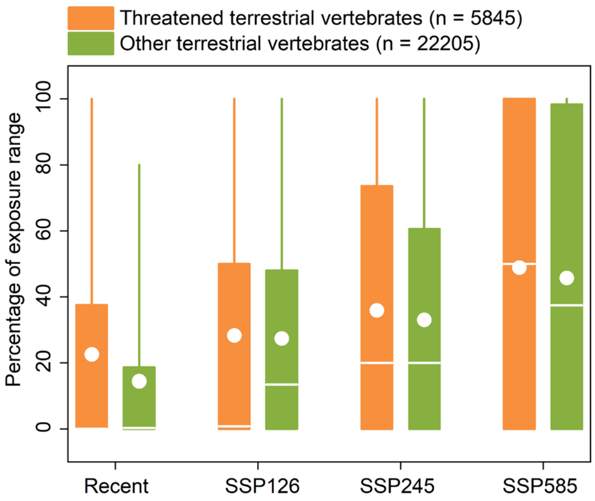


**Supplementary Fig. 8 Comparison of the percentage of exposure range to drought severity exceeding historical extremes between threatened terrestrial vertebrates and other terrestrial vertebrates in BHs.** The number *n* represents the number of species. The box plot shows the median and the 25th and 75th percentiles, the whiskers representing the 5th and 95th percentiles, and the white dots indicating average values. Source data are provided as a Source Data file.

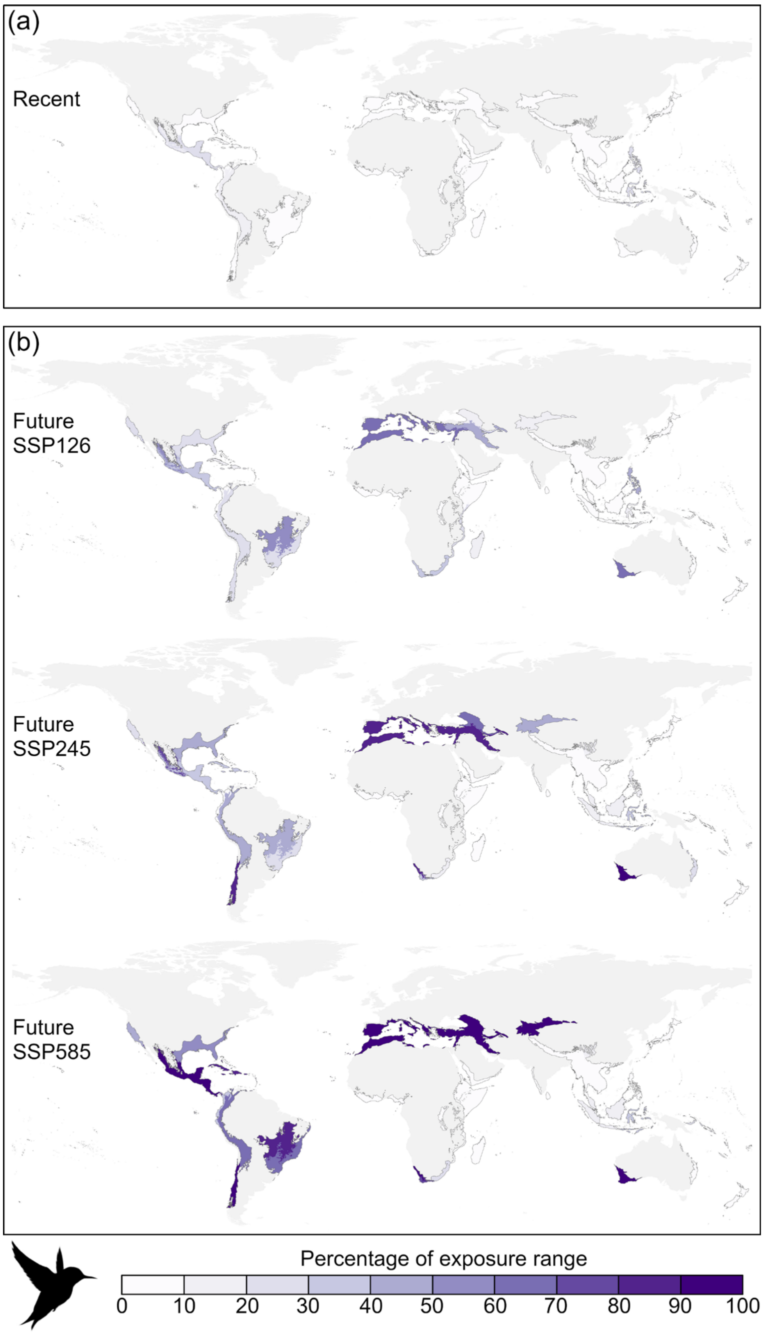


**Supplementary Fig. 9 Spatial patterns of average percentage of exposure range to drought severity exceeding historical extremes for threatened birds in BHs. a,** Average percentage of exposure range in the recent period. **b,** Percentage of exposure range under Shared Socioeconomic Pathway 1–2.6 (SSP126), SSP245, and SSP585. Source data are provided as a Source Data file.

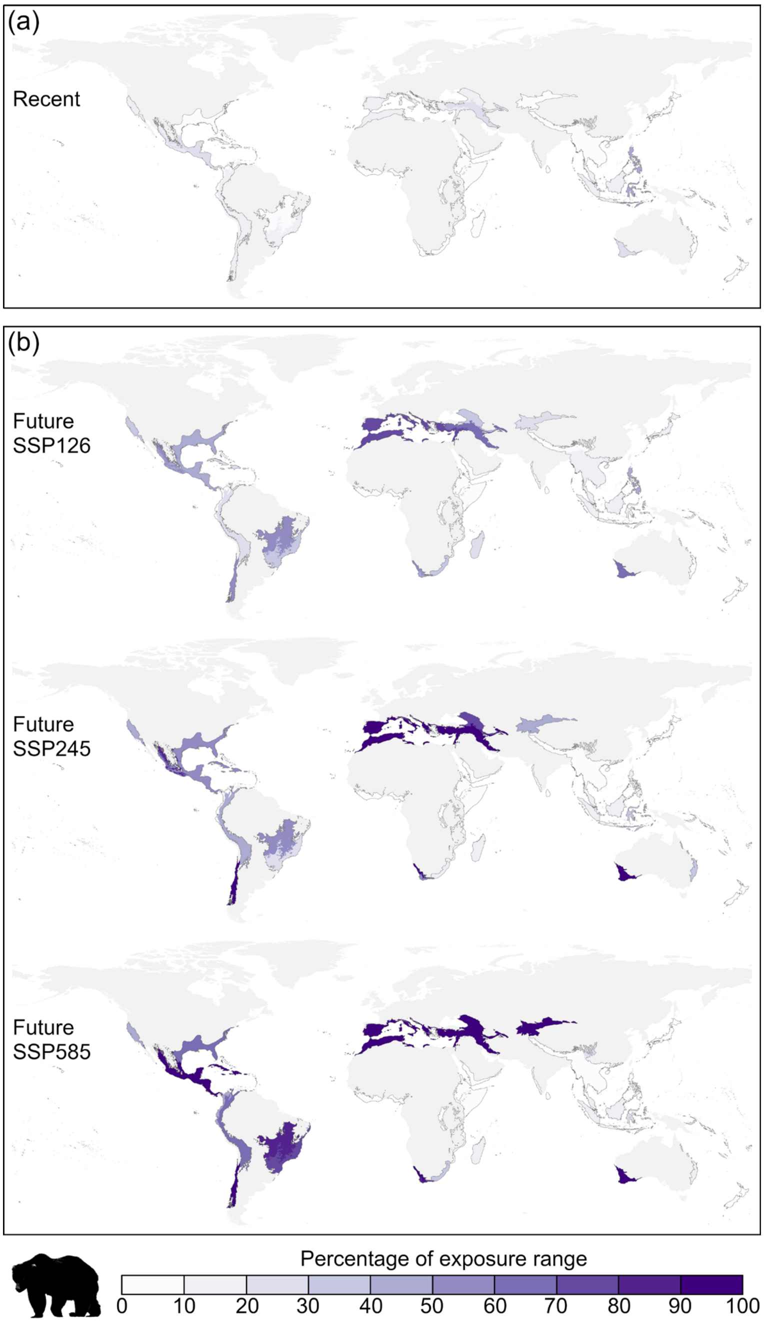


**Supplementary Fig. 10 Spatial patterns of average percentage of exposure range to drought severity exceeding historical extremes for threatened mammals in BHs. a,** Average percentage of exposure range in the recent period. **b,** Percentage of exposure range under Shared Socioeconomic Pathway 1–2.6 (SSP126), SSP245, and SSP585. Source data are provided as a Source Data file.

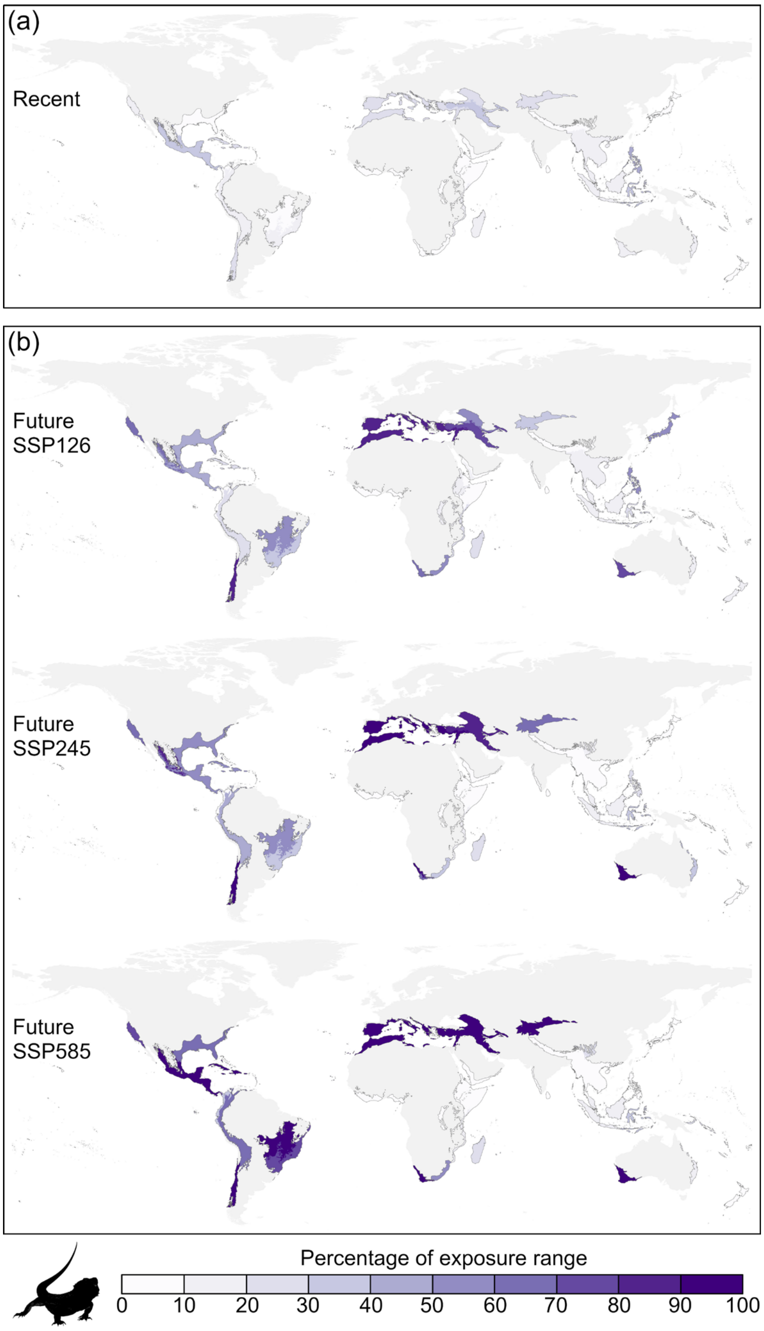


**Supplementary Fig. 11 Spatial patterns of average percentage of exposure range to drought severity exceeding historical extremes for threatened reptiles in BHs. a,** Average percentage of exposure range in the recent period. **b,** Percentage of exposure range under Shared Socioeconomic Pathway 1–2.6 (SSP126), SSP245, and SSP585. Source data are provided as a Source Data file.

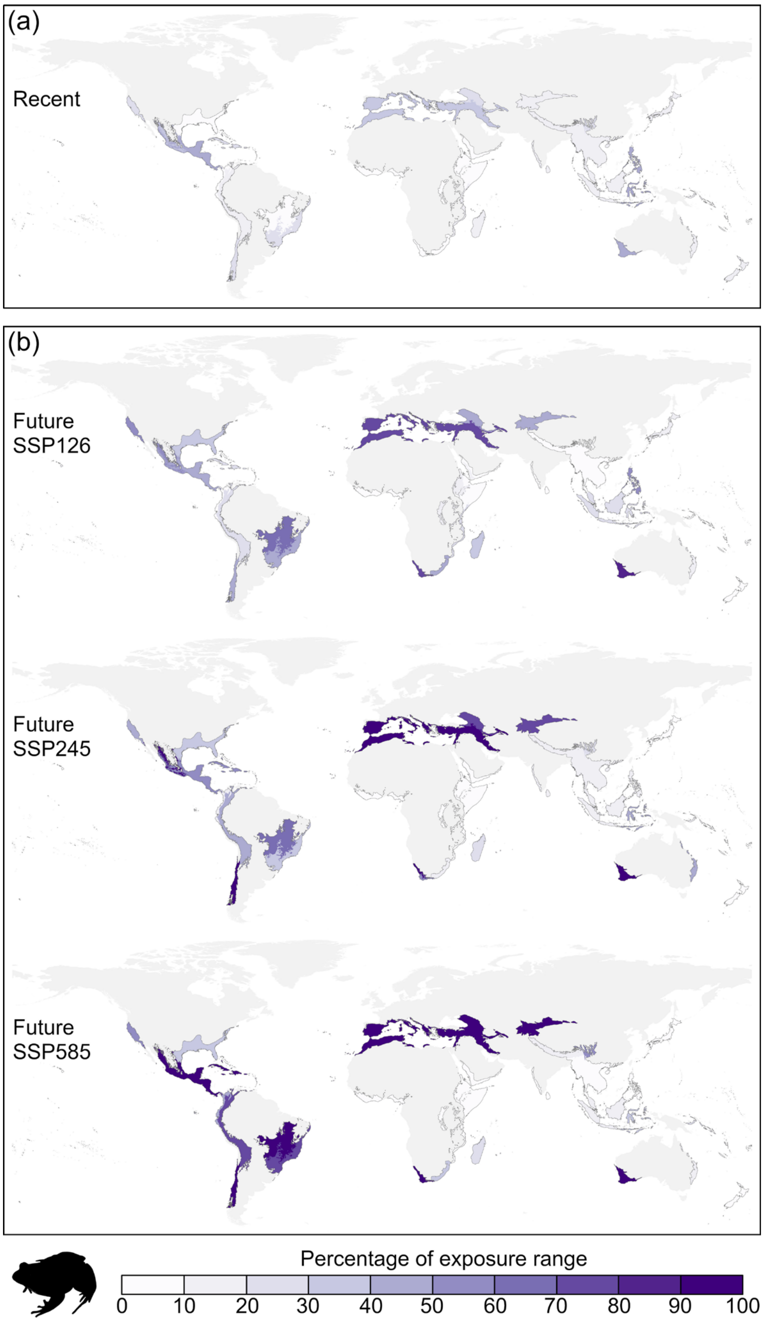


**Supplementary Fig. 12 Spatial patterns of average percentage of exposure range to drought severity exceeding historical extremes for threatened amphibians in BHs. a,** Average percentage of exposure range in the recent period. **b,** Percentage of exposure range under Shared Socioeconomic Pathway 1–2.6 (SSP126), SSP245, and SSP585. Source data are provided as a Source Data file.

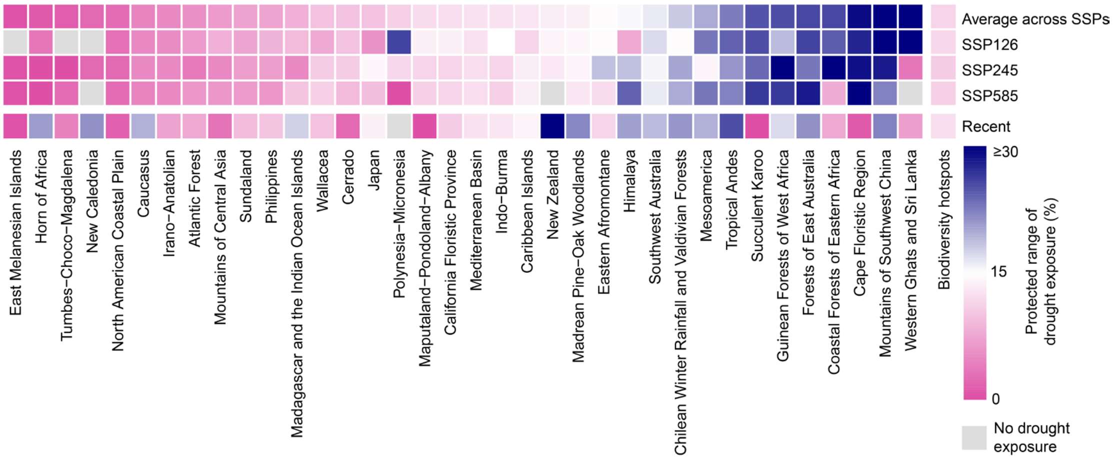


**Supplementary Fig. 13 Percentage of currently protected range for habitats facing drought exposure in each BH in recent and future periods.** Gray indicates that no species' geographic ranges in the BH are exposed to drought severity exceeding their historical extremes. SSP126: Shared Socioeconomic Pathway 1 – 2.6; SSP245: Shared Socioeconomic Pathway 2 – 4.5; SSP585: Shared Socioeconomic Pathway 5 – 8.5. Source data are provided as a Source Data file.

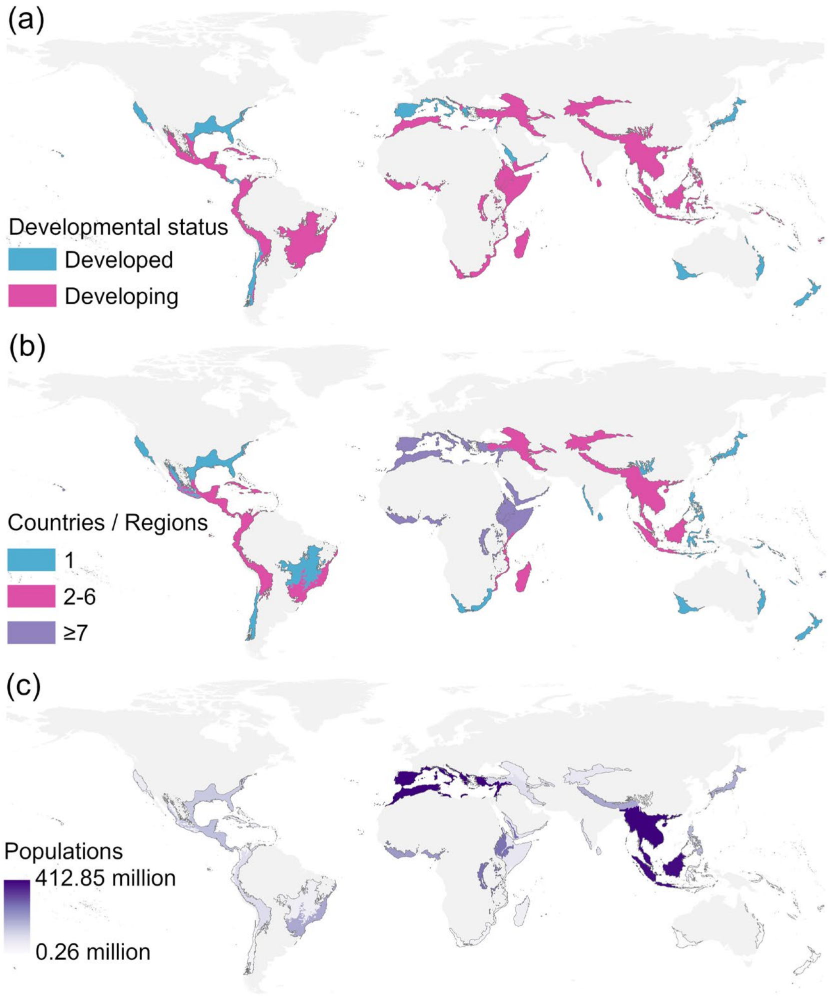


**Supplementary Fig. 14 Socioeconomic context in BHs. a,** The developmental status of countries in BHs. The national developmental statuses are determined by the World Bank based on national incomes (https://data.worldbank.org.cn/?locations=XD-XO-XL). **b,** The number of countries or regions spanned by BHs. **c,** The populations in BHs (2022), data obtained from the Oak Ridge National Laboratory (https://landscan.ornl.gov, 2022). Source data are provided as a Source Data file.

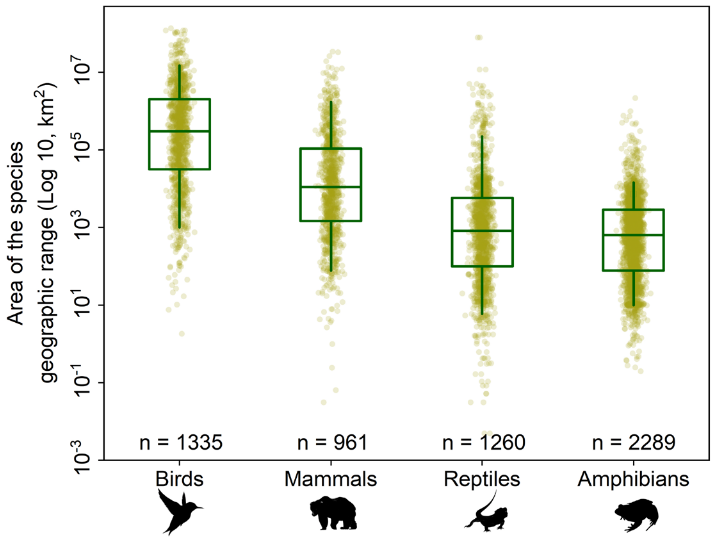


**Supplementary Fig. 15 Comparison of the size of geographic ranges for threatened birds, mammals, reptiles, and amphibians.** Geographic ranges are shown as log10 values. The number *n* represents the number of threatened species. The box plot shows the median and the 25th and 75th percentiles, with whiskers representing the 5th and 95th percentiles. Source data are provided as a Source Data file.

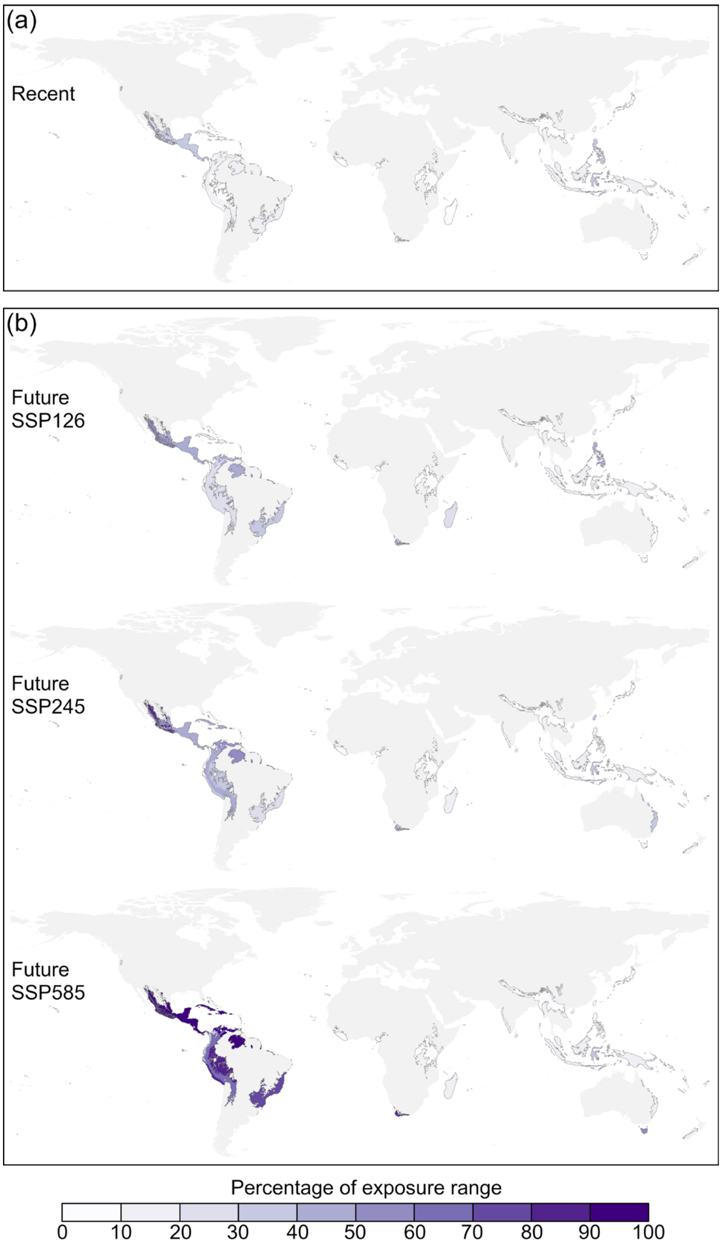


**Supplementary Fig. 16 Species geographic range exposure to drought severity exceeding historical extremes in terrestrial vertebrate diversity hotspots.** Spatial patterns of average percentage of exposure range to drought severity exceeding historical extremes for threatened terrestrial vertebrates in terrestrial vertebrate diversity hotspots under recent (**a**) and three future scenarios (**b**). SSP126: Shared Socioeconomic Pathway 1 – 2.6; SSP245: Shared Socioeconomic Pathway 2 – 4.5; SSP585: Shared Socioeconomic Pathway 5 – 8.5. The map of terrestrial vertebrate diversity hotspots was downloaded from https://doi.org/10.5061/dryad.sn02v6xf9. The analysis of drought exposure was performed on the geographic ranges of 5,209 threatened terrestrial vertebrates occurring in terrestrial vertebrate diversity hotspots, including 1,136 birds, 900 mammals, 1,026 reptiles, and 2,147 amphibians. Source data are provided as a Source Data file.

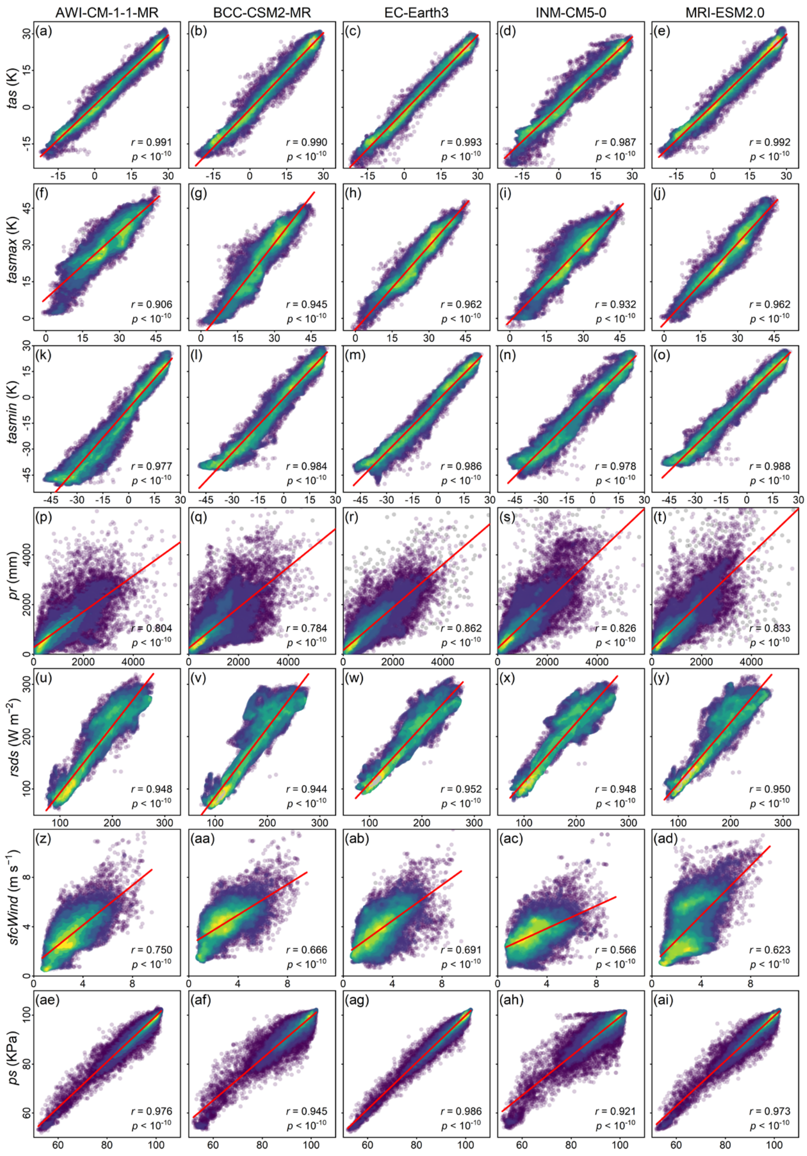


**Supplementary Fig. 17 Validation of climate variables from five GCMs of CMIP6 for the historical period (1970 to 2000) using WorldClim 2.1 and EAR5-Land datasets. a-e,** Validation of average temperatures (*tas*) using WorldClim 2.1; **f-j,** validation of maximum temperatures (*tasmax*) using WorldClim 2.0; **k-o,** validation of minimum temperatures (*tasmin*) using WorldClim 2.1; **p-t,** validation of precipitation (*pr*) using WorldClim 2.1; **u-y,** validation of surface shortwave radiation (*rsds*) using WorldClim 2.1; **z-ad,** validation of near-surface wind speed (*sfcWind*) using WorldClim 2.1; and **ae-ai,** validation of surface air pressure (*ps*) using EAR5-Land. Redder colors indicate higher point densities. Red lines represent linear regressions; Pearson's correlation coefficients (*r*) and two-sided unadjusted *p* values are provided. WorldClim 2.1 data were obtained from https://www.worldclim.org/. ERA5-Land data were obtained from the Copernicus Climate Data Store (https://cds.climate.copernicus.eu/).

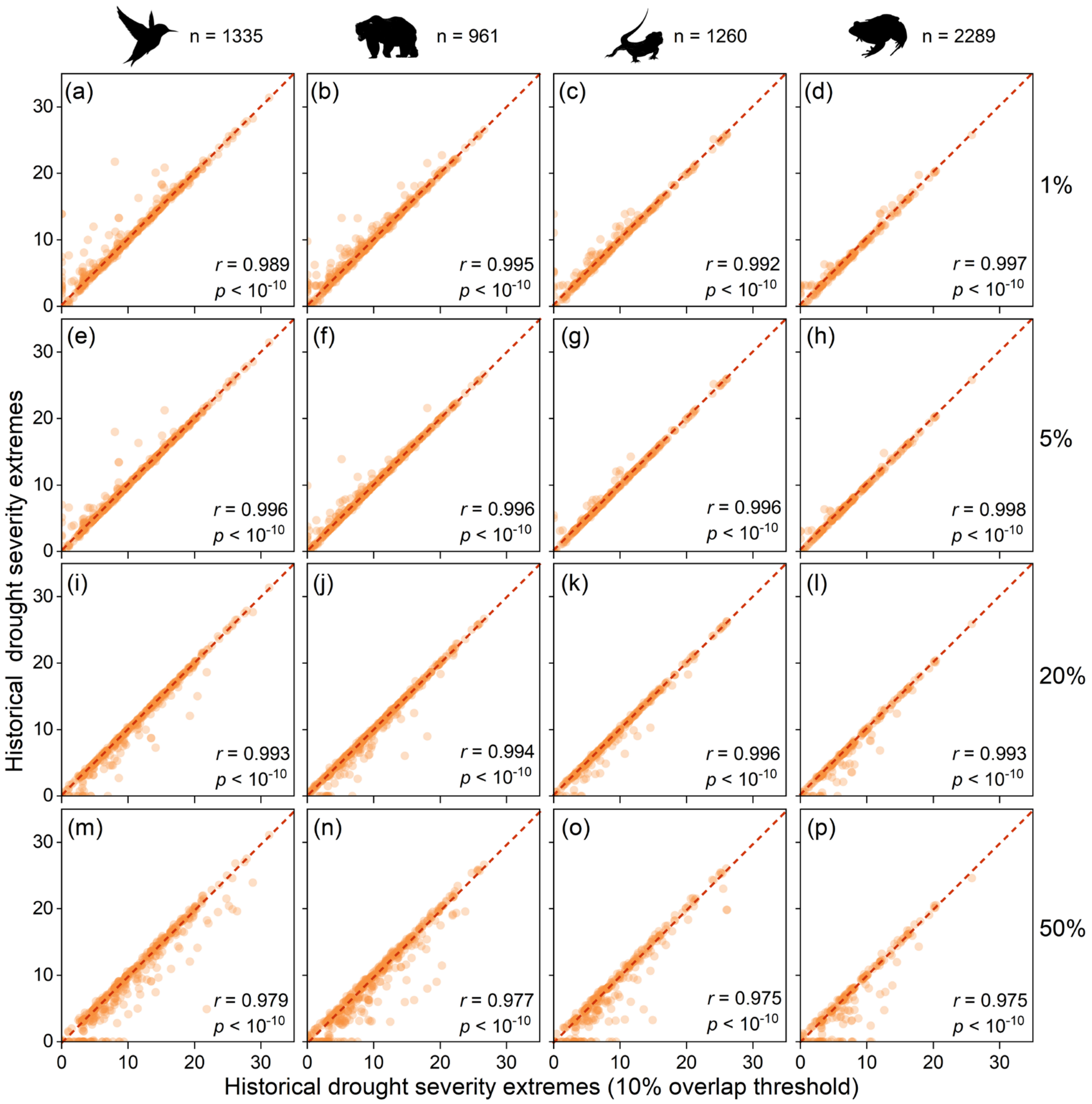


**Supplementary Fig. 18 Sensitivity of species-specific historical drought severity extremes to overlap thresholds for species presence based on threatened terrestrial vertebrate range polygons.** Species-specific historical drought severity extremes were estimated using a 10% overlap threshold compared with different overlap thresholds (1%, 5%, 20%, and 50% in row order). The number n represents the number of threatened species. Red dashed lines indicate the 1:1 line. Pearson's correlation coefficients ($r$) and two-sided unadjusted $p$ values are provided. Source data are provided as a Source Data file.

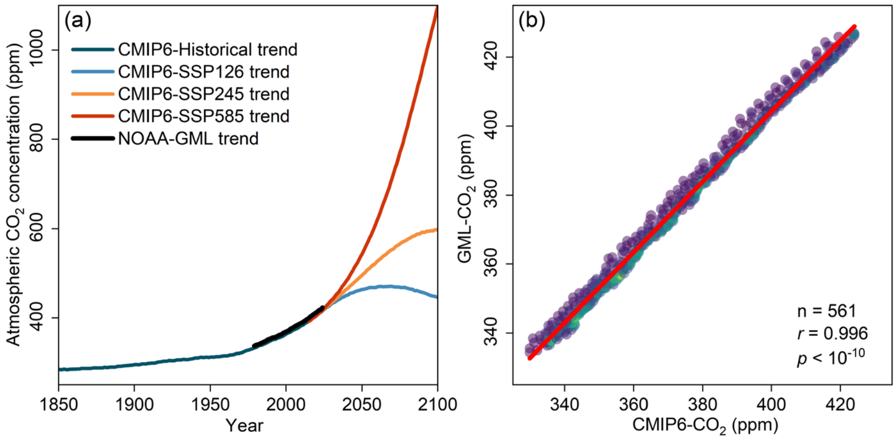


**Supplementary Fig. 19 Trends and validation of the atmospheric $CO_2$ concentrations. a,** Global atmospheric $CO_2$ concentration trends for the GFDL-ESM4 model of CMIP6 (1851 to 2100) and the Global Monitoring Laboratory (GML) of the National Oceanic and Atmospheric Administration (NOAA) (1979 to 2024, https://gml.noaa.gov/ccgg/trends/gl_data.html). **b,** Validation of the atmospheric $CO_2$ concentration data from the GFDL-ESM4 model for the historical period (1979 to 2024) using observational data provided by the NOAA-GML. Red lines represent linear regressions. The number of *n* represents the number of data entries. Pearson's correlation coefficients (*r*) and two-sided unadjusted *p* values are provided. Source data are provided as a Source Data file.

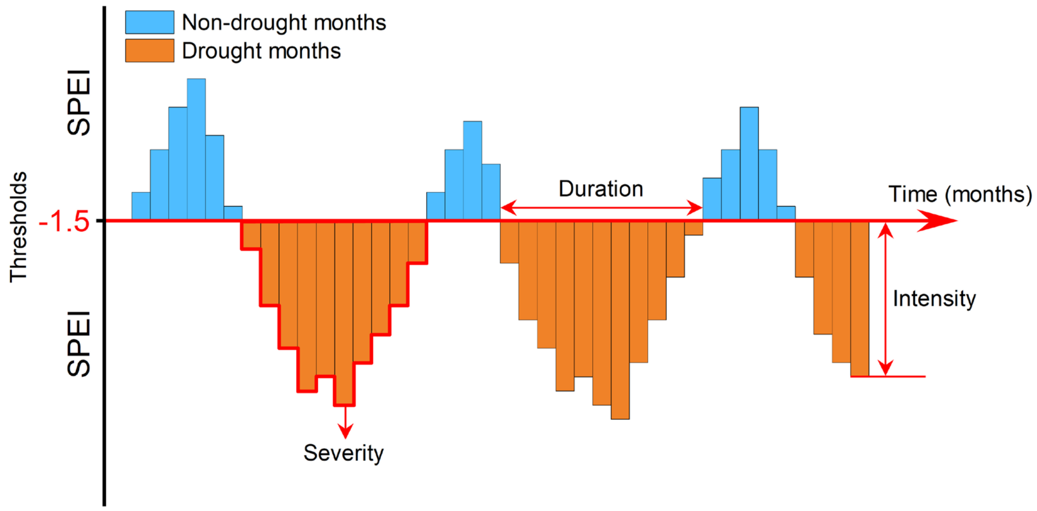


**Supplementary Fig. 20 Methods for defining the characteristics of drought events.** Drought event is defined as an occurrence with the SPEI value at least two consecutive months below the drought threshold (SPEI < –1.5). Drought frequency is defined as the number of non-consecutive drought events occurring in a given time period; drought duration is defined as the consecutive number of months for each drought event; drought intensity is defined as the absolute value of the SPEI value associated with drought months.

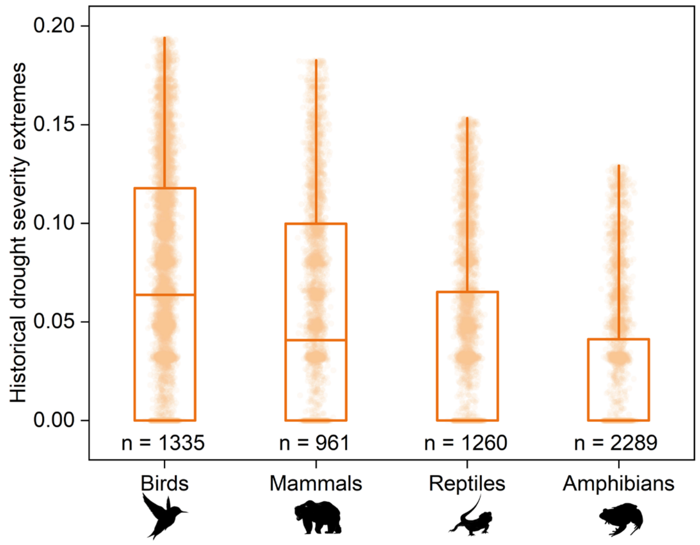


**Supplementary Fig. 21 Species-specific historical drought severity extremes for terrestrial vertebrates.** The number n represents the number of threatened species. The box plot shows the median and the 25th and 75th percentiles, with whiskers representing the 5th and 95th percentiles. Source data are provided as a Source Data file.

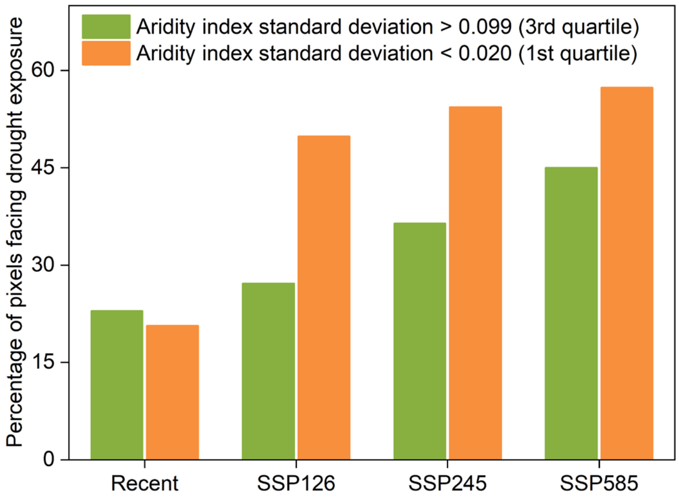


**Supplementary Fig. 22 Drought exposure of threatened terrestrial vertebrate habitats in 0.5° grid cells with low or high spatial climate heterogeneity.** Spatial climate heterogeneity was calculated as the standard deviation of the 1-km resolution aridity index within each grid cell, reflecting regional long-term climate characteristics. Aridity index standard deviation below the 1st quartile indicates lower spatial climate heterogeneity, and above the 3rd quartile indicates higher spatial climate heterogeneity. Source data are provided as a Source Data file.

## Supplementary Tables

**Supplementary Table 1. Countries or regions involved in BHs.**

| Biodiversity hotspot | Countries or regions |
|---|---|
| Atlantic Forest | Brazil, Paraguay, Argentina, Uruguay |
| California Floristic Province | United States of America, Mexico |
| Cape Floristic Region | South Africa |
| Caribbean Islands | Antigua and Barbuda, Bahamas, Dominican, Haiti, Jamaica, Saint Lucia, Saint Vincent and the Grenadines |
| Caucasus | Armenia, Azerbaijan, Georgia, Russia, Iran, Turkey |
| Cerrado | Brazil |
| Chilean Winter Rainfall and Valdivian Forests | Chile, Argentina |
| Costal Forest of Eastern Africa | Somalia, Kenya, Tanzania, Mozambique |
| East Melanesian Islands | Papua New Guinea, Solomon Islands, Vanuatu |
| Eastern Afromontane | Burundi, Democratic Republic of the Congo, Eritrea, Ethiopia, Kenya, Malawi, Mozambique, Rwanda, South Sudan, Tanzania, Uganda, Yemen, Zambia, Zimbabwe |
| Forests of East Australia | Australia |
| Guinean Forests of West Africa | Benin, Cameroon, Cote d'Ivoire, Equatorial Guinea, Ghana, Guinea, Liberia, Nigeria, Sao Tome and Principe, Sierra Leone, Togo |
| Himalaya | Burma, Pakistan, Nepal, Bhutan, India, China |
| Horn of Africa | Somalia, Djibouti, Ethiopia, Eritrea, Kenya, Yemen, Oman, Sudan, Saudi Arabia |
| Indo-Burma | Cambodia, China, Lao PDR, Myanmar, Thailand, Vietnam |
| Irano-Anatolian | Turkey, Georgia, Azerbaijan, Armenia, Iraq, Iran, Turkmenistan |
| Japan | Japan |
| Madagascar and the Indian Ocean Islands | Comoros, Madagascar, Mauritius, Seychelles |
| Madrean Pine-Oak Woodlands | Mexico, United States of America |
| Maputaland-Pondoland–Albany | South Africa, Swaziland, Mozambique |

| | |
|---|---|
| Mediterranean Basin | Albania, Algeria, Bosnia and Herzegovina, Cabo Verde, Egypt, Jordan, Lebanon, Libya, Montenegro, Morocco, North Macedonia, Palestine, Tunisia |
| Mesoamerica | Guatemala, Belize, El Salvador, Honduras, Nicaragua, Costa Rica, Mexico |
| Mountains of Central Asia | Afghanistan, China, Kazakhstan, Kyrgyz Republic, Tajikistan, Turkmenistan, Uzbekistan |
| Mountains of Southwest China | China |
| New Caledonia | New Caledonia |
| New Zealand | New Zealand |
| North American Coastal Plain | Mexico, United States of America |
| Philippines | Philippines |
| Polynesia–Micronesia | Fiji, Micronesia, Polynesia, etc. |
| Southwest Australia | Australia |
| Succulent Karoo | South Africa, Namibia |
| Sundaland | Thailand, Malaysia, Singapore, Brunei, Indonesia, Indian |
| Tropical Andes | Bolivia, Colombia, Ecuador, Peru |
| Tumbes-Choco-Magdalena | Panama, Colombia, Ecuador, Peru |
| Wallaceaea | Indonesia |
| Western Ghats and Sri Lanka | India, Sri Lanka |

**Supplementary Table 2. Richness of species and threatened species of year-round resident terrestrial vertebrates in BHs.**

| Biodiversity hotspot | Birds | | Mammals | | Reptiles | | Amphibians | |
|---|---|---|---|---|---|---|---|---|
| | Species | Threatened | Species | Threatened | Species | Threatened | Species | Threatened |
| Atlantic Forest | 1053 | 161 | 354 | 47 | 409 | 49 | 712 | 130 |
| California Floristic Province | 408 | 42 | 159 | 7 | 78 | 4 | 70 | 18 |
| Cape Floristic Region | 374 | 51 | 116 | 8 | 152 | 4 | 57 | 8 |
| Caribbean Islands | 423 | 52 | 66 | 18 | 381 | 142 | 177 | 133 |
| Caucasus | 323 | 29 | 168 | 12 | 114 | 16 | 23 | 2 |
| Cerrado | 1096 | 139 | 391 | 44 | 459 | 31 | 404 | 30 |
| Chilean Winter Rainfall and Valdivian Forests | 268 | 25 | 82 | 10 | 84 | 17 | 49 | 26 |
| Costal Forest of Eastern Africa | 734 | 74 | 273 | 18 | 270 | 16 | 114 | 25 |
| East Melanesian Islands | 353 | 71 | 88 | 25 | 93 | 2 | 53 | 8 |
| Eastern Afromontane | 1501 | 161 | 650 | 88 | 588 | 45 | 329 | 67 |
| Forests of East Australia | 497 | 52 | 131 | 18 | 279 | 10 | 122 | 27 |
| Guinean Forests of West Africa | 837 | 86 | 384 | 69 | 282 | 24 | 274 | 74 |
| Himalaya | 1101 | 107 | 328 | 40 | 284 | 34 | 180 | 21 |
| Horn of Africa | 856 | 79 | 289 | 24 | 341 | 5 | 67 | 3 |
| Indo-Burma | 1336 | 139 | 493 | 93 | 806 | 133 | 585 | 152 |
| Irano-Anatolian | 385 | 32 | 183 | 11 | 202 | 16 | 29 | 6 |
| Japan | 284 | 35 | 84 | 10 | 40 | 3 | 75 | 35 |
| Madagascar and the Indian Ocean Islands | 302 | 51 | 227 | 126 | 399 | 140 | 305 | 140 |
| Madrean pine-Oak Woodlands | 819 | 78 | 363 | 48 | 502 | 45 | 264 | 124 |
| Maputaland-Pondoland–Albany | 607 | 72 | 199 | 15 | 229 | 11 | 77 | 7 |
| Mediterranean Basin | 560 | 59 | 261 | 33 | 299 | 41 | 110 | 33 |
| Mesoamerica | 1351 | 149 | 467 | 61 | 800 | 120 | 685 | 355 |
| Mountains of Central Asia | 337 | 25 | 151 | 8 | 84 | 6 | 8 | 2 |
| Mountains of Southwest China | 725 | 62 | 285 | 30 | 168 | 7 | 131 | 33 |
| New Caledonia | 111 | 17 | 11 | 5 | 72 | 35 | 1 | 0 |
| New Zealand | 195 | 47 | 6 | 2 | 62 | 42 | 7 | 3 |

| | | | | | | | | |
|---|---|---|---|---|---|---|---|---|
| North American Coastal Plain | 518 | 41 | 166 | 12 | 155 | 10 | 136 | 10 |
| Philippines | 499 | 72 | 185 | 26 | 301 | 25 | 104 | 24 |
| Polynesia–Micronesia | 237 | 47 | 10 | 4 | 32 | 13 | 7 | 0 |
| Southwest Australia | 297 | 29 | 59 | 8 | 200 | 5 | 35 | 3 |
| Succulent Karoo | 324 | 46 | 114 | 5 | 166 | 3 | 32 | 0 |
| Sundaland | 779 | 103 | 414 | 115 | 508 | 51 | 332 | 48 |
| Tropical Andes | 2472 | 329 | 729 | 83 | 922 | 114 | 1479 | 662 |
| Tumbes-Choco-Magdalena | 1275 | 161 | 348 | 35 | 335 | 40 | 310 | 101 |
| Wallaceaea | 654 | 96 | 249 | 58 | 217 | 31 | 53 | 13 |
| Western Ghats and Sri Lanka | 473 | 54 | 143 | 39 | 373 | 143 | 327 | 178 |
