# Supplementary material for: Limiting future warming reduces drought exposure for terrestrial vertebrates
Source: Nat Commun. 2026 May 14;17:6443. doi: 10.1038/s41467-026-73229-3 (PMC13376502; doi:10.1038/s41467-026-73229-3)
Supplement: Supplementary file 2 — Reporting Summary [file 41467_2026_73229_MOESM2_ESM.pdf]

nature portfolio

Corresponding author(s): Jian Sun

Last updated by author(s): April 30, 2026

Reporting Summary

Nature Portfolio wishes to improve the reproducibility of the work that we publish. This form provides structure for consistency and transparency in reporting. For further information on Nature Portfolio policies, see our Editorial Policies and the Editorial Policy Checklist.

Statistics

For all statistical analyses, confirm that the following items are present in the figure legend, table legend, main text, or Methods section.

n/a	Confirmed	
☐	☒	The exact sample size (n) for each experimental group/condition, given as a discrete number and unit of measurement
☒	☐	A statement on whether measurements were taken from distinct samples or whether the same sample was measured repeatedly
☐	☒	The statistical test(s) used AND whether they are one- or two-sided<br>*Only common tests should be described solely by name; describe more complex techniques in the Methods section.*
☒	☐	A description of all covariates tested
☒	☐	A description of any assumptions or corrections, such as tests of normality and adjustment for multiple comparisons
☐	☒	A full description of the statistical parameters including central tendency (e.g. means) or other basic estimates (e.g. regression coefficient) AND variation (e.g. standard deviation) or associated estimates of uncertainty (e.g. confidence intervals)
☐	☒	For null hypothesis testing, the test statistic (e.g. F, t, r) with confidence intervals, effect sizes, degrees of freedom and P value noted<br>*Give P values as exact values whenever suitable.*
☒	☐	For Bayesian analysis, information on the choice of priors and Markov chain Monte Carlo settings
☒	☐	For hierarchical and complex designs, identification of the appropriate level for tests and full reporting of outcomes
☐	☒	Estimates of effect sizes (e.g. Cohen's d, Pearson's r), indicating how they were calculated

Our web collection on statistics for biologists contains articles on many of the points above.

Software and code

Policy information about availability of computer code

Data collection	No software or code was used to collect data.
Data analysis	Data analysis was performed in Python version 3.8. The codes used in this study are available at the Institute of Tibetan Plateau Research, Chinese Academy of Sciences (https://doi.org/10.11888/Terre.tpdc.302324).<br>Firstly, S1_Calculate_PET_PM_and_PET_CO2.py, S2_Combine_PET_PM_and_PET_CO2.py, and S3_Calculate_SPEI were used to calculate SPEI time series for different GCMs from CMIP6. Secondly, S4_Calculate_Drought_Characteristics.py was employed to quantify drought severity during historical, recent, and future periods. Thirdly, S5_Gridded_Species_Geographic_Range_Polygons.py was used to grid the species' geographic range polygons. Fourthly, S6_Calculate_Exposure_Range.py was applied to assess the exposure of species' geographic ranges to drought severity exceeding their historical extremes.<br>Subsequent data analysis and plotting were processed with Origin 2022, ESRI ArcGIS Pro version 3.0, and Adobe Illustrator 2025.

For manuscripts utilizing custom algorithms or software that are central to the research but not yet described in published literature, software must be made available to editors and reviewers. We strongly encourage code deposition in a community repository (e.g. GitHub). See the Nature Portfolio guidelines for submitting code & software for further information.

Data

Policy information about availability of data

All manuscripts must include a data availability statement. This statement should provide the following information, where applicable:

- Accession codes, unique identifiers, or web links for publicly available datasets
- A description of any restrictions on data availability
- For clinical datasets or third party data, please ensure that the statement adheres to our policy

The historical, recent, and future relative drought severity data generated in this study have been deposited in the Institute of Tibetan Plateau Research, Chinese Academy of Sciences, with open access (https://doi.org/10.11888/Terre.tpdc.302324). Geographic range data for biodiversity hotspots is available at https://doi.org/10.5281/zenodo.3261807. CMIP6 climate data is available at https://metagrid.esgf-west.org/search/cmip6/. Geographic distribution data for birds is available at https://datazone.birdlife.org/contact-us/request-our-data (BirdLife v.8.0), and for mammals, reptiles, and amphibians at https://www.iucnredlist.org/resources/spatial-data-download (IUCN v.6.3). Map for drylands is available at https://data-gis.unep-wcmc.org/portal/home/item.html?id=789fcac8959943ab9ed7a225e5316f08. Protected area coverage data is available at www.protectedplanet.net and www.openstreetmap.org/. Human footprint data in 2020 is available at https://doi.org/10.5061/dryad.ttdz08m1f.

Research involving human participants, their data, or biological material

Policy information about studies with human participants or human data. See also policy information about sex, gender (identity/presentation), and sexual orientation and race, ethnicity and racism.

Reporting on sex and gender	NA
Reporting on race, ethnicity, or other socially relevant groupings	NA
Population characteristics	NA
Recruitment	NA
Ethics oversight	NA

Note that full information on the approval of the study protocol must also be provided in the manuscript.

Field-specific reporting

Please select the one below that is the best fit for your research. If you are not sure, read the appropriate sections before making your selection.

☐ Life sciences ☐ Behavioural & social sciences ☒ Ecological, evolutionary & environmental sciences

For a reference copy of the document with all sections, see nature.com/documents/nr-reporting-summary-flat.pdf

Ecological, evolutionary & environmental sciences study design

All studies must disclose on these points even when the disclosure is negative.

Study description	This study quantitatively assesses the exposure of terrestrial vertebrates in global biodiversity hotspots to recent and future drought severity exceeding their historical extremes, further revealing conservation gaps and governance challenges in drought exposure to inform decisions for pre-emptive conservation actions.
Research sample	Geographic range data for terrestrial vertebrates associated with 36 global biodiversity hotspots and climate data from 1851 to 2100.
Sampling strategy	NA
Data collection	The historical, recent, and future relative drought severity data generated in this study have been deposited in the Institute of Tibetan Plateau Research, Chinese Academy of Sciences, with open access (https://doi.org/10.11888/Terre.tpdc.302324). Geographic range data for biodiversity hotspots is available at https://doi.org/10.5281/zenodo.3261807. CMIP6 climate data is available at https://metagrid.esgf-west.org/search/cmip6/. Geographic distribution data for birds is available at https://datazone.birdlife.org/contact-us/request-our-data (BirdLife v.8.0), and for mammals, reptiles, and amphibians at https://www.iucnredlist.org/resources/spatial-data-download (IUCN v.6.3). Map for drylands is available at https://data-gis.unep-wcmc.org/portal/home/item.html?id=789fcac8959943ab9ed7a225e5316f08. Protected area coverage data is available at www.protectedplanet.net and www.openstreetmap.org/. Human footprint data in 2020 is available at https://doi.org/10.5061/dryad.ttdz08m1f.
Timing and spatial scale	Timing scale: Based on climate data from 1851 to 2100, the drought severity was quantified for the historical (1851-1900), recent (1975-2024), and future (2051-2100) periods. We collected species geographical range data in April 2024 and protected areas data in December 2024. The statuses of national development are for 2022, and the population and human footprint data are for 2020.<br>Spatial scale: Global and biodiversity hotspots.
Data exclusions	This study excluded vertebrates in both marine and terrestrial habitats and used only breeding, extant, and native species ranges.

	Additionally, point data layers of protected areas, protected areas without designated, inscribed, or established status, and the Other Effective Conservation Measures (OECMs) are excluded from our analysis.
Reproducibility	All attempts to repeat the analysis with these data collected and statistical methods would be successful.
Randomization	Randomization was not relevant to our study as our study was not experimental.
Blinding	Not relevant to this study.

Did the study involve field work? ☐ Yes ☒ No

Reporting for specific materials, systems and methods

We require information from authors about some types of materials, experimental systems and methods used in many studies. Here, indicate whether each material, system or method listed is relevant to your study. If you are not sure if a list item applies to your research, read the appropriate section before selecting a response.

Materials & experimental systems

n/a	Involved in the study
☒	☐ Antibodies
☒	☐ Eukaryotic cell lines
☒	☐ Palaeontology and archaeology
☒	☐ Animals and other organisms
☒	☐ Clinical data
☒	☐ Dual use research of concern
☒	☐ Plants

Methods

n/a	Involved in the study
☒	☐ ChIP-seq
☒	☐ Flow cytometry
☒	☐ MRI-based neuroimaging

Plants

Seed stocks	NA
Novel plant genotypes	NA
Authentication	NA
