# Supplementary material for: Limiting future warming reduces drought exposure for terrestrial vertebrates
Source: Nat Commun. 2026 May 14;17:6443. doi: 10.1038/s41467-026-73229-3 (PMC13376502; doi:10.1038/s41467-026-73229-3)
Supplement: Supplementary file 3 — Transparent Peer Review file [file 41467_2026_73229_MOESM3_ESM.pdf]

nature portfolio

Peer Review File

Limiting future warming reduces drought exposure for terrestrial vertebrates

Corresponding Author: Professor Jian Sun

This file contains all reviewer reports in order by version, followed by all author rebuttals in order by version.

Attachments originally included by the reviewers as part of their assessment can be found at the end of this file.

Version 0:

Reviewer comments:

Reviewer #1

(Remarks to the Author)
Dear authors,
thanks for the interesting study but there are quite some flaws that need to be addressed and I also think you are overselling your results in terms of what the sustainability scenario really means in your analysis (it is low climate change you cover, not the rest). See detailed comments in the pdf.
I have also provided some references of quite similar studies to yours.
I hope my comments help to improve the manuscript.
bests
christopher reyer

(Remarks on code availability)

Reviewer #2

(Remarks to the Author)
This manuscript presents an ambitious and valuable global assessment of projected changes in drought severity across biodiversity hotspots and their implications for terrestrial vertebrates. The integration of multiple SSP scenarios and the attempt to link drought dynamics with biodiversity and socio-economic dimensions represent important contributions to understanding climate–biodiversity interactions. Further, the figures are very informative. While the study complements recent research, with a distinct method of drought quantification and a broader framing, several aspects require substantial revision before the manuscript is suitable for publication.
The manuscript would benefit from clearer and more concise writing — the text is often repetitive and overloaded with adjectives, which obscures the main messages. Substantively, I have two major concerns. First, while inclusion of three SSPs is commendable, the use of SSP585 as the leading scenario warrants reconsideration given the growing consensus that it represents an increasingly implausible high-emission pathway. Second, the drought threshold adopted (SPEI < –0.5) is extremely low; such values indicate only mildly dry or near-normal conditions and may not meaningfully reflect ecological drought stress. Using this as a starting point for quantifying drought frequency and severity likely inflates drought intensity estimates and, consequently, the extent of areas and species deemed exposed to “extreme drought.”

-Title:
I would avoid the use of 'significantly' in a non-statistical context. Perhaps 'Representative Concentration PathwayS determine future drought exposure for terrestrial vertebrates'.

-Abstract:
L30: exposure to drought frequency, intensity, or both?
L31: meaning of 'beyond historical records' is not entirely clear here. Droughts more extreme than in the historical records, or droughts predicted in the future?
L40: conservation gaps? As in, conservation issues?

L41: social equity appears a bit out of the blue and an afterthought, if there is space, there should be a line on how conservation money is not allocated according to spatial patterns of biodiversity.

-Introduction:
General: the writing of the introduction, results, and discussion stands to be improved. Firstly, there are phrasing and sentence structure issues. Further, the writing could be more concise. I provide examples for the first paragraph of the introduction:

1-The first sentence is 50 words with many nested clauses, making it difficult to follow.
2-'skepticism is ongoing' (awkward phrasing)
3-'Despite their immense value' (either omit this or expand upon it, it's just awkwardly postulated here)
4- 'Increased future extreme drought will doubtless pose a considerable threat to species in critical but fragile BHs' (overabundance of adjectives, almost half of the word count)
5-'Recurring extreme drought will potentially have unexpected negative cascading effects on ecosystems' (half of the words are adjectives)
6- L58–60 and 60–62 essentially say the same thing
7- 'The planet's exceptionally species rich....' sounds awkward because of the unnecessary adjective. The planet is not exceptionally rich in species; it's just the only planet with a known biological diversity.

L66–68: there is no clear link between the first statement (inequitable conservation spending) and the second (drought as a compounding threat to biodiversity).
L71: informal use of unfortunately (and another adjective).
L64–78: The overarching message (social equity issues exacerbate threats to BHs already posed by climatic change and habitat loss) is not immediately clear because the paragraph subject seems to change throughout (e.g. drought is mentioned in the first sentence, then we switch to habitat loss, go back to drought, and in between we mention issues of social equity). A more logical approach could be;
1- habitat loss and fragmentation considered as primary threats
2- climatic change, one of its facets being extreme drought, interacting and compounding with these threats
3- how this is all further problematized by social equity issues.

L82: again, careful proofreading and rewriting necessary: 'such studies have not yet focused on…', remove 'yet' as these studies are already published and cannot be amended.
L82–85: I believe this overstates the novelty of the study, because what you state to be novel has largely been done here - https://onlinelibrary.wiley.com/doi/full/10.1111/gcb.70272.
Your study still has novelty, and is complementary, because it
1- focusses largely on vertebrates with an already threatened status (as opposed to e.g. Least Concern species that nevertheless have 'Drought' assigned as a conservation threat by IUCN)
2- looks at drought exposure differently
3- zooms in on the governance challenges posed by the results
The paper linked above is a good opportunity to acknowledge recent findings on this topic and further contextualize your study.
L87: again, not sure what 'beyond the historical record' means, though I think you mean they surpass historical drought exposure.

-Results:

General: again, I believe there are many opportunities to tighten the writing:
unprecedented future expansion of drought risk = increased drought risk
the risk of future drought = future drought risk
to visually indicate = to visualize
the degree of exposure to extreme drought = extreme drought exposure
after we aggregated all future climate scenarios = after aggregating climate scenarios, etc.
L106: do you mean compared to the global average?
L115–118: I would lead all findings by reporting results under the intermediate SSP, followed by the extremes (as some sort of 'confidence interval'). One reason is that SSP 585 criticized for not being very realistic (https://doi.org/10.1038/s41467-024-52437-9). It projects virtually no GGE mitigations even though such initiatives have been underway globally (though, of course, not in desirable degrees).
L121–122: I'm not sure the IPCC report states this anywhere?
see https://www.congress.gov/crs-product/R47082?utm=, paragraph below Table 1
L130: it is currently unclear in this paragraph whether you also quantified exposure in these two ways.
L135–136: more careful phrasing is needed here. A threatened conservation status does not imply higher drought vulnerability/sensitivity (see van den Bosch et al., 2025). I would also not specify 'vulnerable to extinction', as there are many things that precede extinction (distribution shifts, local extirpations, abundance declines, etc.).
L140–143: to meaningfully interpret these numbers, you may want to compare it to a recent period.
L140–146: I got confused: for SSP585 you report mean range percentage exposed and percentage of species for which > 50% of the range is exposed, but for the other SSPs you appear to report different statistics? Again the mean range percentage but also the percentage of species exposed?
L144: number missing in 'SSP 24x'
L138–155: see previous comment about the order of SSPs in reporting results.

L157: I’m unsure what ‘clustering’ means in this regard.
L166–171: just making sure we are basing this on proportions per species class, so adjusting for the fact some species will be richer in birds than mammals etc.?
L179: the heading for this section ‘benefits from sustainable development’ strikes me as odd, I would explicitly link back to the SSPs.
L184–192: the phrasing of ‘reducing’ and ‘avoiding’ exposure confuses me. What is the difference between them?
L206: ok, but while protected status might prevent habitat loss, amongst other things, it does not immediately prevent climatic change in that area. Nor would granting protected status to BHs necessarily limit their future drought exposure. So this would need to be discussed a bit more in-depth.
L208: what are local versus global BHs? These terms have not been clarified.
L215–216: it should be mentioned the SSPs also project changing human populations and, by proxy, human footprints, which could not be accounted for with the static human footprint index.
L217–221: the content is good, but it’s not really fleshed out. It’s not telling me how e.g. habitat loss or fencing things can exacerbate drought effects, and the point about human rights issues also remains quite vague.

Discussion:
L229: what’s the difference between ‘threaten survival of species’ and ‘exacerbate extinction risk’?
L234: again, I don’t think the IPCC report insinuates SSP585 resembles the current trajectory, and I highly recommend looking into considering SSP245 as your leading ‘middle-of-the-road’ scenario.
L234–236: there again seems to be some repetition. I can’t see the difference between these lines and lines L236–238.
L260: choose symbiosis or mutualism.
L270–295: a crucial point that is missing is that while you quantify drought exposure, we urgently need science on drought vulnerability, to know which species are most likely to decline under this increased drought exposure. Species-specific drought vulnerability is the missing link between estimating their future exposure and knowing how this will affect them, which can facilitate conservation prioritization. Currently, drought vulnerability of most vertebrates is not well known (van den Bosch et al., 2025).
L301: that is under the assumption profitable agriculture and forestry will actually benefit the local population…
L297–322: I appreciate the integration of social issues, but I think we’re bringing in too many new terms here that are not fully explained and, to an extent, are only indirectly linked to your results (e.g. ‘opportunity costs’, ‘IPLC-based conservation’, ‘differentiated responsibilities’, etc.
L331: this goes both ways, some species may have good buffers, others may be more vulnerable than we currently know. I’d generalize this to say the missing link is learning more about species-specific drought vulnerability.
L324: perhaps to include in this ‘limitations’ paragraph is the fact that you had to use an initial drought threshold for your calculations (currently SPEI-12 of -0.5). If many past months had -0.49 and many of the projections have -0.51, that will greatly increase drought frequency (part of your severity calculations) despite there being little actual change. This is difficult to avoid, even if you change the threshold (which I think is necessary).
L360–364: this is another part that needs extensive rewriting, it is extremely difficult to follow.
Methods:
L482–493: I’m not sure this level of detail is needed here, just a reference may be sufficient.
L498–509: same as above, I think the concept of SPEI can be explained in simpler terms.
L524: I have an issue with the drought threshold used here. The lower threshold could be described as mildly dry, or even near-normal. This is problematic, because due to the calculation of drought severity, a large increase in the number of months with slightly drier conditions that are still not considered moderate, severe, or extreme drought (per the SPEI index), could lead to the quantification of ‘extreme drought’ (while in SPEI-terms, ‘extreme drought’ would be limited to numbers below SPEI-12 = -2).
Firstly, I think it is necessary to rerun the analysis with a more common drought threshold (e.g. SPEI-12 = -1.5), because you are inferring ‘extreme drought’ for high percentages of species’ ranges, while in many cases it may in fact be long-term gradual shifts from ‘dryish’ to ‘quite dry’. With a stronger threshold there will be fewer events to consider but they will be more likely to have biological impacts, and so the change in drought you calculate will be more likely to have the grave impacts you postulate.
Secondly, I would then rephrase ‘extreme drought’ to ‘extreme changes in drought severity’, to avoid confusion, given your findings of changing drought patterns may or may not pertain to severe/extreme droughts in SPEI-12 terms.
L627: calculating
Fig 1: I’m not sure how helpful the tan outlines are, they confused me until I realized they’re likely meant to delineate the hotspots?
Fig 3: again a very informative figure, though I have a hard time discerning which line refers to which SSP. Perhaps it would be possible to use three more contrasting colors, but then use two different patterns (solid / striped, for example). It may also confuse some readers that a striped vertical line is used for different purposes in 3a-e versus 3f.
Fig. 4: small mistake in the annotations, both mention BHs with lower species drought crisis
Fig. 5: I’m unsure about this figure: while visually appealing and conceptually interesting, this figure appears to extend substantially into social science and conservation policy, which is not directly part of the analysist. A lot of themes are introduced that, while important, feel somewhat disconnected from the empirical results presented earlier. There is enough material here to warrant separate studies, and its inclusion risks diluting the paper’s focus on drought and biodiversity. I should note that I am less familiar with these topics, and will therefore leave it to the editor and other reviewers to judge whether the inclusion of this figure strengthens the manuscript or detracts from its focus.

(Remarks on code availability)
I do not foresee to have the time necessary to review the code.

Version 1:

Reviewer comments:

Reviewer #1

(Remarks to the Author)
thanks for the very deep and clear revisions.
i have only made very few minor comments and fixed typos etc. in the attached ms.
all the best
christopher reyer

(Remarks on code availability)

Reviewer #2

(Remarks to the Author)
Please find my review report attached.

(Remarks on code availability)

Version 2:

Reviewer comments:

Reviewer #2

(Remarks to the Author)
The authors have conducted extensive re-analysis to address the concerns that were raised, while the writing of the manuscript has also been greatly improved.

I believe the manuscript is ready for publication, and would like to congratulate the authors on a great research contribution.

(Remarks on code availability)

Open Access This Peer Review File is licensed under a Creative Commons Attribution 4.0 International License, which permits use, sharing, adaptation, distribution and reproduction in any medium or format, as long as you give appropriate

credit to the original author(s) and the source, provide a link to the Creative Commons license, and indicate if changes were made.
In cases where reviewers are anonymous, credit should be given to 'Anonymous Referee' and the source.
The images or other third party material in this Peer Review File are included in the article’s Creative Commons license, unless indicated otherwise in a credit line to the material. If material is not included in the article’s Creative Commons license and your intended use is not permitted by statutory regulation or exceeds the permitted use, you will need to obtain permission directly from the copyright holder.
To view a copy of this license, visit https://creativecommons.org/licenses/by/4.0/

Reviewer #1 (Remarks to the Author):

Dear authors,

thanks for the interesting study but there are quite some flaws that need to be addressed and I also think you are overselling your results in terms of what the sustainability scenario really means in your analysis (it is low climate change you cover, not the rest). See detailed comments in the pdf.

I have also provided some references of quite similar studies to yours.

I hope my comments help to improve the manuscript.

bests

christopher reyer

Response: Dear Dr. Reyer, we thank you very much for your thoughtful and insightful comments on our work. We have made substantial revisions to our manuscript according to your suggestions. The specific corrections are detailed below. Please do not hesitate to contact us if further material or information is needed.

Title:

Comment #1: I think the title is very misleading since you are referring to SSP126 but only as far as climate is concerned. there is no "sustainable land-use" or anything considered in your analysis. you simply assume that the BHs will remain where they are not being affected by land-use or human development decisions. While the IAMs/LU models behind the Land-use pattern used in the SSPs, respect existing conservation areas to some extent, there is really no further consideration of the many aspects of sustainability in your analysis. And earlier studies including land-use change have shown that there are high risks for biodiversity from land use in the sustainability scenario.

Yours is a climate impact study and the other dimension of SSP126 that actually lead to the "sustainable climate pathway" are ignored.

Therefore, I think the title is misleading and sth like "Low warming reduces..." would be more appropriate. it is then, however, also clear that your findings are not that new.

Response: We are grateful for your clarification on sustainable development pathways. We acknowledge that our analysis mainly focused on the contribution of SSP126 to mitigating drought exposure in the climate dimension, without explicitly accounting for the impacts of land-use changes or other human development decisions under sustainable scenarios. Therefore, we agree that the original title is misleading since it implies an assessment of broader sustainable scenarios.

Following your subsequent constructive comments, we have now refined our analysis to focus on assessing drought exposure exceeding species' historical extremes (see response to comment

#12). Accordingly, we have revised the title to "**Limiting future warming reduces drought exposure for terrestrial vertebrates**" to better align with our main findings. Additionally, we revised all instances of "sustainable development" throughout the text to "limiting future warming". We also added a discussion on the impacts of land-use change on biodiversity under the SSP126 scenario (see response to comment #19).

Abstract:

Comment #2: Do you mean in a sense of integrating ecological, social/cultural and economic aspects of conservation? or sth more like "enduring"

Response: Thank you for pointing out the ambiguity of the term "sustainable". Here, we mean addressing the threats of intensifying drought severity to biodiversity requires not only ambitious climate strategies to mitigate drought exposure for species, but also adaptive conservation measures to help species adapt to increasing drought exposure without compromising local well-being.

Therefore, we have revised the wording to "adaptive", as follows:

L28–29: *Ambitious and adaptive conservation efforts are needed to curb biodiversity loss, particularly as drought severity intensifies globally.*

Comment #3: "losses" is maybe the more appropriate term?

Response: Thanks. We have amended the wording, as follows:

L28–29: *Ambitious and adaptive conservation efforts are needed to curb biodiversity loss, particularly as drought severity intensifies globally.*

Comment #4: Are those habitats shifting?

Response: Thank you for raising the important issue. We have revised the manuscript to explicitly refer to the **current geographic range** throughout (L32–34), thereby avoiding any implication that future range shifts are directly modeled. This reflects a deliberate scope choice rather than an oversight. Our analysis focuses on species' exposure patterns within their realized current geographic ranges, which are directly relevant for near-term conservation planning and management decisions.

While climate-driven habitat range shifts are increasingly important, explicitly modelling them would require additional assumptions regarding species dispersal capacity, landscape connectivity, and the rate of climate change, introducing substantial uncertainty and model complexity. We therefore treat potential range shifts as a secondary-order process beyond the scope of the current study. Furthermore, we argue that assessing exposure within the species' realized current geographic range provides a crucial conservative baseline. If species cannot

shift habitats (due to fragmentation in BHs), this metric represents their realistic exposure.

Nevertheless, we explicitly acknowledge this limitation and discuss how future range shifts could influence exposure estimates in the *Discussion* (L266–270) and *Methods* (L500–502) section, and we highlight this as a priority direction for future research (L283–285).

L32–34: *We show that 22.6% of threatened terrestrial vertebrates (especially reptiles and amphibians) have already been exposed to drought severity exceeding their historical extremes across at least half of current geographic range in recent decades.*

L266–270: *The fundamental niche limits of species may be wider than their realized niche limits3[8,56]. Some species may buffer drought exposure through behavioral adaptations or phenotypic plasticity, such as utilizing microhabitats[50], shifting seasonal activities[67], adapting new habitats to alter current range[56], or evolving physiological tolerance to drought[13].*

L500–502: *Notably, complex ecological processes, such as species utilizing climate refuges or dispersing into new habitats, may alter exposure estimates[56,57].*

L283–285: *…and explicitly estimate the consequences of drought exposure, including habitat range shifts and impacts on interactions and trophic dynamics across taxa in food networks[7,22,57].*

Comment #5: still not the right word in this context I believe.

Response: We have replaced the term "crisis" with "exposure" to better align with our findings, as follows:

L37–39: *Notably, a twofold difference exists between the exposure estimates in optimistic (SSP126) and pessimistic (SSP585) warming scenarios, highlighting the potential for ambitious climate mitigation to reduce drought exposure.*

Comment #6: grammar. "the species drought crises" does not work here and elsewhere in the manuscript. i think it is too general term.

Response: We agree that the term is too general and imprecise, and have revised the sentence to more clearly articulate our research focus—drought exposure. As follows:

L39–42: *However, given that future drought exposure will increase in most BHs, and exposed regions currently face inadequate protection and substantial social burdens, expanding adaptive conservation actions without compromising local well-being is urgent.*

Introduction:

Comment #7: How are extreme droughts defined given the degree of drought at which ecosystems and species are at risks differs quite a lot across ecosystems and climate zones. See

my comment below about the “10 times higher” definition of drought.

Response: Thank you for this insightful comment. In our refined analysis, we defined species-specific historical drought severity extremes related to drought vulnerability for each species to identify which species and regions are more threatened by intensifying drought (see response to comment #12).

To this end, we removed the description of “extreme drought” and revised the sentence as follows:

L57–59: *Therefore, evaluating the impacts of intensifying drought on biodiversity in critical yet vulnerable BHs is fundamental to guiding pre-emptive conservation efforts*[3,24].

Comment #8: unclear what you mean here. what is the “association of the threat and the burden”?

Response: Thank you for pointing out this issue. We have revised the sentence to emphasize that clarifying the conservation gaps and governance challenges in regions facing drought exposure is important in guiding equitable and adaptive conservation actions. As follows:

L70–73: *Consequently, identifying the species and regions most affected by intensifying drought in BHs, and clarifying their conservation gaps and governance challenges, can inform decisions to develop or modify equitable and adaptive conservation measures*[5,34].

Comment #9: There are least two studies, in well respected journals, that actually do study future climate impacts, including droughts, in BHs:

https://onlinelibrary.wiley.com/doi/full/10.1111/gcb.70272

https://doi.org/10.1111/geb.12228

I think you miss to position your work in the existing literature here.

Response: Thank you for this important comment. We accept that the literature review in the original manuscript omitted these studies. Thus, we have reworked the literature review to acknowledge a wider range of prior research.

Additionally, following your subsequent key comments, we have improved our analysis methodology. Here we reposition our research by clarifying that most previous studies have overlooked species-specific drought vulnerability in assessing the impacts of intensifying drought on biodiversity. To this end, following the recent research framework for temperature extremes (Trisos et al., 2020; Murali et al., 2023), our study applies the “historical extremes” approach specifically to asses exposure of species to drought severity exceeding their niche limits, providing a distinct angle on drought threats.

Trisos, C. H., Merow, C. & Pigot, A. L. The projected timing of abrupt ecological disruption from climate change. Nature 580, 496-501 (2020).

Murali, G., Iwamura, T., Meiri, S. & Roll, U. Future temperature extremes threaten land vertebrates. Nature 615, 461-467 (2023).

The specific revisions are as follows:

L75–85: *Previous studies integrating climate change simulations with species distribution modeling demonstrate that species, both globally and within BHs, are increasingly exposed to droughts and other climate extremes*[13,20,35-40]*. However, most existing assessments quantify these impacts using generalized climate anomalies or fixed historical thresholds*[18,40]*. While such approaches capture intensifying drought conditions, they often overlook the species-specific differences in drought vulnerability, which are critical determinants of survival*[18,40]*. To address the scarcity of physiological data for most species*[40]*, we estimated drought tolerance limits using the historical extremes of species realized climatic niches. We then assessed the exposure of species' geographic ranges to recent and future drought severity exceeding their historical extremes (see Supplementary Fig. 1 and Methods).*

Comment #10: adaptation and mitigation are clearly defined in the climate change literature. but here it seems adaptation and mitigation mean the same? somehow coping with the damage?

Response: Thank you for raising this important issue. We agree that mitigation and adaptation have distinct meanings in the context of climate change, and the original wording conflated them.

In our revised manuscript, we clearly distinguish between mitigation and adaptation for drought exposure, emphasizing the need for ambitious climate mitigation strategies to reduce drought exposure, as well as adaptive conservation measures to cope with increasing drought exposure without compromising local well-being. Here we have revised the sentence:

Revised manuscript

L85–89: *Focusing on threatened terrestrial vertebrates (as identified by the International Union for Conservation of Nature*[41]*) within BHs, this analysis identifies critical conservation gaps and governance challenges in regions facing drought exposure, providing spatially explicit guidance for conservation planning.*

Results:

Comment #11: this is unclear. 2001-2020 is in the 21st century?

and if this is the historical period, you miss a lot of earlier droughts that the ecosystems/species might have endured over the past hundreds of years. I think you need to deduce the pre-industrial (or long-term average drought frequency, duration and intensiy) from a kind of pre-industrial control climate (picontrol runs of the ESM for example).

Response: Thank you for pointing out this error and offering constructive comments. Following this suggestion, we have redefined the historical baseline as the pre-industrial period

(1851–1900), rather than using the 2001–2020 period. We have recalculated drought severity for all periods accordingly and reanalyzed recent and future drought exposure for species based on the new historical baseline.

The following revisions relate to the *Results* (L93–96) and the *Methods* (L451–455):

L93–96: *To characterize drought dynamics in BHs following large-scale anthropogenic warming, we quantified and compared the drought severity for historical (pre-industrial, 1851 to 1900), recent (1975 to 2024), and future (2051 to 2100) periods.*

L451–455: *Based on the median of SPEI time series across GCMs, we calculated the total drought severity for the historical baseline (pre-industrial, 1851 to 1900), recent (1975 to 2024), and future (2051 to 2100) periods using a 50-year timescale, further normalized to relative drought severity, in order to assess drought dynamics since the onset of large-scale anthropogenic warming.*

Comment #12: this seems very arbitrary. any evidence to support this? it is no surprise that your results are supported by the sensitivity analysis, also 5 times higher is still really a lot. and see my comment on the period 2001-2020 not really representing the actual long-term distribution of droughts in any grid cell.

Response: Thank you for raising these important concerns. We completely agreed with this critique. We have abandoned the arbitrarily 10-fold threshold. Instead, we now calculate a species-specific drought severity threshold defined as the 99th percentile of drought severity experienced within that species' realized niche during the pre-industrial baseline. This biologically grounded metric replaces the arbitrary statistical threshold to integrate species-specific drought vulnerability into drought exposure assessments. Details are as follows:

L461–484:

Defining species-specific historical drought severity extremes

The intensification of drought severity will force organisms to endure unprecedented environmental stresses[13,14,17]*, but the exact impacts are contingent upon species-specific drought vulnerability*[18,40]*. However, physiological data on drought vulnerability for most species remain unavailable*[40]*. In fact, a species' geographic distribution results from long-term adaptation to climatic conditions*[56]*, and the climatic extremes experienced across its realized distribution likely represent its climatic niche limits*[38,56,89,90]*. Therefore, we applied methods similar to studies on temperature extremes*[36,38]*, approximating species-specific drought tolerance limits by estimating historical drought severity extremes within species' realized niches. Specifically, species-specific historical drought severity extremes were calculated as the 99th percentile of drought severity experienced across all grid cells within each species' expert-validated global distribution during the historical baseline (Supplementary Figs. 1 and 20). This percentile-based method reduces the effects of extreme outliers and overestimated species ranges*[36,81]*. Importantly, considering recent droughts have already imposed significant impacts on ecosystems*[14-16,21,22]*, we use the pre-industrial period (1851 to 1900, characterized by relatively stable climate conditions) as the historical baseline. It must be underlined that species*

drought tolerance limits estimated through realized niches may be conservative, as species may not have reached their actual physiological limits in the pre-industrial period. Furthermore, some species' range maps may underestimate the extent of their climatic niche due to limited long-term historical records for calibration[80]. Phenotypic plasticity of species to drought stress and species interactions may lead to misjudgments of drought tolerance limits by applying historical drought severity extremes[13,57].

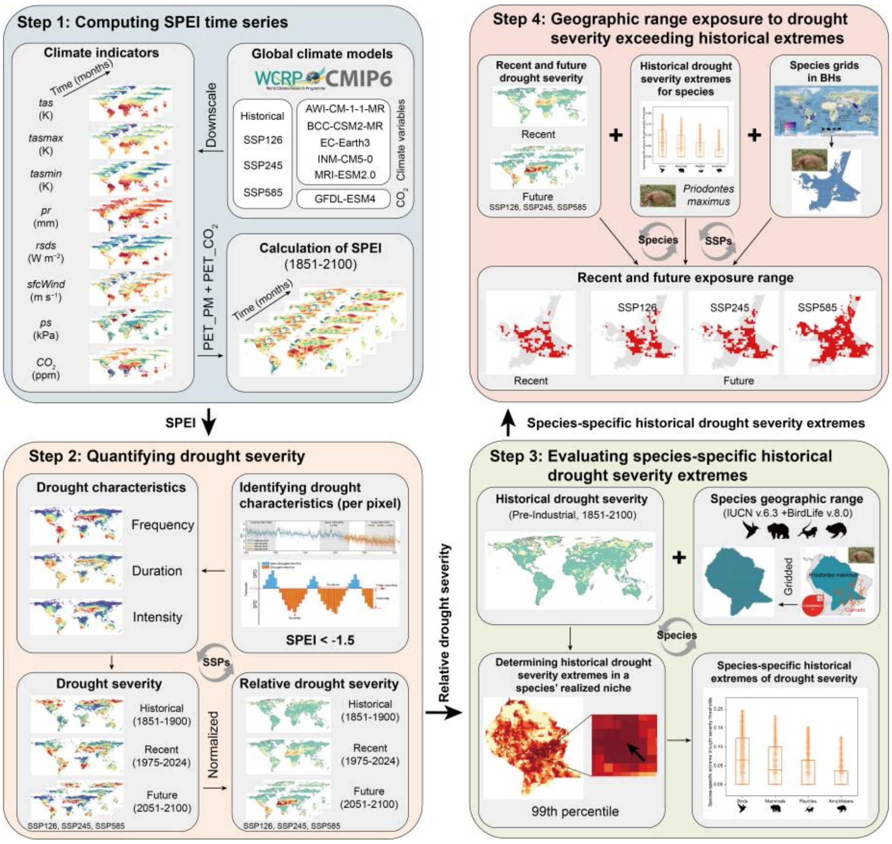

***Supplementary Fig. 1 Overview of methods used for assessing species geographic range exposure to drought severity beyond realized niche limits.** Illustration using the geographic range of Priodontes maximus as a vulnerable species found in the Cerrado (image of Priodontes maximus from IUCN). **Step 1:** calculate monthly time series of the standardized precipitation-evapotranspiration index (SPEI) using GCMs data from CMIP6, where tas represents the monthly mean near-surface air temperature, tasmax represents the monthly maximum near-surface air temperature, tasmin represents the monthly minimum near-surface air temperature, pr represents the monthly total precipitation, rsds represents the monthly total surface shortwave radiation, sfcWind represents the monthly mean near-surface wind speed, ps represents the monthly mean surface air pressure, and CO2 represents atmospheric carbon dioxide concentration; **Step 2:** quantify drought severity with a threshold of SPEI < –1.5; **Step 3:** evaluate species-specific historical drought severity extremes based on historical realized niche limits; **Step 4:** assess species geographic range exposure to drought severity exceeding historical extremes.*

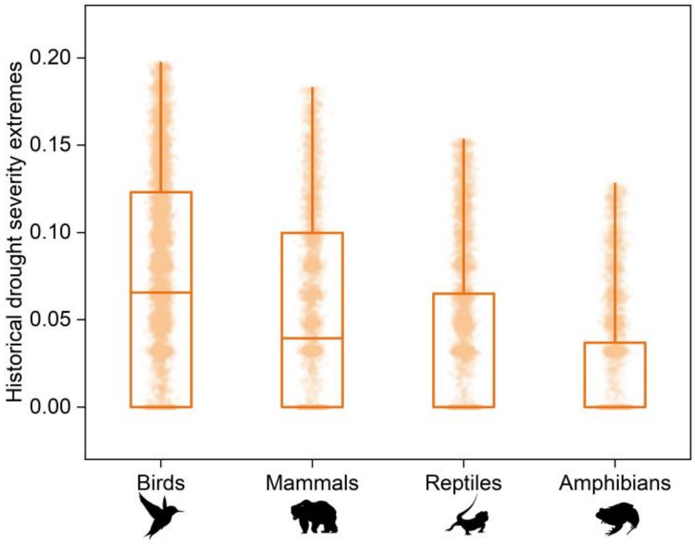

Supplementary Fig. 20 Species-specific historical drought severity extremes for terrestrial vertebrates. *The box plot shows the median and the 25th and 75th percentiles, with whiskers representing the 5th and 95th percentiles.*

Comment #13: there is quite some literature and debate about SSP585 not being a realistic scenario anymore because of the current reneweable deployment etc.

Response: Thank you for highlighting this important information. We agree that SSP585 is no longer a realistic scenario given current renewable energy deployment and other factors. According to the Intergovernmental Panel on Climate Change: Sixth Assessment Report (https://www.congress.gov/crs-product/R47082?utm=), the current warming trajectory closely resembles SSP245.

Therefore, we have highlighted the more realistic SSP245 scenario in our revised manuscript (L110–112). However, we did not exclude the SSP585 scenario, as it encompasses all possible future anthropogenic radiative forcing (Sarofim et al., 2024).

Sarofim, M. C. et al. High radiative forcing climate scenario relevance analyzed with a ten-million-member ensemble. Nature Communications 15 (2024).

L110–112: *Since the current warming trajectory closely resembles SSP245[7], the predicted intensification of drought severity may impact species persistence in BHs[13-18].*

Comment #14: again, not sure what the “concept” behind this term and how you use it is. I do not think it matches here.

Response: Thanks. We have removed the phrase “species drought crises” throughout the manuscript and clarified our research focus to drought exposure.

Comment #15: what is the rationale behind this number? I would think that it depends very much on the species whether loosing 50% of the range is a threat to the species (in terms of extinction) or not.

Response: Thank you for this insightful comment. We agree that whether exposure ≥50% of geographic range poses a threat to species survival depends on species-specific traits.

Our refined analysis incorporates drought vulnerability into the assessment of species' drought exposure (see response to comment #12). We assessed the geographic range exposure to drought severity exceeding historical extremes for each species. We further calculated the percentage of species exposed in at least half (≥50%) of their geographic range aiming to provide a comparable summary indicator of biodiversity range loss (Warren et al., 2018).

Warren, R., Price, J., Graham, E., Forstenhaeusler, N. & VanDerWal, J. The projected effect on insects, vertebrates, and plants of limiting global warming to 1.5°C rather than 2°C. Science 360, 791-795 (2018).

We have moved the approach for synthesizing drought exposure to the *Methods* and revised it accordingly (L490–496).

L490–496: *Drought exposure of species was synthesized in two complementary metrics: (1) the average percentage of current geographic range in BHs exposed to drought severity exceeding historical extremes across all species; and (2) the percentage of species exposed to drought severity exceeding their historical extremes across at least half (≥ 50%) of current geographic range in BHs, providing a comparable summary indicator of biodiversity range loss[37].*

Comment #16: Not sure what you want to express with this sentence (other than mentioning the number of threatened species covered

Response: Thank you for raising this concern. We recognized that a threatened conservation status does not imply greater drought vulnerability.

Therefore, we have removed this phrasing and instead introduced our focus on threatened terrestrial vertebrates in the *Introduction* (L85–87), while mentioning their numbers in the *Methods* (L371–373).

L85–87: *Focusing on of threatened terrestrial vertebrates (as identified by the International Union for Conservation of Nature[41]) within BHs, …*

L371–373: *…and 6,145 species of threatened terrestrial vertebrates, including 1,445 threatened birds, 970 threatened mammals, 1,276 threatened reptiles, and 2,454 threatened amphibians.*

Comment #17: sth missing

Response: Thanks. "Irano-Anatolian" is the official name for this BH. To avoid grammatical

ambiguity, we have used "Irano-Anatolian BH" throughout the text. As follows:

L135–138: *In the future, drought exposure is greater in mid-latitude dryland BHs (Fig. 3g, i), particularly in the Southwest Australia, Mediterranean Basin, Chilean Winter Rainfall and Valdivian Forests, Madrean Pine-Oak Woodlands, Irano-Anatolian BH, and Succulent Karoo.*

Comment #18: is this a special category for New Zealand? or why is it only mentioned here? was never introduced/defined before.

Response: Thank you for raising this issue. "endemic" was not intended to be a separate analytical category. To avoid confusion and align with our updated results, we have revised the text to consistently use "threatened species". As follows:

L141–146: *On average, threatened reptiles and amphibians are more vulnerable to drought exposure than birds and mammals in most BHs, especially in the Caucasus, Cerrado, Mountains of Southwest China, Madagascar and the Indian Ocean Islands, and Forests of East Australia. Nonetheless, threatened mammals face greater exposure than other taxa in the Maputaland-Pondoland-Albany, North American Coastal Plain, Atlantic Forest, Mesoamerica, and Caribbean Islands.*

Comment #19: but in your analyiss the entire difference is because of the warming which is behind. it is actually unclear whether low-warming impacts will not also stringly affect biodiversity because of the high demand for bionegery for example (see Hof et al.). https://doi.org/10.1073/pnas.1807745115

Response: Thank you for this comment. We revised the manuscript to explicitly acknowledge this trade-off: while SSP126 reduces climate exposure, the land-use requirements for bioenergy under this scenario may introduce new habitat threats. We now present SSP126 as a 'climate-safe' but not necessarily 'habitat-safe' scenario without careful land-use planning. As follows:

L165–168: *Moreover, the expansion of bioenergy cropland[42] and renewable energy[31] promoted by climate mitigation policies may accelerate habitat losses in BHs, potentially offsetting the biodiversity gains from slowing warming.*

Comment #20: Again, I would use more appropriate terms than crises and disaster.

Response: Thanks. We have removed these phrases and revised the sentence as follows:

L171–173: *Since climate mitigation strategies cannot fully reverse the future increases in drought exposure for species, spatially tailoring conservation efforts is crucial for facilitating species adaptation to drought exposure[43-45].*

Comment #21: I do not understand the connection of this finding to the 30 by 30 target?

Response: We recognize that the original manuscript did not explicitly articulate the connection between our findings and the "30×30" target.

In fact, we assessed whether areas facing drought exposure are protected. By comparing this conservation status with the "30×30" target, we aim to underscore that conservation decisions advancing the "30×30" target may require prioritizing areas with higher drought exposure, particularly in mid-latitude dryland BHs.

We have revised the manuscript as follows:

L176–179: *Most individual BHs projected to experience greater drought exposure are covered by protected areas below 15%, well below the global target of protecting at least 30% of the key ecological areas by 2030 ("30×30" target)[4], particularly for the Caucasus, Mountains of Central Asia, and Irano-Anatolian BH.*

L246–249: *In terms of policy, meeting the GBF's "30×30" target may benefit vulnerable BHs[4], but specific conservation decisions may require prioritizing mid-latitude dryland BHs that are more vulnerable to drought exposure.*

Comment #22: why "would"

Response: We have corrected the mistake as follows:

L181–184: *Indeed, BHs with greater drought exposure are situated mainly in transboundary areas of developing countries (Supplementary Fig. 14a, b, and Supplementary Table 1), where nearly 700 million people (Supplementary Fig. 14c) impose intensive human pressure on 77.5% of the area (human footprint score > 4[30], Fig. 4b).*

Comment #23: But this is also already going on in these regions. habitat loss and fragmentation etc. does not start when an area is protected but is already ongoing and may or may not stop when an area is declared a protected area.

Response: We appreciate your important comment. We have refined this argument to clarify that protected areas have mixed effectiveness in resisting habitat loss and do not insulate species from drought exposure driven by climate warming. Our revisions focus on highlighting that conservation gaps may stem from intense human pressures in BHs, where accelerating anthropogenic habitat loss and fragmentation are reducing opportunities for species to adapt to and survive in response to drought exposure, while conservation efforts may be constrained by trade-offs with other societal priorities. Specific revisions are as follows:

L170–198:
Conservation gaps and challenges
Since climate mitigation strategies cannot fully reverse the future increases in drought exposure for species, spatially tailoring conservation efforts is crucial for facilitating species adaptation

to drought exposure[43-45]. By overlaying protected area networks, we found that only ~11% (recent: 12.1%; SSP245: 10.8%; SSP585: 11.1%; SSP126: 11.6%) of areas facing drought exposure in BHs are currently protected (Fig. 4a and Supplementary Fig. 13). Most individual BHs projected to experience greater drought exposure are covered by protected areas below 15%, well below the global target of protecting at least 30% of the key ecological areas by 2030 ("30×30" target)[4], particularly for the Caucasus, Mountains of Central Asia, and Irano-Anatolian BH. This inadequate protection is presumably due to intense human competition for land use[46]. Indeed, BHs with greater drought exposure are situated mainly in transboundary areas of developing countries (Supplementary Fig. 14a, b, and Supplementary Table 1), where nearly 700 million people (Supplementary Fig. 14c) impose intensive human pressure on 77.5% of the area (human footprint score > 4[30], Fig. 4b). Furthermore, numerous evidence demonstrated that anthropogenic habitat conversion in BHs will be further amplified in the future[26,27,39]. Habitat loss and fragmentation from expanding human activities are leading to structural disarticulation across BHs, undermining the potential adaptive strategies of species to drought (such as migratory behavior)[25,26,33]. Therefore, while expanding protected networks does not insulate against drought exposure driven by climate warming, it can support species persistence by providing refuge and maintaining habitat integrity and connectivity[43,45]. However, protected areas have shown mixed outcomes in resisting habitat loss[47], and the implementation of conservation actions often entails trade-offs with other social priorities, including food production and economic development[28,48]. Additionally, applying top-down approaches to create protected areas may trigger conflicts with local livelihoods[28,29,48]. Consequently, understanding and minimizing these trade-offs in conservation decisions will be key to coping with intensifying drought exposure without compromising social well-being in BHs.

Comment #24: I think you have to come up with a more differentiated argumentation here. the more advanced literatur and thinking on nature's contribution to people highlighty that healthy ecosystems and protected areas are required for allowing development and fostering human rights. the perspective of land competition from conservation is an issue of how the protected area is designed and implemented and supported etc. and not an issue of protecting areas per se. so as long as you do not clearly define what a "one-site-fits-all" protection is, your argument of human right abuse is hard to maintain.

I think what you mean is a top-down designation of protected areas, maybe even with a colonial dimension, that infringes on indigenous people's rights etc.

but this can not be generalised as "the one conservation approach" as it depends very much on context and there is already ample literature on how to design protected areas in a more fair and equitable way.

Response: Thank you for your insightful comment. We agree that conflicts between protected areas and development or human rights arise from issues relating to the design, implementation, and support of protected areas.

The revised manuscript improves our narrative by first emphasizing that implementation of

conservation actions may be constrained by substantial development and livelihood needs in BHs, and further clarifying that top-down approaches to creating protected areas may trigger conflicts with local livelihoods (L191–195). Additionally, we have strengthened discussions on expanding adaptive conservation actions while promoting development and safeguarding human rights (L249–261). The revised contents are as follows:

L191–195: *However, protected areas have shown mixed outcomes in resisting habitat loss*[47]*, and the implementation of conservation actions often entails trade-offs with other social priorities, including food production and economic development*[28,48]*. Additionally, applying top-down approaches to create protected areas may trigger conflicts with local livelihoods*[28,29,48]*.*

L249–261: *Emphatically, BHs facing greater drought exposure are also bearing substantial social burdens, implying that conservation efforts may be constrained by weak local governance capacity and substantial livelihood and development needs*[28,48]*, such as planning ecological corridors on land also valuable for agriculture or infrastructure*[29]*. Therefore, expanding protection networks in BHs to adapt to intensifying drought exposure entails integrating social well-being. To this end, promoting land-use intensification or de-intensification in suitable areas could simultaneously restore natural habitats while matching land-use demands for socioeconomic development*[62,63]*. Moreover, strengthening cost-sharing and fiscal transfer mechanisms for conservation*[64]*, facilitating transboundary collaboration*[65]*, and recognizing conservation approaches rooted in indigenous and local communities*[66] *are essential for reducing conflicts and improving governance, particularly in BHs that share habitats or water systems.*

Comment #25: Again, this sentence is too convoluted. also see my earlier comment about mitigate and adapt.

Response: Thanks. We have restructured our wording:

L196–198: *Consequently, understanding and minimizing these trade-offs in conservation decisions will be key to coping with intensifying drought exposure without compromising social well-being in BHs.*

Discussion:

Comment #26: see my comment above on RCP8.5 /SSP585.

Response: We have modified the content accordingly:

L206–207: *…with this proportion projected to rise to 36.4% under a more realistic future scenario (SSP245).*

Comment #27: This is of course valid but how can these species-specific aspects really be assessed at a 0.5° resolution? All small to mid-scale habitat variation (can be large-scale for small animals with small dispersal rates) is basically ignored at this resolution.

Response: Thank you for raising this critical question. To assess the exposure of species geographic ranges to drought severity exceeding historical extremes, we gridded species distribution polygons into 0.5° grid cells. We selected a 0.5° resolution to capture the local climate variation across species ranges while avoiding false species presence wherever possible (Bütikofer et al., 2020; Hurlbert et al., 2007). We acknowledge that 0.5° resolution cannot identify fine-scale habitat heterogeneity, microhabitat variation, or local climate refuges, particularly for small species or those with limited dispersal capacity. However, we did not exclude these small-range species, as they may be more vulnerable to climate extremes (Urban, 2015; Hurlbert et al., 2007).

Bütikofer, L. et al. The problem of scale in predicting biological responses to climate. Global Change Biol 26, 6657-6666 (2020).

Hurlbert, A. H. & Jetz, W. Species richness, hotspots, and the scale dependence of range maps in ecology and conservation. P Natl Acad Sci USA 104, 13384-13389 (2007).

Urban, M. C. Accelerating extinction risk from climate change. Science 348, 571-573 (2015).

Warren, R. et al. Quantifying the benefit of early climate change mitigation in avoiding biodiversity loss. Nature Climate Change 3, 678-682 (2013).

We have discussed in the manuscript that ecological processes, such as species utilizing microhabitats or tolerating new habitats, may alter our estimates (L267–270).

Importantly, we supplemented a spatial scale effect analysis into our study to confirm the robustness of our drought exposure assessment (L504–524).

L267–270: *Some species may buffer drought exposure through behavioral adaptations or phenotypic plasticity, such as utilizing microhabitats[50], shifting seasonal activities[67], adapting new habitats to alter current range[56], or evolving physiological tolerance to drought[13].*

L504–524:

Spatial scale effect

We assessed the species exposure to intensifying drought using climate model data at 0.5° resolution, aiming to balance the need to capture spatial climate details while avoiding false species presence[76,81]. However, 0.5° grid cells may contain considerable spatial climate heterogeneity, causing discrepancies between our analyzed drought conditions and the local microclimates that influence organism survival[92]. To this end, we examined the relationship between drought exposure in each grid cell and its internal spatial climate heterogeneity. Spatial climate heterogeneity was calculated as the standard deviation of the 1-km resolution aridity index within each grid cell, reflecting regional long-term climate characteristics[93]. Habitats with higher spatial climate heterogeneity in BHs exhibit more moderate drought exposure (Supplementary Fig. 21), indicating that our findings still identified those habitats with more diverse climatic conditions (such as mountain communities) as better buffers against

drought threats. Importantly, while the use of finer-resolution climate data may introduce biases in estimates of drought exposure, it does not change the central conclusion that species are increasingly exposed to drought severity beyond their realized niche limits, and that limiting future warming can reduce exposure. The reason is that drought exposure tends to be greater in habitats with relatively low spatial climate heterogeneity. Nevertheless, adopting finer-scale climate data that more closely reflects the conditions actually experienced by organisms may improve the accuracy with which the impacts of intensifying drought on biodiversity are represented[76,81].

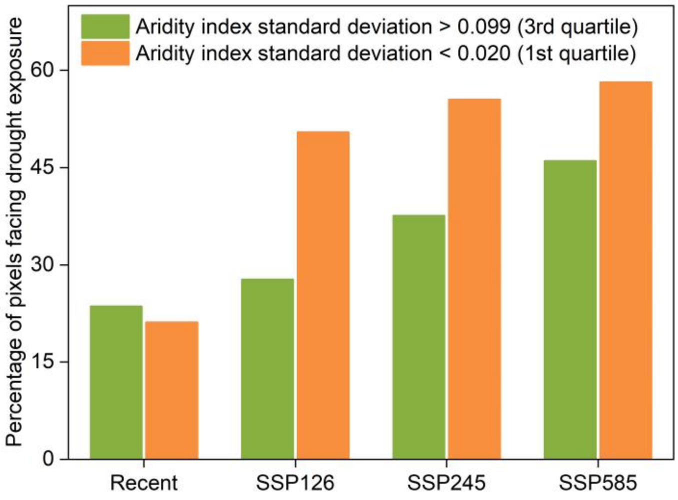

Supplementary Fig. 21 Drought exposure of threatened terrestrial vertebrate habitats in 0.5° grid cells with low or high spatial climate heterogeneity. *Spatial climate heterogeneity was calculated as the standard deviation of the 1-km resolution aridity index within each grid cell, reflecting regional long-term climate characteristics. Aridity index standard deviation below the 1st quartile indicates lower spatial climate heterogeneity, and above the 3rd quartile indicates higher spatial climate heterogeneity.*

Comment #28: Not sure what you want to say here....

Response: We have restructured our text to clearly articulate that climate mitigation strategies cannot fully curb the future increases in drought exposure of species relative to recent levels, thereby highlighting the necessity of expanding adaptive conservation actions. The revised text is below:

L236–237: *Even so, under an optimistic warming scenario, future drought exposure remains greater than recent levels.*

Methods:

Comment #29: This is a bit too simplified. I think you ignore CO2 effects on water use efficiency which affects drought. this effect will be much higher under rcp8.5 and counter the negative impacts of the warming to some extent. co2-effects can be included in the PET

calculation.

Response: Thanks. According to your helpful suggestion, we have rerun our PET calculation incorporating the effects of CO_2. The details are as follows:

L412–427: *PET formulation usually employs the Penman-Monteith equation (PET_PM)[84] and one of its variants that accounts for decreased evapotranspiration due to increased surface resistance driven by rising atmospheric CO_2 concentrations (PET_CO2)[85], which are respectively parameterized as:*

$$PET\_PM = \frac{0.408\Delta(R_n - G) + \gamma \frac{900}{T_{avg} + 273} u_2(e_s - e_a)}{\Delta + \gamma(1 + 0.34u_2)} \quad (1)$$

$$PET\_CO_2 = \frac{0.408\Delta(R_n - G) + \gamma \frac{900}{T_{avg} + 273} u_2(e_s - e_a)}{\Delta + \gamma(1 + (0.34 + 2.4 \times 10^{-4}([CO_2] - 300))u_2)} \quad (2)$$

where Δ is the gradient of the saturated vapor pressure curve, R_n is the net radiation at the crop surface, G is the soil heat flux density, γ is the psychrometric constant, T_{avg} is the near-surface temperature, u_2 is the near-surface wind speed, $e_s - e_a$ is the saturated and actual vapor pressure deficit, these parameters can be derived from the climate variables collected from GCMs of CMIP6. Atmospheric CO_2 concentration ($[CO_2]$) grid data were obtained from the GFDL-ESM4 model in CMIP6, which shows strong correlation with observations from the Global Monitoring Laboratory (Pearson's $r > 0.99$, $p < 0.001$, Supplementary Fig. 18). Notably, the PET_CO_2 is modeled based on non-water-limited surface[85]. Therefore, we estimated PET using the average of the PET_PM and PET_CO_2 models for global drylands[19] (as defined by UNEP-WCM), while employing the PET_CO_2 model for other regions.

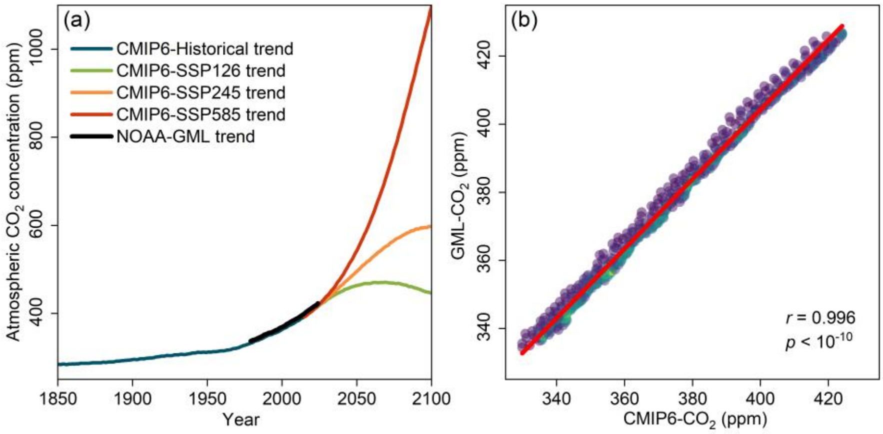

Supplementary Fig. 18 Trends and validation of the atmospheric CO2 concentrations. a, *Global atmospheric CO2 concentration trends for the GFDL-ESM4 model of CMIP6 (1851 to 2100) and the Global Monitoring Laboratory (GML) of the National Oceanic and Atmospheric Administration (NOAA) (1979 to 2024, https://gml.noaa.gov/ccgg/trends/gl_data.html).* ***b,*** *Validation of the atmospheric CO_2 concentration data from the GFDL-ESM4 model for the historical period (1979 to 2024) using*

observational data provided by the NOAA-GML. Red line represents linear regression; Pearson's correlation coefficients (r) and two-sided unadjusted p values are provided.

Comment #30: But this means that you do not really know whether the system is affected by droughts operating at longer cycles than the 20 years you study. historical megadroughts may have much longer return intervals and still shape ecosystems.

Response: Thank you for this important comment. Following your suggestion regarding the historical baseline, our study focused on the impacts of intensifying droughts on biodiversity since the onset of large-scale anthropogenic warming. Given the increasing instability of dominant cycles of global climate extremes (Zantout et al., 2025), we expanded our original 20-year timescale to 50-year to capture drought events wherever possible.

We have now acknowledged and discussed that historical megadroughts with recurrence intervals longer than our explored timescale may influence our assessment. Specific revisions are as follows:

Zantout, K. et al. Shifting dominant periods in extreme climate impacts under global warming. Nature Communications 16 (2025).

L457–459: *Notably, the dominant cycles of extreme weather are becoming increasingly unstable under global warming[87], and historical megadroughts with recurrence intervals longer than 50 years may still shape ecosystems[88].*

Comment #31: this is exactly my point above: how can you be sure that the 2010s are actually a good representation of the historical record. in the Californian juniperon-pinion ecosystems, which have experienced a lot of drought mortality, research suggest there are long drought cycles to be considered for example, even though the recent droughts have been devastating. So actually the 2010s period is grossly out of balance with what seems to have been a more stationary state before. and you take this period as baseline.

Response: Following your valuable comments, we have redefined the historical baseline as the pre-industrial period (1851–1900). Using this new historical baseline, we have quantified species-specific historical drought severity extremes (see response to comment #12). The revision is as follows:

L475–478: *Importantly, considering that recent droughts have already imposed significant impacts on ecosystems[14-16,21,22], we use the pre-industrial period (1851 to 1900, characterized by relatively stable climate conditions) as the historical baseline.*

Reviewer #2 (Remarks to the Author):

This manuscript presents an ambitious and valuable global assessment of projected changes in drought severity across biodiversity hotspots and their implications for terrestrial vertebrates. The integration of multiple SSP scenarios and the attempt to link drought dynamics with biodiversity and socio-economic dimensions represent important contributions to understanding climate–biodiversity interactions. Further, the figures are very informative. While the study complements recent research, with a distinct method of drought quantification and a broader framing, several aspects require substantial revision before the manuscript is suitable for publication.

The manuscript would benefit from clearer and more concise writing — the text is often repetitive and overloaded with adjectives, which obscures the main messages. Substantively, I have two major concerns. First, while inclusion of three SSPs is commendable, the use of SSP585 as the leading scenario warrants reconsideration given the growing consensus that it represents an increasingly implausible high-emission pathway. Second, the drought threshold adopted (SPEI < –0.5) is extremely low; such values indicate only mildly dry or near-normal conditions and may not meaningfully reflect ecological drought stress. Using this as a starting point for quantifying drought frequency and severity likely inflates drought intensity estimates and, consequently, the extent of areas and species deemed exposed to "extreme drought."

Response: Dear reviewer, we very much appreciate your thorough evaluation and insightful suggestions on our work. We have addressed all your concerns in detail and made extensive amendments to our manuscript. Please do not hesitate to contact us if further material or information is needed.

Title:

Comment #1: I would avoid the use of 'significantly' in a non-statistical context. Perhaps 'Representative Concentration PathwayS determine future drought exposure for terrestrial vertebrates'.

Response: Thanks. Following your and reviewer #1's valuable suggestions, we have revised the title to "**Limiting future warming reduces drought exposure for terrestrial vertebrates**".

Abstract:

Comment #2: L30: exposure to drought frequency, intensity, or both?

Response: Our research focuses on the dynamics of drought severity, which is the combination of drought frequency, duration, and intensity. We have revised the original sentence to:

L28–29: *Ambitious and adaptive conservation efforts are needed to curb biodiversity loss, particularly as drought severity intensifies globally.*

Comment #3: L31: meaning of 'beyond historical records' is not entirely clear here. Droughts more extreme than in the historical records, or droughts predicted in the future?

Response: Thank you for raising this ambiguity in our wording. According to your subsequent important comments, our refined analysis assessed the exposure of species' geographic range to recent and future drought severity exceeding the extremes experienced during their pre-industrial history (see response to comment 35). Therefore, we have revised our wording:

L29–31: *Here, we assess the exposure of terrestrial vertebrates within global biodiversity hotspots (BHs) to recent and future drought severity surpassing the extremes experienced during their pre-industrial history.*

Comment #4: L40: conservation gaps? As in, conservation issues?

Response: We have revised the sentence to clearly express that most areas facing drought exposure are currently inadequately protected. As follows:

L39–42: *However, given that future drought exposure will increase in most BHs, and exposed regions currently face inadequate protection and substantial social burdens, expanding adaptive conservation actions without compromising local well-being is urgent.*

Comment #5: L41: social equity appears a bit out of the blue and an afterthought, if there is space, there should be a line on how conservation money is not allocated according to spatial patterns of biodiversity.

Response: Thank you for this constructive suggestion. We have adjusted the narrative as follows:

Revised manuscript

L39–42: *However, given that future drought exposure will increase in most BHs, and exposed regions currently face inadequate protection and substantial social burdens, expanding adaptive conservation actions without compromising local well-being is urgent.*

Introduction:

Comment #6: General: the writing of the introduction, results, and discussion stands to be improved. Firstly, there are phrasing and sentence structure issues. Further, the writing could be more concise. I provide examples for the first paragraph of the introduction:

1-The first sentence is 50 words with many nested clauses, making it difficult to follow.

2- 'skepticism is ongoing' (awkward phrasing)

3- 'Despite their immense value' (either omit this or expand upon it, it's just awkwardly

postulated here)

4- 'Increased future extreme drought will doubtless pose a considerable threat to species in critical but fragile BHs' (overabundance of adjectives, almost half of the word count)

5- 'Recurring extreme drought will potentially have unexpected negative cascading effects on ecosystems' (half of the words are adjectives)

6- L58–60 and 60–62 essentially say the same thing

7- 'The planet's exceptionally species rich....' sounds awkward because of the unnecessary adjective. The planet is not exceptionally rich in species; it's just the only planet with a known biological diversity.

Response: Thanks for this comment. We have conducted a thorough line-by-line edit to remove 'adjective bloat' and simplify sentence structures throughout the manuscript, specifically addressing the examples you provided. The revised first paragraph of the *Introduction* is as follows:

L46–59: *Prioritizing and safeguarding global biodiversity hotspots (BHs, the most species-rich yet highly threatened terrestrial regions[1-3]. Fig. 1) is central to the Kunming-Montreal Global Biodiversity Framework (GBF)'s ambitions of halting and reversing biodiversity loss[4]. However, the conservation priorities for BHs under accelerating climate warming remain unclear[3,5,6]. Anthropogenic climate change is exacerbating the frequency, duration, and intensity of drought events globally at an unprecedented rate and is expected to further increase in the future[7-12]. Recurring droughts degrade habitat quality, directly affecting species survival strategies, increasing physiological stresses such as heat stress and dehydration, causing reproduction failure and population decrease[13-18], and triggering negative cascading effects in ecosystems19-22. Recent extreme droughts have already resulted in habitat loss and population die-offs in localized areas within BHs[3,16,21,23]. Therefore, evaluating the impacts of intensifying drought on biodiversity in critical yet vulnerable BHs is fundamental to guiding pre-emptive conservation efforts[3,24].*

Comment #7: L66–68: there is no clear link between the first statement (inequitable conservation spending) and the second (drought as a compounding threat to biodiversity).

Response: We have revised the second paragraph of the *Introduction*, see our response to comment #9 for details.

Comment #8: L71: informal use of unfortunately (and another adjective).

Response: Thanks. We have revised this informal wording, see our response to comment #9.

Comment #9: L64–78: The overarching message (social equity issues exacerbate threats to BHs

already posed by climatic change and habitat loss) is not immediately clear because the paragraph subject seems to change throughout (e.g. drought is mentioned in the first sentence, then we switch to habitat loss, go back to drought, and in between we mention issues of social equity). A more logical approach could be;

1- habitat loss and fragmentation considered as primary threats

2- climatic change, one of its facets being extreme drought, interacting and compounding with these threats

3- how this is all further problematized by social equity issues.

Response: Thank you very much for this constructive suggestion. We have restructured the second paragraph of the *Introduction* accordingly.

Firstly, we now emphasized that habitat loss and fragmentation weaken the opportunities for species to adapt and survive in the face of drought threats. We further indicated that these anthropogenic pressures result from substantial development and livelihood needs in BHs. Finally, we highlighted the importance of clarifying conservation gaps and governance challenges of drought threats for developing equitable and adaptive conservation strategies.

The specific revisions are as follows:

L61–73: *Although the threat of droughts to species and ecosystem viability is widely recognized[13-21], their ecological consequences may be further amplified by reduced opportunities for species to adapt and survive due to anthropogenic habitat loss and fragmentation[25-27]. In fact, most BHs are located in densely populated and underdeveloped regions[2,3], where increasing livelihood needs often compete with biodiversity conservation in terms of land use and financial resources[5,28,29]. Habitat deterioration caused by extensive urbanization[30], agricultural and renewable energy expansion[25,31], deforestation[5], or transboundary fencing[32] is undermining potential climate refuges for species and weakening their dispersal to track their climate envelopes[25,33]. Consequently, identifying the species and regions most affected by intensifying drought in BHs, and clarifying their conservation gaps and governance challenges, can inform decisions to develop or modify equitable and adaptive conservation measures[5,34].*

Comment #10: again, careful proofreading and rewriting necessary: 'such studies have not yet focused on…', remove 'yet' as these studies are already published and cannot be amended.

Response: Thanks. We have corrected the wording, see our response to comment #11.

Comment #11: L82–85: I believe this overstates the novelty of the study, because what you state to be novel has largely been done here - https://onlinelibrary.wiley.com/doi/full/10.1111/gcb.70272.

Your study still has novelty, and is complementary, because it

1- focusses largely on vertebrates with an already threatened status (as opposed to e.g. Least Concern species that nevertheless have 'Drought' assigned as a conservation threat by IUCN)

2- looks at drought exposure differently

3- zooms in on the governance challenges posed by the results

The paper linked above is a good opportunity to acknowledge recent findings on this topic and further contextualize your study.

Response: Thank you for the constructive comment. We acknowledge that the original manuscript overstated the novelty due to missing previous literature on the impacts of climate extremes including droughts in BHs. Incorporating your important suggestions, we have revisited previous research, improved our analysis, and repositioned our study.

We have now clarified that previous assessments primarily relied on localized climate anomalies or fixed historical thresholds to assess the impacts of intensifying drought on biodiversity, overlooking species-specific drought vulnerability. To this end, we reference a recent research framework on temperature extremes (Trisos et al. 2020; Murali et al. 2023), utilizing historical drought severity extremes within species realized climatic niches to approximate their drought tolerance limits. We further assessed the exposure of species to recent and future drought severity exceeding their historical extremes.

Furthermore, we have highlighted that our research primarily focuses on threatened terrestrial vertebrates in BHs, further reveal conservation gaps and governance challenges in areas facing drought exposure.

Trisos, C. H., Merow, C. & Pigot, A. L. The projected timing of abrupt ecological disruption from climate change. Nature 580, 496-501 (2020).

Murali, G., Iwamura, T., Meiri, S. & Roll, U. Future temperature extremes threaten land vertebrates. Nature 615, 461-467 (2023).

The specific revisions are as follows:

L75–89: *Previous studies integrating climate change simulations with species distribution modeling demonstrate that species, both globally and within BHs, are increasingly exposed to droughts and other climate extremes*[13,20,35-40]*. However, most existing assessments quantify these impacts using generalized climate anomalies or fixed historical thresholds*[18,40]*. While such approaches capture intensifying drought conditions, they often overlook the species-specific differences in drought vulnerability, which are critical determinants of survival*[18,40]*. To address the scarcity of physiological data for most species*[40]*, we estimated drought tolerance limits using the historical extremes of species realized climatic niches. We then assessed the exposure of species' geographic ranges to recent and future drought severity exceeding their historical extremes (see Supplementary Fig. 1 and Methods). Focusing on threatened terrestrial vertebrates (as identified by the International Union for Conservation of Nature*[41]*) within BHs, this analysis identifies critical conservation gaps and governance challenges in regions facing drought exposure, providing spatially explicit guidance for conservation planning.*

Comment #12: L87: again, not sure what 'beyond the historical record' means, though I think you mean they surpass historical drought exposure.

Response: Thanks. Our refined analysis assessed the exposure of species' geographic ranges to recent and future drought severity exceeding their historical extremes. We have revised our phrasing as follows:

L81–85: *To address the scarcity of physiological data for most species[40], we estimated drought tolerance limits using the historical extremes of species realized climatic niches. We then assessed the exposure of species' geographic ranges to recent and future drought severity exceeding their historical extremes (see Supplementary Fig. 1 and Methods).*

Results:

Comment #13: General: again, I believe there are many opportunities to tighten the writing:

unprecedented future expansion of drought risk = increased drought risk

the risk of future drought = future drought risk

to visually indicate = to visualize

the degree of exposure to extreme drought = extreme drought exposure

after we aggregated all future climate scenarios = after aggregating climate scenarios, etc.

Response: Thank you very much. We have checked and tightened the writing throughout the text.

Comment #14: L106: do you mean compared to the global average?

Response: Yes, we compared the average drought severity in BHs with the global terrestrial average. We have further revised the wording:

L102–104: *Relative to the historical baseline, average drought severity in BHs has recently increased by 48.9% and is projected to exceed the global terrestrial average in the future (Fig. 2e).*

Comment #15: L115–118: I would lead all findings by reporting results under the intermediate SSP, followed by the extremes (as some sort of 'confidence interval'). One reason is that SSP 585 criticized for not being very realistic (https://doi.org/10.1038/s41467-024-52437-9). It projects virtually no GGE mitigations even though such initiatives have been underway globally (though, of course, not in desirable degrees).

Response: Thank you for pointing out this important information. We agree that SSP585 is not

very realistic.

Following your suggestion, we have prioritized reporting future projections under the more realistic SSP245 scenario, followed by the results under pessimistic (SSP585) and optimistic (SSP126) scenarios. As follows:

L104–108: *Under SSP245, nearly half (46.0%) of the areas in BHs will experience drought severity above historical levels, increasing to 51.0% under SSP585 (Fig. 2f and Supplementary Fig. 5). Even under the stringent climate mitigation scenario (SSP126), this proportion remains substantial at 38.8%.*

Comment #16: L121–122: I'm not sure the IPCC report states this anywhere?

see https://www.congress.gov/crs-product/R47082?utm=, paragraph below Table 1

Response: Thanks. We have corrected our view based on the latest IPCC report, emphasizing that the current warming trajectory closely resembles SSP245. As follows:

L110–112: *Since the current warming trajectory closely resembles SSP245[7], the predicted intensification of drought severity may impact species persistence in BHs[13-18].*

Comment #17: L130: it is currently unclear in this paragraph whether you also quantified exposure in these two ways.

Response: Thank you for raising this issue. We employed these two ways to provide summary indicators of species' drought exposure. Exposure is quantified based on whether the recent or future drought severity in grid cells across species geographic range exceeds their historical extremes.

We have revised the relevant wording for clarity and moved it to the *Methods*. As follows:

L488–496: *We mapped drought exposure for each species by identifying whether recent or future drought severity in grid cells across its current geographic range exceeds its historical extremes. Drought exposure of species was synthesized in two complementary metrics: (1) the average percentage of current geographic range in BHs exposed to drought severity exceeding historical extremes across all species; and (2) the percentage of species exposed to drought severity exceeding their historical extremes across at least half (≥ 50%) of current geographic range in BHs, providing a comparable summary indicator of biodiversity range loss[37].*

Comment #18: L135–136: more careful phrasing is needed here. A threatened conservation status does not imply higher drought vulnerability/sensitivity (see van den Bosch et al., 2025). I would also not specify 'vulnerable to extinction', as there are many things that precede extinction (distribution shifts, local extirpations, abundance declines, etc.).

Response: We agree that species defined as threatened by the IUCN does not imply higher

drought vulnerability, and we recognize that the term "extinction" was used imprecisely.

We have removed these statements and instead highlighted in the "Introduction" that our primary focus is on the threatened species as defined by the IUCN. As follows:

L85–87: *Focusing on of threatened terrestrial vertebrates (as identified by the International Union for Conservation of Nature[41]) within BHs, ...*

Comment #19: L140–143: to meaningfully interpret these numbers, you may want to compare it to a recent period.

Response: Following your important suggestion, we have incorporated the recent period into our refined analysis, quantifying and comparing the drought exposure of species between recent and future periods to more reasonably characterize the intensifying drought threats. The specific revisions are as follows:

L117–122: *The estimates indicate that an average of 22.7% of geographic range in BHs for threatened terrestrial vertebrates has been exposed to drought severity exceeding their historical extremes in recent decades (Fig. 3a). Under SSP245, by the latter half of the 21st century, the average percentage of exposure range is projected to increase to 35.9%, as high as 49.8% under SSP585. Although the average estimate is lower for SSP126 (28.2%), it remains above recent levels.*

Comment #20: L140–146: I got confused: for SSP585 you report mean range percentage exposed and percentage of species for which > 50% of the range is exposed, but for the other SSPs you appear to report different statistics? Again the mean range percentage but also the percentage of species exposed?

Response: We have restructured the paragraph to first report the average percentage of geographic range for species exposed to drought severity exceeding their historical extremes under different scenarios, followed by the percentage of species exposed to drought severity exceeding their historical extremes in at least half (> 50%) of their geographic range. The revised paragraph reads as follows:

L115–133: *We assessed the geographic range exposure of terrestrial vertebrates in BHs to recent and future drought severity exceeding their historical extremes (see Supplementary Fig. 1 and Methods). The estimates indicate that an average of 22.7% of geographic range in BHs for threatened terrestrial vertebrates has been exposed to drought severity exceeding their historical extremes in recent decades (Fig. 3a). Under SSP245, by the latter half of the 21st century, the average percentage of exposure range is projected to increase to 35.9%, as high as 49.8% under SSP585. Although the average estimate is lower for SSP126 (28.2%), it remains above recent levels. Across taxa, the average percentage of exposure range for threatened amphibians (recent: 29.0%; SSP245: 41.6%; SSP585: 60.9%; SSP126: 31.0%, Fig. 3e) and reptiles (recent: 24.2%; SSP245: 35.5%; SSP585: 45.3%; SSP126: 28.1%, Fig. 3d) are higher*

compared to threatened mammals (recent: 20.0%; SSP245: 33.6%; SSP585: 38.4%; SSP126: 28.9%, Fig. 3c) and birds (recent: 12.6%; SSP245: 28.1%; SSP585: 42.4%; SSP126: 23.1%, Fig. 3b). Particularly, the average exposure estimates for threatened terrestrial vertebrates are higher than other terrestrial vertebrates (Supplementary Fig. 8). Overall, 22.6% of threatened terrestrial vertebrates in BHs have recently experienced drought severity exceeding historical extremes across at least half (≥ 50%) of their geographic range, and this percentage of species will respectively rise to 36.4% (SSP245), 51.0% (SSP585), and 27.8% (SSP126) in the future (Fig. 3f).

Comment #21: L144: number missing in 'SSP 24x'

Response: Thanks. We have corrected this error and checked the full text.

Comment #22: L138–155: see previous comment about the order of SSPs in reporting results.

Response: We have adjusted the reporting order for results under different future scenarios. As follows:

L119–133: *Under SSP245, by the latter half of the 21st century, the average percentage of exposure range is projected to increase to 35.9%, as high as 49.8% under SSP585. Although the average estimate is lower for SSP126 (28.2%), it remains above recent levels. Across taxa, the average percentage of exposure range for threatened amphibians (recent: 29.0%; SSP245: 41.6%; SSP585: 60.9%; SSP126: 31.0%, Fig. 3e) and reptiles (recent: 24.2%; SSP245: 35.5%; SSP585: 45.3%; SSP126: 28.1%, Fig. 3d) are higher compared to threatened mammals (recent: 20.0%; SSP245: 33.6%; SSP585: 38.4%; SSP126: 28.9%, Fig. 3c) and birds (recent: 12.6%; SSP245: 28.1%; SSP585: 42.4%; SSP126: 23.1%, Fig. 3b). Particularly, the average exposure estimates for threatened terrestrial vertebrates are higher than other terrestrial vertebrates (Supplementary Fig. 8). Overall, 22.6% of threatened terrestrial vertebrates in BHs have recently experienced drought severity exceeding historical extremes across at least half (≥ 50%) of their geographic range, and this percentage of species will respectively rise to 36.4% (SSP245), 51.0% (SSP585), and 27.8% (SSP126) in the future (Fig. 3f).*

Comment #23: L157: I'm unsure what 'clustering' means in this regard.

Response: Given the uncertainty in future socioeconomic development pathways, we employed a hierarchical clustering statistical analysis (Murtagh & Legendre 2014) to create a predictive ensemble of exposure estimates across three scenarios, thereby identifying BHs facing higher drought exposure.

Murtagh, F. & Legendre, P. Ward's Hierarchical Agglomerative Clustering Method: Which Algorithms Implement Ward's Criterion? J Classif 31, 274-295 (2014).

We explain this process in the *Methods* (L499-503).

L496–500: *Given the uncertainty in future socioeconomic development pathways, we also employed hierarchical clustering analysis[91] to create an ensemble of projections according to exposure estimates under three scenarios (SSP126, SSP245, and SSP585) for each BH, thereby more conservatively identifying which BHs are vulnerable to drought exposure in the future.*

Comment #24: L166–171: just making sure we are basing this on proportions per species class, so adjusting for the fact some species will be richer in birds than mammals etc.?

Response: Thank you for your important clarification. Yes, all our comparisons across taxa are based on species percentages within each class (e.g., mammals, birds, reptiles, amphibians), rather than absolute species richness. This approach ensures that the reported differences in drought exposure are not affected by variations in species richness among taxa.

Comment #25: L179: the heading for this section 'benefits from sustainable development' strikes me as odd, I would explicitly link back to the SSPs.

Response: Thanks. We have adjusted the heading for this section to "**Benefits of limiting future warming**"

Comment #26: L184–192: the phrasing of 'reducing' and 'avoiding' exposure confuses me. What is the difference between them?

Response: Thank you for pointing out this issue in the phrasing. We summarized drought exposure for species in two ways (see response to comment #17). Here, "reducing" means the average reduction in range exposure, while "avoiding" refers to the percentage of species that can avoid exposure to at least half of their geographic range.

We have revised the sentence to emphasize primarily how much range exposure can be reduced on average by limiting future warming, thereby eliminating this confusion. As follows:

L151–155: *Generally, limiting mean global warming to below 1.8 °C above pre-industrial levels by the end of the 21st century (SSP126) can effectively reduce the average percentage of geographic range exposed to drought severity exceeding historical extremes respectively by 7.7% relative to SSP245 (2.7 °C warming) and 21.6% relative to SSP585 (4.4 °C warming) (Fig. 3a).*

Comment #27: L206: ok, but while protected status might prevent habitat loss, amongst other things, it does not immediately prevent climatic change in that area. Nor would granting protected status to BHs necessarily limit their future drought exposure. So this would need to be discussed a bit more in-depth.

Response: We agree that creating protected areas cannot prevent regional climate change. However, well-managed protected areas can facilitate species adaptation to climate change (Mi

et al., 2023; Buenafe et al., 2025). Our analysis indicates that even an optimistic warming scenario cannot fully reverse the future increases in drought exposure for species, highlighting the urgent need for spatially explicit adaptive conservation measures. To this end, we assessed the coverage of protected areas in areas facing drought exposure within BHs to support targeted optimization of conservation strategies.

Mi, C. R. et al. Global Protected Areas as refuges for amphibians and reptiles under climate change. Nature Communications 14 (2023).

Buenafe, K. C. V. et al. Current approaches and future opportunities for climate-smart protected areas. Nature Reviews Biodiversity, 1-14 (2025).

We have restructured our narrative to emphasize that establishing protected areas aims to facilitate species adaptation to intensifying drought exposure, rather than mitigate drought exposure. The specific revisions are as follows:

L171–173: *Since climate mitigation strategies cannot fully reverse the future increases in drought exposure for species, spatially tailoring conservation efforts is crucial for facilitating species adaptation to drought exposure*[43-45].

L189–191: *Therefore, while expanding protected networks does not insulate against drought exposure driven by climate warming, it can support species persistence by providing refuge and maintaining habitat integrity and connectivity*[43,45].

Comment #28: L208: what are local versus global BHs? These terms have not been clarified.

Response: Thanks. There are currently 36 BHs worldwide. Our intention was to describe the coverage of protected areas for all BHs and each individual BH. We have revised our wording:

L173–179: *By overlaying protected area networks, we found that only ~11% (recent: 12.1%; SSP245: 10.8%; SSP585: 11.1%; SSP126: 11.6%) of areas facing drought exposure in BHs are currently protected (Fig. 4a and Supplementary Fig. 13). Most individual BHs projected to experience greater drought exposure are covered by protected areas below 15%, well below the global target of protecting at least 30% of the key ecological areas by 2030 ("30×30" target)*[4]*, particularly for the Caucasus, Mountains of Central Asia, and Irano-Anatolian BH.*

Comment #29: L215–216: it should be mentioned the SSPs also project changing human populations and, by proxy, human footprints, which could not be accounted for with the static human footprint index.

Response: Thank you for your important comment. We admit we did not consider projected changes in human footprint under different SSPs. We focused on the climate component of exposure, while future land-use change is a separate threat layer that was outside the scope of this specific analysis. Nevertheless, based on prior research, we have supplemented evidence indicating that future anthropogenic habitat conversion in BHs will further intensify. As follows:

L184–186: *Furthermore, numerous evidence demonstrated that anthropogenic habitat conversion in BHs will be further amplified in the future*[26,27,39].

Comment #30: L217–221: the content is good, but it's not really fleshed out. It's not telling me how e.g. habitat loss or fencing things can exacerbate drought effects, and the point about human rights issues also remains quite vague.

Response: Thanks. We have supplemented the explanation of how habitat loss and fragmentation can affect species' adaptation to drought exposure. Additionally, we further discussed the potential conflicts between development and livelihood needs in BHs and conservation efforts. The specific revisions are as follows:

L186–195: *Habitat loss and fragmentation from expanding human activities are leading to structural disarticulation across BHs, undermining the potential adaptive strategies of species to drought (such as migratory behavior)*[25,26,33]. *Therefore, while expanding protected networks does not insulate against drought exposure driven by climate warming, it can support species persistence by providing refuge and maintaining habitat integrity and connectivity*[43,45]. *However, protected areas have shown mixed outcomes in resisting habitat loss*[47], *and the implementation of conservation actions often entails trade-offs with other social priorities, including food production and economic development*[28,48]. *Additionally, applying top-down approaches to create protected areas may trigger conflicts with local livelihoods*[28,29,48].

Discussion:

Comment #31: L229: what's the difference between 'threaten survival of species' and 'exacerbate extinction risk'?

Response: Thank you for pointing out this redundant phrasing. We have tightened the wording as follows:

L202–203: *Drought severity beyond niche limits is highly detrimental to species survival*[13-18].

Comment #32: L234: again, I don't think the IPCC report insinuates SSP585 resembles the current trajectory, and I highly recommend looking into considering SSP245 as your leading 'middle-of-the-road' scenario.

Response: Thanks. We have revised our narrative accordingly:

Revised manuscript

L206–207: *…with this proportion projected to rise to 36.4% under a more realistic future scenario (SSP245).*

Comment #33: L234–236: there again seems to be some repetition. I can't see the difference between these lines and lines L236–238.

Response: We have revised the sentences to reduce repetition, as follows:

L208–211: *Mid-latitude dryland BHs will face greater drought exposure, in which compound extreme thermal events may further amplify thermodynamic responses*[36]*, thereby overwhelming species that are already at the physiological limits of thermal and water stress*[14,27]*.*

Comment #34: L260: choose symbiosis or mutualism.

Response: Thanks. We have revised "mutualistic symbiosis" to "symbiosis.

Comment #35: L270–295: a crucial point that is missing is that while you quantify drought exposure, we urgently need science on drought vulnerability, to know which species are most likely to decline under this increased drought exposure. Species-specific drought vulnerability is the missing link between estimating their future exposure and knowing how this will affect them, which can facilitate conservation prioritization. Currently, drought vulnerability of most vertebrates is not well known (van den Bosch et al., 2025).

Response: Thank you for your important insight. This insight fundamentally reshapes our analysis. We use the historical drought severity extremes (99th percentile) experienced by a species within its realized niche as a proxy for drought tolerance limits. By shifting from a generic drought threshold to a species-specific historical extreme, we effectively integrated drought vulnerability into our exposure assessment. We believe this directly addresses the "missing link" you identified. The quantitative details for species-specific historical drought severity extremes are as follows:

L461–484:
Defining species-specific historical drought severity extremes
The intensification of drought severity will force organisms to endure unprecedented environmental stresses[13,14,17]*, but the exact impacts are contingent upon species-specific drought vulnerability*[18,40]*. However, physiological data on drought vulnerability for most species remain unavailable*[40]*. In fact, a species' geographic distribution results from long-term adaptation to climatic conditions*[56]*, and the climatic extremes experienced across its realized distribution likely represent its climatic niche limits*[38,56,89,90]*. Therefore, we applied methods similar to studies on temperature extremes*[36,38]*, approximating species-specific drought tolerance limits by estimating historical drought severity extremes within species' realized niches. Specifically, species-specific historical drought severity extremes were calculated as the 99th percentile of drought severity experienced across all grid cells within each species' expert-validated global distribution during the historical baseline (Supplementary Figs. 1 and 20). This percentile-based method reduces the effects of extreme outliers and overestimated species ranges*[36,81]*. Importantly, considering recent droughts have already imposed significant impacts on ecosystems*[14-16,21,22]*, we use the pre-industrial period (1851 to 1900, characterized by*

relatively stable climate conditions) as the historical baseline. It must be underlined that species drought tolerance limits estimated through realized niches may be conservative, as species may not have reached their actual physiological limits in the pre-industrial period. Furthermore, some species' range maps may underestimate the extent of their climatic niche due to limited long-term historical records for calibration[80]. Phenotypic plasticity of species to drought stress and species interactions may lead to misjudgments of drought tolerance limits by applying historical drought severity extremes[13,57].

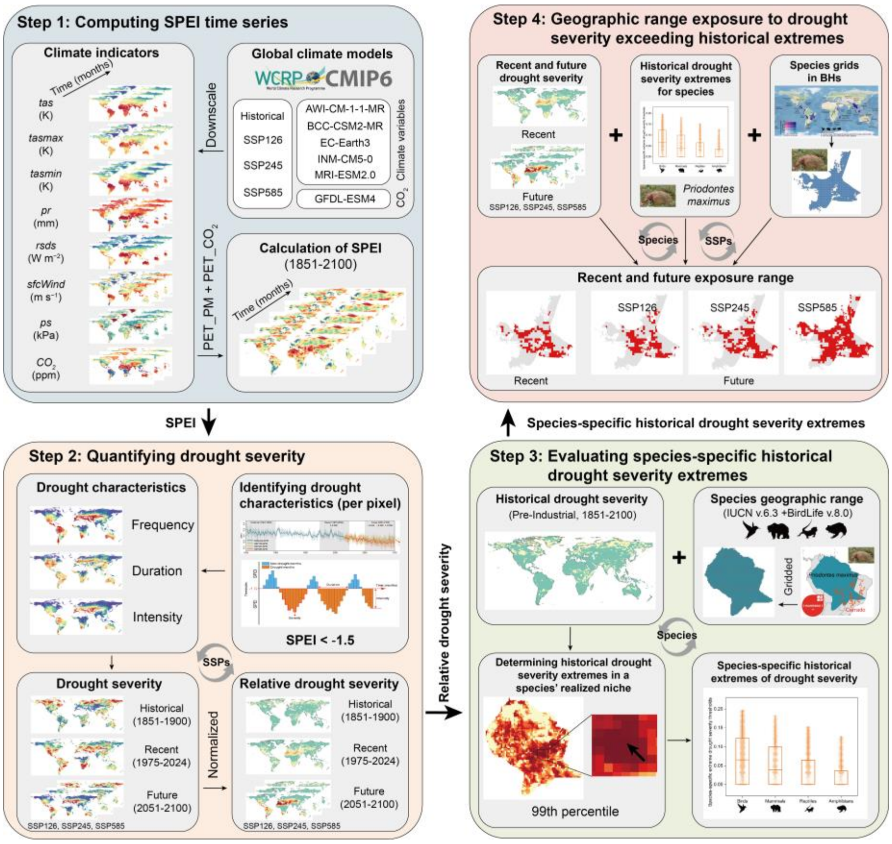

Supplementary Fig. 1 Overview of methods used for assessing species geographic range exposure to drought severity beyond realized niche limits. *Illustration using the geographic range of Priodontes maximus as a vulnerable species found in the Cerrado (image of Priodontes maximus from IUCN).* ***Step 1:*** *calculate monthly time series of the standardized precipitation-evapotranspiration index (SPEI) using GCMs data from CMIP6, where tas represents the monthly mean near-surface air temperature, tasmax represents the monthly maximum near-surface air temperature, tasmin represents the monthly minimum near-surface air temperature, pr represents the monthly total precipitation, rsds represents the monthly total surface shortwave radiation, sfcWind represents the monthly mean near-surface wind speed, ps represents the monthly mean surface air pressure, and CO2 represents atmospheric carbon dioxide concentration;* ***Step 2:*** *quantify drought severity with a threshold of SPEI < –1.5;* ***Step 3:*** *evaluate species-specific historical drought severity extremes based on historical realized niche limits;* ***Step 4:*** *assess species geographic range exposure to drought severity exceeding historical extremes.*

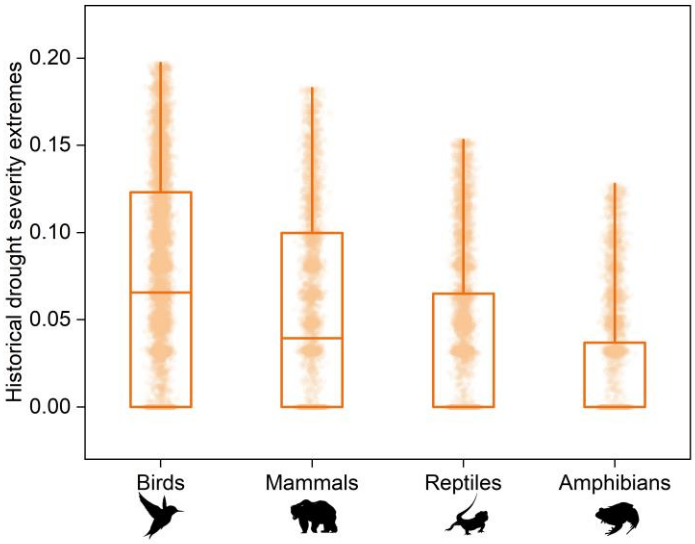

Supplementary Fig. 20 Species-specific historical drought severity extremes for terrestrial vertebrates. *The box plot shows the median and the 25th and 75th percentiles, with whiskers representing the 5th and 95th percentiles.*

Comment #36: L301: that is under the assumption profitable agriculture and forestry will actually benefit the local population…

Response: Thank you for the insightful comment. We aimed to illustrate that conservation efforts may be constrained by substantial development and livelihood needs in BHs.

To this end, we have tightened our narrative and removed references to opportunity costs, in order to avoid distracting from the main topic. As follows:

L248–253: *Emphatically, BHs facing greater drought exposure are also bearing substantial social burdens, implying that conservation efforts may be constrained by weak local governance capacity and substantial livelihood and development needs*[28,48]*, such as planning ecological corridors on land also valuable for agriculture or infrastructure*[29]*.*

Comment #37: L297–322: I appreciate the integration of social issues, but I think we're bringing in too many new terms here that are not fully explained and, to an extent, are only indirectly linked to your results (e.g. 'opportunity costs', 'IPLC-based conservation', 'differentiated responsibilities', etc.

Response: Thanks. In the revised manuscript, we have somewhat downplayed the discussion on social issues and avoided introducing too many new terms.

Our revised narrative focuses more on stressing that substantial social burdens in BHs may hinder conservation efforts for adaptation to drought exposure, while outlining potential

approaches to improve governance. The specific revisions are as follows:

L249–261: *Emphatically, BHs facing greater drought exposure are also bearing substantial social burdens, implying that conservation efforts may be constrained by weak local governance capacity and substantial livelihood and development needs[28,48], such as planning ecological corridors on land also valuable for agriculture or infrastructure[29]. Therefore, expanding protection networks in BHs to adapt to intensifying drought exposure entails integrating social well-being. To this end, promoting land-use intensification or de-intensification in suitable areas could simultaneously restore natural habitats while matching land-use demands for socioeconomic development[62,63]. Moreover, strengthening cost-sharing and fiscal transfer mechanisms for conservation[64], facilitating transboundary collaboration[65], and recognizing conservation approaches rooted in indigenous and local communities[66] are essential for reducing conflicts and improving governance, particularly in BHs that share habitats or water systems.*

Comment #38: L331: this goes both ways, some species may have good buffers, others may be more vulnerable than we currently know. I'd generalize this to say the missing link is learning more about species-specific drought vulnerability.

Response: Thanks for the comment. Following your valuable comments, our refined analysis incorporated species-specific drought vulnerability, which may provide a more accurate profile of the hazardous drought conditions that threaten species persistence (see response to comment #35). Accordingly, we have revised our discussion of the assessment's limitations:

Revised manuscript

L264–270: *However, geographic range exposure to drought severity beyond realized niche limits may not necessarily imply local biodiversity loss. The fundamental niche limits of species may be wider than their realized niche limits[38,56]. Some species may buffer drought exposure through behavioral adaptations or phenotypic plasticity, such as utilizing microhabitats[50], shifting seasonal activities[67], adapting new habitats to alter current range[56], or evolving physiological tolerance to drought[13].*

Comment #39: L324: perhaps to include in this 'limitations' paragraph is the fact that you had to use an initial drought threshold for your calculations (currently SPEI-12 of -0.5). If many past months had -0.49 and many of the projections have -0.51, that will greatly increase drought frequency (part of your severity calculations) despite there being little actual change. This is difficult to avoid, even if you change the threshold (which I think is necessary).

Response: Thanks. We have added relevant discussion on this threshold effect in the *Discussion* (L270–271) and the *Methods* (L449–451). As follows:

L270–271: *Furthermore, exposure estimates may be influenced by the drought-threshold effect, which determines the drought severity (see Methods).*

L449–451: *It must be stressed that when SPEI values within a time series cluster near drought threshold, such methods may amplify drought severity by overestimating drought frequency.*

Comment #40: L360–364: this is another part that needs extensive rewriting, it is extremely difficult to follow.

Response: We have rewritten the sentence:

L294–297: *Prioritizing and safeguarding drought-vulnerable BHs and species holds the potential to prevent intensifying drought from undermining the achievement of global biodiversity targets, such as the GBF.*

Methods:

Comment #41: L482–493: I'm not sure this level of detail is needed here, just a reference may be sufficient.

Response: Thanks. Incorporating Reviewer #1's comments, we have rerun our PET calculation to include the effects of CO_2. Consequently, we have retained the PET_PM calculation and added details for the PET_CO_2 calculation. Of course, we have tightened our writing:

L412–427: *PET formulation usually employs the Penman-Monteith equation (PET_PM)[84] and one of its variants that accounts for decreased evapotranspiration due to increased surface resistance driven by rising atmospheric CO_2 concentrations (PET_CO_2)[85], which are respectively parameterized as:*

$$PET\_PM = \frac{0.408\Delta(R_n - G) + \gamma \frac{900}{T_{avg} + 273} u_2(e_s - e_a)}{\Delta + \gamma(1 + 0.34u_2)} \quad (1)$$

$$PET\_CO_2 = \frac{0.408\Delta(R_n - G) + \gamma \frac{900}{T_{avg} + 273} u_2(e_s - e_a)}{\Delta + \gamma(1 + (0.34 + 2.4 \times 10^{-4}([CO_2] - 300))u_2)} \quad (2)$$

where Δ is the gradient of the saturated vapor pressure curve, R_n is the net radiation at the crop surface, G is the soil heat flux density, γ is the psychrometric constant, T_{avg} is the near-surface temperature, u_2 is the near-surface wind speed, $e_s - e_a$ is the saturated and actual vapor pressure deficit, these parameters can be derived from the climate variables collected from GCMs of CMIP6. Atmospheric CO_2 concentration ($[CO_2]$) grid data were obtained from the GFDL-ESM4 model in CMIP6, which shows strong correlation with observations from the Global Monitoring Laboratory (Pearson's $r > 0.99$, $p < 0.001$, Supplementary Fig. 18). Notably, the PET_CO_2 is modeled based on non-water-limited surface[85]. Therefore, we estimated PET using the average of the PET_PM and PET_CO_2 models for global drylands[19] (as defined by UNEP-WCM), while employing the PET_CO_2 model for other regions.

Comment #42: L498–509: same as above, I think the concept of SPEI can be explained in simpler terms.

Response: We have further tightened the wording about SPEI:

L427–429: *The SPEI is further calculated by transforming the empirical probability of the difference between monthly precipitation and PET into the standard normal distribution function*[83].

Comment #43: L524: I have an issue with the drought threshold used here. The lower threshold could be described as mildly dry, or even near-normal. This is problematic, because due to the calculation of drought severity, a large increase in the number of months with slightly drier conditions that are still not considered moderate, severe, or extreme drought (per the SPEI index), could lead to the quantification of 'extreme drought' (while in SPEI-terms, 'extreme drought' would be limited to numbers below SPEI-12 = -2).

Firstly, I think it is necessary to rerun the analysis with a more common drought threshold (e.g. SPEI-12 = -1.5), because you are inferring 'extreme drought' for high percentages of species' ranges, while in many cases it may in fact be long-term gradual shifts from 'dryish' to 'quite dry'. With a stronger threshold there will be fewer events to consider but they will be more likely to have biological impacts, and so the change in drought you calculate will be more likely to have the grave impacts you postulate.

Secondly, I would then rephrase 'extreme drought' to 'extreme changes in drought severity', to avoid confusion, given your findings of changing drought patterns may or may not pertain to severe/extreme droughts in SPEI-12 terms.

Response: Thanks. Following your valuable suggestion, we adopted the stricter threshold of SPEI < –1.5 (Severe Drought). This ensures that we are not categorizing 'mildly dry' years as drought events, but focusing only on biologically significant drought conditions. This makes our results more conservative and robust.

Furthermore, based on our refined analysis, we have revised the term "extreme drought" to "drought severity exceeding historical extremes" to avoid confusion.

The specific revisions are as follows:

L440–444: *We categorized a drought event as an occurrence with the SPEI value at least two consecutive months below the drought threshold (SPEI < –1.5), which corresponds to the lower threshold for severe drought as defined by the commonly used U.S. Drought Monitor classification scheme*[9,86].

Comment #44: L627: calculating

Response: Thanks. We have rewritten the sentence as follows:

L545–547: *The Python codes associated with this study are available at https://doi.org/10.11888/Terre.tpdc.302324. More information about the codes and data can be obtained from the corresponding author on request.*

Figures:

Comment #45: Fig 1: I'm not sure how helpful the tan outlines are, they confused me until I realized they're likely meant to delineate the hotspots?

Response: We have added a legend and caption for the orange lines in Fig. 1. As follows:

L761–771:

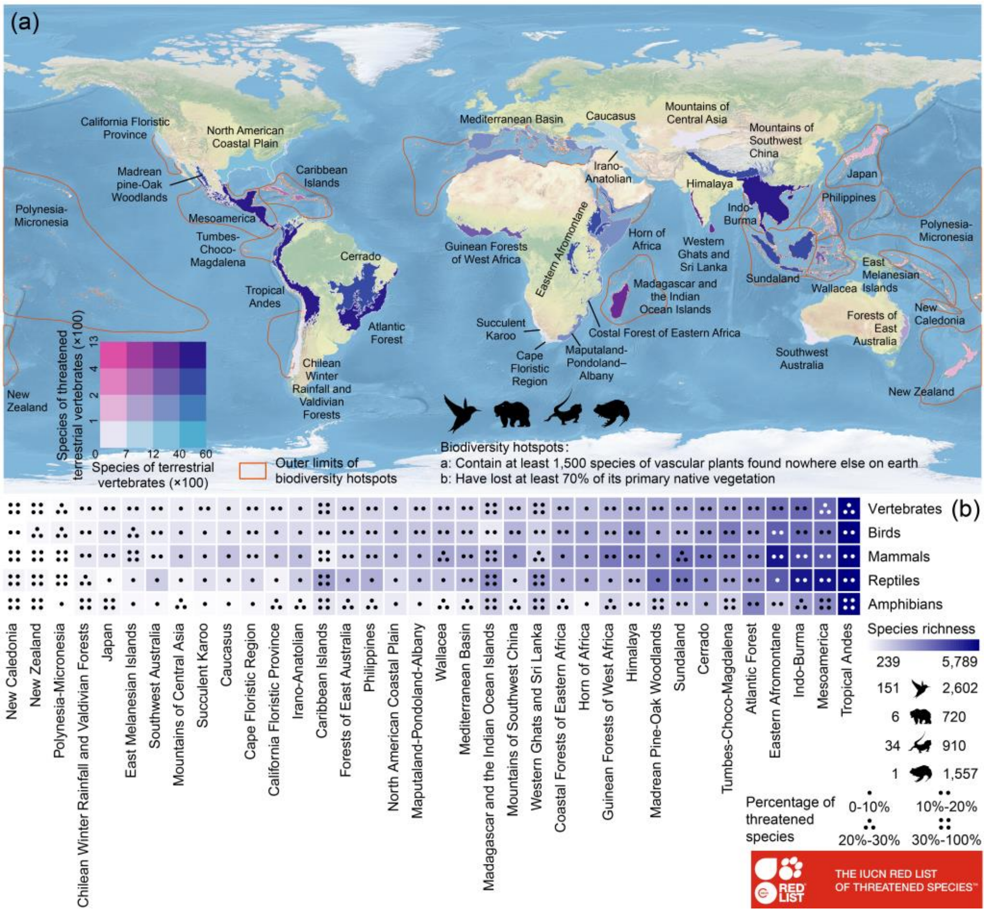

*Fig. 1 Location and terrestrial vertebrates of BHs. **a**, Map of 36 BHs, with the bivariate map showing the species richness of all and threatened terrestrial vertebrates, with the orange lines representing the outer limits of BHs. The background image is from Natural Earth (https://www.naturalearthdata.com/). **b**, Species richness of all terrestrial vertebrates, birds, mammals, reptiles, and amphibians in each BH, with dots representing the percentage of threatened species in that category. One, two, three, and four dots denote 0-10%, 10-20%, 20-30%, and 30-100%, respectively. Terrestrial vertebrate species richness*

data are obtained from the International Union for Conservation of Nature (IUCN) and the BirdLife International. Threatened species are those classified as vulnerable, endangered, or critically endangered by the IUCN Red List of Threatened Species.

Comment #46: Fig 3: again a very informative figure, though I have a hard time discerning which line refers to which SSP. Perhaps it would be possible to use three more contrasting colors, but then use two different patterns (solid / striped, for example). It may also confuse some readers that a striped vertical line is used for different purposes in 3a-e versus 3f.

Response: Thank you for the helpful suggestion. Our updated analysis focuses on drought exposure for the recent and future three scenarios. We have redesigned the graphs for clarity. Each color in the figure represents the same period: dark blue for recent, green for SSP126, orange for SSP245, and red for SSP585. As follows:

L786–798:

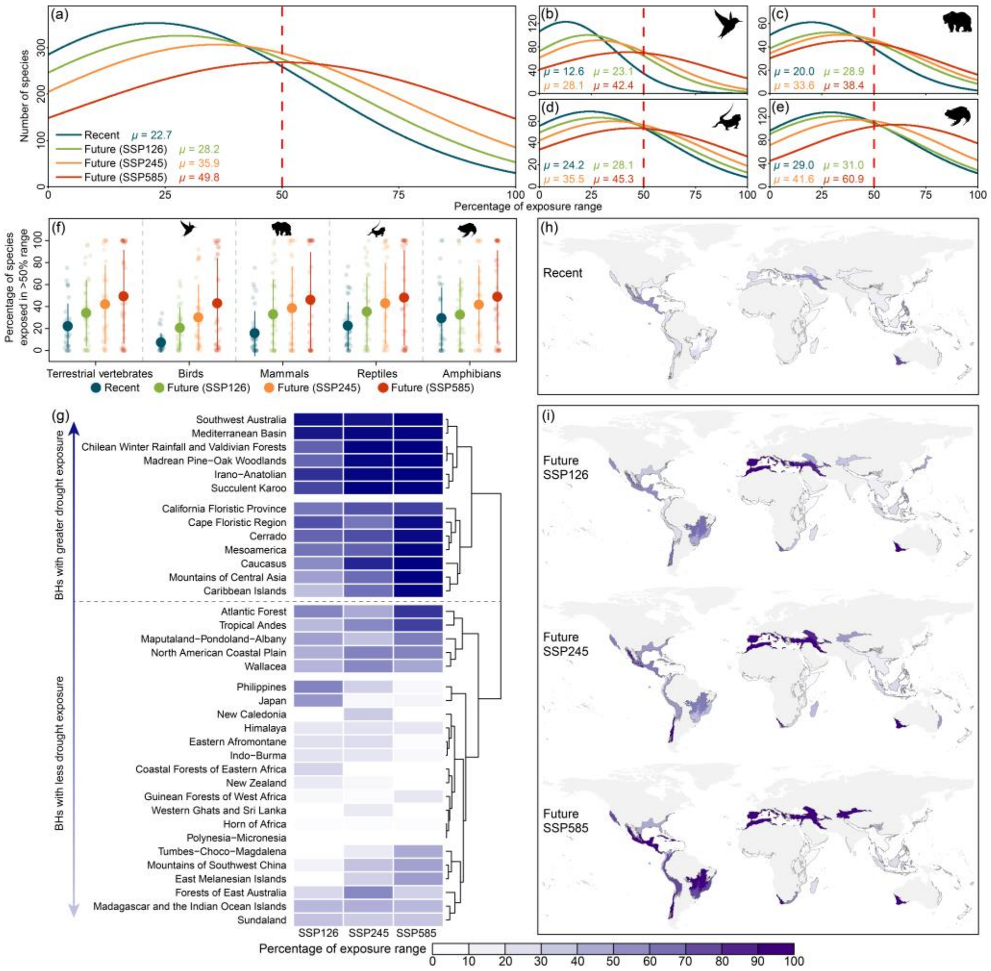

Fig. 3 Species geographic range exposure to drought severity exceeding historical extremes in BHs. a-e, *Distribution of percentage of exposure range to drought severity exceeding historical extremes for all threatened terrestrial vertebrates (a), birds (b), mammals (c), reptiles (d), and amphibians (e) in BHs*

under recent and three future scenarios, with μ denoting the overall mean of the distribution. ***f,*** *The percentage of species exposed to drought severity exceeding historical extremes in ≥ 50% of their geographic range for threatened terrestrial vertebrates, birds, mammals, reptiles, and amphibians in BHs under recent and three future scenarios, with dots representing each BH and whiskers representing standard deviation.* ***g,*** *Average percentage of exposure range to drought severity exceeding historical extremes for threatened terrestrial vertebrates in each BH under three future scenarios, clustering all SSPs into greater and less drought exposure.* ***h*** *and* ***i****, Spatial patterns of average percentage of exposure range to drought severity exceeding historical extremes for threatened terrestrial vertebrates in BHs under recent (****h****) and three future scenarios (****i****).*

Comment #47: Fig. 4: small mistake in the annotations, both mention BHs with lower species drought crisis.

Response: Thanks. We have corrected this error and updated the figure. As follows:

L799–808:

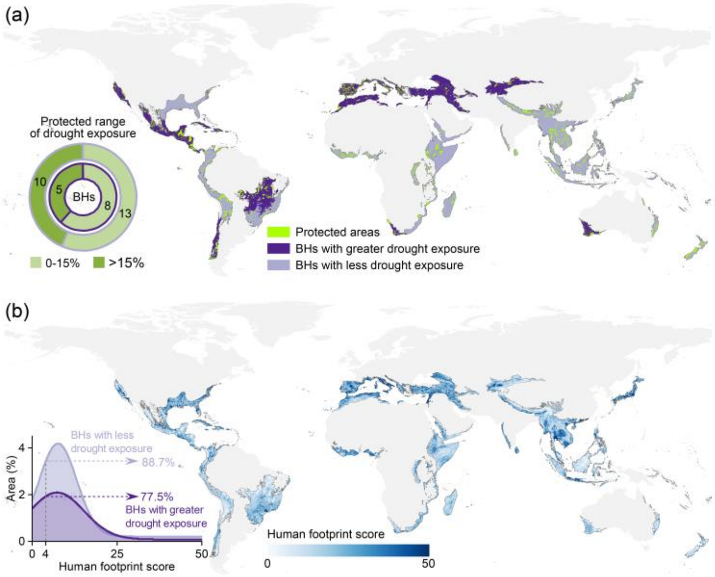

Fig. 4 Protected areas and human pressure in BHs. a, *Distribution of protected areas in BHs. The left panel shows the current protection status of regions that will face future drought exposure: light green, 0-15% range protected; green, > 15% range protected. The percentage of protected area coverage is the average across the three SSPs. The inner circle shows the number of BHs with greater drought exposure, and the outer circle shows the number of BHs with less drought exposure.* ***b,*** *Distribution of human footprints in BHs. The left graph shows the frequency distribution of human footprints in BHs with different future drought exposure levels: light purple, BHs with less drought exposure; purple, BHs with greater drought exposure. It also shows the percentage of area under intense human pressure (i.e., human footprint score > 4).*

Comment #48: Fig. 5: I'm unsure about this figure: while visually appealing and conceptually interesting, this figure appears to extend substantially into social science and conservation policy, which is not directly part of the analysist. A lot of themes are introduced that, while important, feel somewhat disconnected from the empirical results presented earlier. There is enough material here to warrant separate studies, and its inclusion risks diluting the paper's focus on drought and biodiversity. I should note that I am less familiar with these topics, and will therefore leave it to the editor and other reviewers to judge whether the inclusion of this figure strengthens the manuscript or detracts from its focus.

Response: Thank you for your important comment. In our revised manuscript, we have somewhat downplayed the discussion on social issues and conservation policies. Consequently, we have simplified this figure.

L809–816:

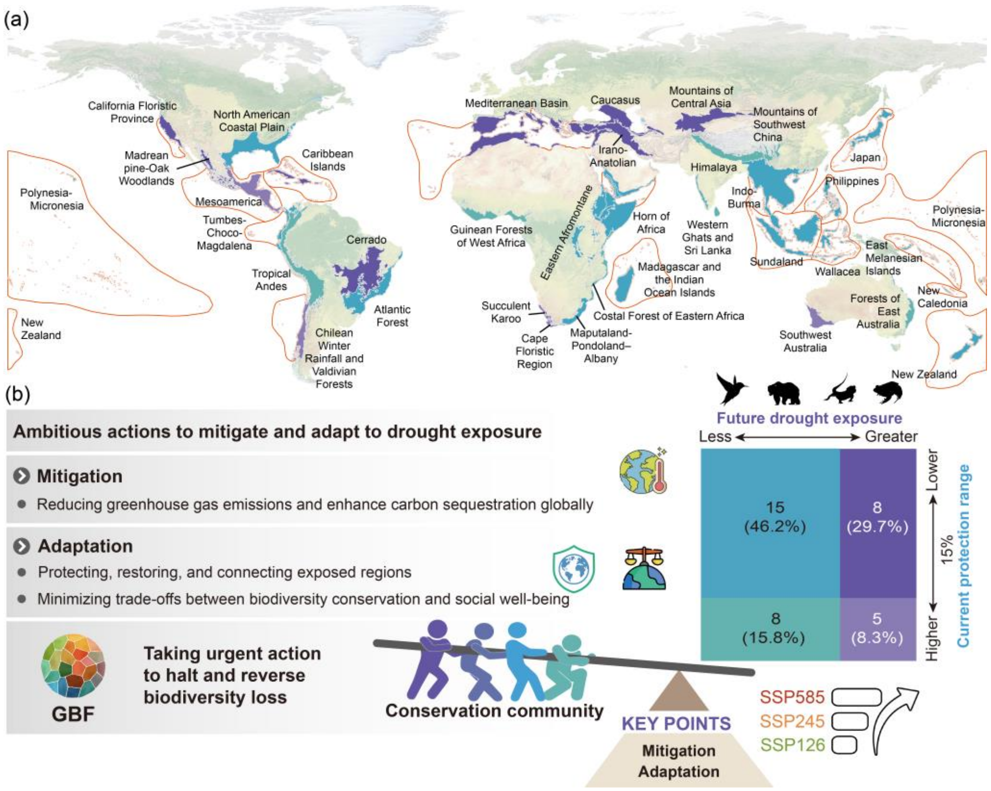

Fig. 5 Mitigation and adaptation framework for drought exposure in BHs. *The binary scheme shows the intersection between future drought exposure and current protection status in BHs (**a**), with corresponding figures representing the number and percentage of area for classified BHs (**b**). To mitigate and adapt to unprecedented drought exposure in BHs, the international community should immediately reduce greenhouse gas emissions and enhance carbon sequestration to mitigate climate change, and conservation efforts should prioritize protecting, restoring, and connecting exposed areas while balancing social well-being.*

Reviewer #2 (Remarks on code availability):

Comment #49: I do not foresee to have the time necessary to review the code.

Response: Thanks. Our refined analysis has updated the Python codes, as detailed at https://doi.org/10.11888/Terre.tpdc.302324.

Dear reviewers,

Thank you sincerely for your valuable comments on our work. We have carefully and rigorously refined our analysis, responded to your comments point by point, and revised the manuscript accordingly. A summary of our main revisions is as follows:

(1) Improved clarity: Considering all reviewers' concerns, we have improved the overall writing by revising phrasing and restructuring paragraphs to enhance flow and readability.

(2) Refined PET modeling: As suggested by Reviewer #1, we have clarified the rationale for employing the average of PET_PM and PET_CO_2 models to estimate PET in drylands to avoid over- or under-estimation.

(3) Additional analyses of vertebrate hotspots. Considering Reviewer #2's concerns, we have performed additional analyses on drought exposure in terrestrial vertebrate diversity hotspots to address potential biases in the definition of Biodiversity Hotspots.

(4) Re-analysis of resident species: As suggested by Reviewer #2, to ensure our exposure estimates are biologically robust, we have re-run all analyses based on year-round resident terrestrial vertebrates.

We hope these revisions have satisfactorily addressed the issues raised. The revised Manuscript, Supplementary Information, and Source Data for all updated Figures have been submitted simultaneously. Please do not hesitate to contact us if further material or information is needed.

Yours sincerely,

Jian Sun

On behalf of all co-authors

Response to Reviewer #1

Reviewer #1 (Remarks to the Author):

Thanks for the very deep and clear revisions.

I have only made very few minor comments and fixed typos etc. in the attached ms.

all the best

christopher reyer

Response: Dear Dr. Reyer, we appreciate you for your constructive comments, which have greatly improved our manuscript. We have addressed all suggested revisions below.

Abstract:

Comment #1: Replace “for” to “of”

Response: Thank you. We have made this correction:

L38–40: *“Importantly, a low-warming future (SSP126) will reduce the average exposure range by 7.6% compared to SSP245, highlighting the urgency of ambitious climate mitigation.”*

Results:

Comment #2: BY design, none of the ssp-rcps is more realistic than any other. so it is more a "middle-of-the-road" scenario or "intermediate" as you write. but I would omit the "realistic".

Response: Thank you for highlighting this important nuance. We have described SSP245 as “an intermediate-emission scenario” throughout the text and removed references to it being “realistic”.

L96–100: *“To cover the potential range of future anthropogenic climate change, we estimated projections under three warming scenarios: Shared Socioeconomic Pathway 1-2.6 (SSP126), a low greenhouse gas emission scenario; SSP245, an intermediate-emission scenario; and SSP585, a high-emission scenario (see Methods for details).”*

Comment #3: I think the "potentially" is misleading. rather sth like “bearing the risk of offsetting...”

Response: Thanks, we have revised the original sentence to:

L168–170: *“Moreover, the expansion of bioenergy cropland[41] and renewable energy[29] promoted by climate mitigation policies may accelerate habitat losses in BHs, thereby risking the offset of biodiversity gains from slowing warming.”*

Comment #4: Remove "use"

Response: Done.

Discussion:

Comment #5: Add the words "been" and "their".

Response: Thanks. We have modified the sentence accordingly:

L207–209: *"…we found that 22.5% of threatened terrestrial vertebrates in BHs have recently been exposed to drought severity exceeding their historical extremes across at least half of their current geographic range, …"*

Methods:

Comment #6: Revise "confirms" to "has identified".

Response: Done.

L308–309 *"Conservation International (https://www.conservation.org/) has identified 36 Biodiversity Hotspots (BHs) globally…"*

Comment #7: in which way are they actually funded? their protection? but not all the area of a BH is protected? this is unclear.

Response: We agree this was unclear. We have removed the mention of funding to focus on the biological definition of the hotspots.

L308–312: *"Conservation International (https://www.conservation.org/) has identified 36 Biodiversity Hotspots (BHs) globally (Fig. 1 and Supplementary Table 2), which are strictly defined as terrestrial regions that contain at least 1,500 species of vascular plants found nowhere else on Earth and have lost at least 70% of their primary native vegetation[1-3]."*

Comment #8: Replace "earth" to "Earth".

Response: Done.

Comment #9: Capitalize the initial letters of "representative concentration pathways (RCPs) and shared socioeconomic pathways (SSPs)".

Response: Revise.

Comment #10: Remove "realistic".

Response: Done.

L345–346: *"SSP245 represents an intermediate-emission scenario, with socioeconomic trends largely consistent with historical trajectories[78]."*

Comment #11: Does this average make sense? if it is a water limited surface (which I think is what you imply?)

Response: Thank you for this comment. We have clarified the justification for this approach in the *Methods*. As highlighted in previous studies (Lian et al., 2021; Milly et al., 2016), the conventional Penman–Monteith formulation (PET_PM) tends to overestimate PET changes under elevated CO_2 because it assumes fixed surface resistance of vegetation. Conversely, incorporating CO_2-sensitive surface resistance (PET_CO_2) reduces these estimates, but this model was developed on non-water-limited surfaces; applying it strictly to water-limited drylands may underestimate regional PET increases (Yang et al., 2019).

Because PET in drylands is expected to fall between estimates with and without the CO_2 effect (Lian et al., 2021), we employed the average of PET_PM and PET_CO_2 models for drylands to balance these biases, while using PET_CO_2 for other regions.

L430–436: *"Notably, the CO_2 effect on surface resistance in the PET_CO_2 was modeled on non-water-limited surfaces, and applying it to drylands may underestimate PET increases[86]. Previous research on dryland expansion under climate warming suggests that PET in drylands should fall between estimates with and without CO_2 effect[13]. Therefore, we estimated PET using the average of the PET_PM and PET_CO_2 models for global drylands (as defined by UNEP-WCM), while employing the PET_CO_2 model for other regions."*

Lian, X. et al. Multifaceted characteristics of dryland aridity changes in a warming world. Nat Rev Earth Env 2, 232-250 (2021).
Milly, P. C., & Dunne, K. A. Potential evapotranspiration and continental drying. Nature Climate Change 6, 946-949 (2016).
Yang, Y., Roderick, M. L., Zhang, S., McVicar, T. R. & Donohue, R. J. J. N. C. C. Hydrologic implications of vegetation response to elevated CO2 in climate projections. Nature Climate Change 9, 44-48 (2019).

Comment #12: I am not sure if this "infographic style" with all the illustrations is really needed or not rather distracting...

Response: Upon reflection, we agree. We have removed Figure 5 to avoid distracting from the core ecological analysis.

Response to Reviewer #2

Reviewer #2 (Remarks to the Author):

Please find my review report attached.

I would like to sincerely thank the authors for their hard work revising the manuscript, particularly rerunning analyses with a different threshold for extreme drought as well as extensively improving the clarity and readability of the text. While most comments below relate to minor issues regarding writing and phrasing, I have two major concerns that I believe need to be addressed before publication, and I apologize for not identifying these concerns in my first review. They relate to the treatment of breeding versus non-breeding ranges and the definition of biodiversity hotspots and are detailed below in my line-by-line comments.

Response: We sincerely thank you for your thoughtful and insightful comments. We have taken your major concerns very seriously. Specifically, we have added analyses regarding vertebrate hotspots to address potential biases introduced by the definition of hotspots, and we have re-run our analyses using year-round resident species to ensure accurate exposure estimates. The specific corrections are detailed below.

Abstract:

Comment #1: I'm still unsure about the use of the term 'adaptive' conservation, though other than that, the title and abstract are greatly improved. I note the abstract still mentions the twofold difference between SSP126 and 585 which is not reflected by L106–108.

Response: Thank you. We have removed the term "adaptive" (L29–30). Additionally, we revised the abstract to match the new results, emphasizing the quantitative reduction in exposure between SSP126 and SSP245 rather than the "twofold" phrasing (L38-40).

L29–30: *"Ambitious conservation efforts are needed to curb biodiversity loss as drought severity intensifies globally."*

L38–40: *"Importantly, a low-warming future (SSP126) will reduce exposure estimates of species by 8.5% compared to SSP245, highlighting the urgency of ambitious climate mitigation."*

Introduction:

Comment #2: L47: An important point that was not raised in the first round of review concerns the definition of biodiversity hotspots used in the study. The original Myers biodiversity hotspots were delineated based on patterns of endemic vascular plant diversity and therefore do not necessarily capture the best ratio of hotspot land area / vertebrate diversity (see Jenkins et al., 2013).

Ideally, the analyses would be adapted to global vertebrate diversity hotspots as identified by

Jenkins et al. (2013); for example, the 36 hotspots used here exclude the Amazon basin, despite it being the most vertebrate-diverse region globally. Given that this issue was not addressed in the initial review round, it may be more appropriate—at the editor's discretion—to acknowledge this mismatch as a limitation in the Discussion.

Response: Thank you for this crucial point. We agree that Biodiversity Hotspots (BHs) defined by vascular plants may overlook certain vertebrate-rich regions (e.g., the Amazon).

To address this, we performed an additional analysis of drought exposure using the terrestrial vertebrate diversity hotspots identified by Jenkins et al. (2013). By comparing the results, we found that: (1) Regardless of the hotspot definition used, our core conclusion remains unchanged: threatened terrestrial vertebrates face increasing drought exposure that exceeds their historical extremes, while limiting future warming can reduce this drought exposure; (2) The BHs we used capture the majority (71.9%) of terrestrial vertebrate diversity hotspots. Both assessments similarly reveal that mid-latitude drylands and tropical Americas are projected to face greater drought exposure.

Consequently, we have illustrated this limitation and the supporting results in the *Discussion* and *Supplementary Information*. As follows:

L281–287: "*Importantly, our analysis focused on BHs defined by the diversity of endemic vascular plants, potentially overlooking certain terrestrial vertebrate diversity hotspots (such as the Amazon basin)*[39,67]*. To this end, we performed an additional analysis based on terrestrial vertebrate diversity hotspots (Supplementary Fig. 16), which similarly reveals and confirms that threatened terrestrial vertebrates are increasingly exposed to drought severity exceeding their historical extremes, while limiting future warming will reduce this drought exposure.*"

Comment #3: L52–55: This sentence runs on a bit too long, perhaps omit the last part; 'and triggering…'.

Response: Revised as suggested.

L52–55: *"Recurring droughts degrade habitat quality, directly affecting species survival strategies, increasing physiological stresses such as heat stress and dehydration, causing reproduction failure and population decrease*[14-19]*."*

Comment #4: L69: 'weakening their dispersal' sounds odd.

Response: We have replaced it with "hindering their dispersal". As follows:

L67–69: *"…undermining potential climate refuges for species and hindering their dispersal to track their climate envelopes*[23,31]*."*

Comment #5: L81: The wording "to address the scarcity of physiological data" may be misleading, as it suggests that this study contributes new physiological data. A formulation such as "given the scarcity of physiological data…" would be more accurate. While the proposed drought exposure quantification represents a methodological advance and may improve our understanding of drought tolerance limits, it is beyond the scope of this research to validate such estimates. This limitation should be acknowledged in the Discussion, along with a clear statement that direct, species-specific physiological measurements remain essential for accurately characterizing drought tolerance.

Response: We accept this correction. We have rephrased the text to avoid implying we generated new physiological data (L80–82). In the *Discussion*, we explicitly acknowledge that our assessments are based on realized climatic limits, not direct physiological measurements (L265–266). We also emphasized the necessity of directly measuring and verifying species-specific physiological drought tolerance (L288–290).

L80–82: *"Given the scarcity of physiological data for most species[39], we approximated drought tolerance limits using the historical extremes of species realized climatic niches."*

L265–266: *"Our assessments of species drought tolerance are based on their realized climatic niche limits rather than direct physiological measurements."*

L288–290: *"Future research priorities are recommended to refine the investigation and validation of species-specific physiological drought tolerance and adaptation mechanisms across different climate scenarios, …"*

Results:

Comment #6: L69: L110–112: could be rephrased or removed, species persistence could also be impacted if the current warming trajectory closely resembled another SSP.

Response: We have removed the sentence as suggested.

Comment #7: L142: I would rephrase to 'face greater exposure' due to the specific meaning of drought vulnerability.

Response: Rephrased as suggested:

L142–143: *"On average, threatened reptiles and amphibians face greater drought exposure than birds and mammals in most BHs, …"*

Comment #8: L146: 'BH's' is missing at the end of the sentence.

Response: Incorporated.

L146–147: *"Nonetheless, threatened mammals face greater exposure than other taxa in the North American Coastal Plain and Irano-Anatolian BHs."*

Comment #9: L153–154: more informative if you also mention the SSP245 percentage. Is it still twofold, or is this an artefact from the previous analysis?

Response: Thank you. We have updated the text with specific exposure estimates for the different future scenarios based on our new analysis.

L152–157: *"Generally, limiting mean global warming to below 1.8 ℃ above pre-industrial levels by the end of the 21st century (SSP126) can effectively reduce the percentage of species that face drought severity exceeding historical extremes in ≥ 50% of their geographic range respectively by 8.5% from 36.5% under SSP245 (2.7 ℃ warming) and 22.0% from 50.0% under SSP585 (4.4 ℃ warming) (Fig. 3f)."*

Comment #10: L155–156: This sentence is incomplete; some grammatical mistakes are the cause.

Response: Thanks. We have rewritten the sentence as follows:

L157–159: *"In terms of reduced average exposure ranges, limiting future warming provides greater benefits for threatened amphibians and reptiles than for threatened birds and mammals (Fig. 3b–e)."*

Comment #11: L177: rephrase to something like 'have less than 15% of their area under protection', or 'have protected-area coverage below 15%'.

Response: Rephrased as suggested.

L178–179: *"Most individual BHs projected to experience greater drought exposure have protected-area coverage below 15%, …"*

Comment #12: L185: numerous implies a multiple will follow, evidence is singular

Response: We have corrected this mistake:

L187–188: *"Furthermore, numerous studies demonstrate that anthropogenic habitat conversion in BHs will be further amplified in the future[24,25,38]."*

Comment #13: L187: disarticulation is not a term I have seen used in this field of research and may confuse readers, I would rephrase

Response: We have replaced it with "reducing connectivity".

L188–189: ***"**Habitat loss and fragmentation from expanding human activities are reducing connectivity across BHs, ..."*

Comment #14: L188: perhaps range shifts are a better example of an adaptive strategy to climate change that may be compromised by habitat loss, as opposed to migratory behaviour.

Response: Excellent suggestion. We have replaced the term "migratory behavior" with "range shifts".

L188–191: *"Habitat loss and fragmentation from expanding human activities are reducing connectivity across BHs, undermining the potential adaptive strategies of species to drought (such as range shifts)[23,24,31]."*

Discussion:

Comment #15: L201: please proofread once again for unnecessary adjectives. For example, 'quantitatively' does not really add anything here. For readability, you could opt to fully write out important abbreviations at the start of each article section (e.g. write out Biodiversity Hotspots (BHs) in the first line of the discussion).

Response: We have carefully checked the phrasing to remove unnecessary adjectives and have written out important abbreviations at the start of sections as suggested.

Comment #16: L202: 'niche limits' is too vague here. What definition of niche? What limits? Perhaps opt for something more concrete, like 'exceeding the historical extremes experienced by species' or even 'tolerable environmental conditions'.

Response: Thank you. We have rephrased the sentence as suggested. As follows:

L204–205: *"Drought severity exceeding historical extremes experienced by species is detrimental to their survival[14-19]."*

Comment #17: L205: Again some minor writing issues: "recently been exposed", "half of their geographic range", "under a realistic" (the 'more' is isolated in this sentence, more than what?).

Response: Revised as suggested.

Comment #18: L208: Many adjectives again, 'compound extreme thermal events'

Response: Modified. Thank you.

L210–213: *"Mid-latitude dryland BHs will face severe drought exposure, where coupled thermal extremes may amplify thermodynamic responses[35], overwhelming species that are already at the physiological limits of heat and water stress[15,25]."*

Comment #19: L216–220: this is all true, but L212 (this is potentially explained by…) appears to imply this is the reason you found greater drought exposure for some taxa, while apart from range size, none of these elements were directly included in the analysis. In fact, you found these exposure differences between birds/mammals and amphibians/reptiles and, compounding with that, amphibians/reptiles may also be more vulnerable due to their respective ecologies.

Response: Thank you for clarifying the distinction between exposure and vulnerability. We have revised the text to ensure we do not conflate the two. We now clarify that range size explains exposure, while biological differences (vulnerability) compound the impact.

L213–217: *"Analyses across taxa highlight that threatened amphibians and reptiles face greater drought exposure than birds and mammals in BHs, potentially due to the smaller geographic ranges occupied by most threatened amphibians and reptiles (Supplementary Fig. 15). The impacts of drought exposure are compounded by divergent drought vulnerability and adaptability across taxa[17,39]."*

Comment #20: L237: than recent exposure levels

Response: Revised as suggested.

Comment #21: L242: remove 'behind'

Response: Done.

Comment #22: L297: the ending is stronger without the addition of 'such as the GBF'

Response: Great suggestion, we have modified this statement accordingly.

L302–304: *"Prioritizing and safeguarding drought-vulnerable BHs and species holds the potential to prevent intensifying drought from undermining the achievement of global biodiversity targets."*

Methods:

Comment #23: L302: what do you mean the BHs are funded by CEPF? I would remove the funding part of the sentence.

Response: We have removed the funding part:

L308–309: *"Conservation International (https://www.conservation.org/) has identified 36 Biodiversity Hotspots (BHs) globally (Fig. 1 and Supplementary Table 2), …"*

Comment #24: L306: I also noticed this 2.5% figure on the website of conservation.org, but visually this did not make sense to me as the hotspots appeared to cover a much higher percentage of earth's land mass. I downloaded the shapefile from their website and the total area of BH seems to be 24 million square kilometers, so closer to 15-16% of terrestrial surface? See also Jenkins et al. 2013 for estimates of how they were able to capture a higher percentage of vertebrate diversity within a lower percentage of earth's terrestrial surface.

Response: You are correct. The 2.5% figure refers to the remaining intact habitat, not the total area of the BHs. We have corrected this to ~16% to reflect the total terrestrial surface covered by BHs.

L312–314: *"These most biologically rich yet threatened regions cover only about 16% of the Earth's terrestrial surface but support nearly half of the world's endemic plants and terrestrial vertebrates, …"*

Comment #25: L307-309: as in the previous version, the sudden tie-in of indigenous communities appears quite out of the blue, and it seems misplaced in the methodology section.

Response: Thanks. We have removed the reference to indigenous and local communities here.

Comment #26: L358: remove 'the' before Birdlife International

Response: Revised as suggested.

Comment #27: L364–365: I would like to clarify whether the range filters were applied additively (AND) rather than inclusively (OR). Specifically, was the extracted range limited to areas that are simultaneously Breeding AND Native, rather than Breeding OR Native? If an OR criterion was used, this could be problematic for birds, whose breeding and non-breeding ranges are often separated by thousands of kilometers. In that case, (averaged) SPEI-12 across this range misrepresent actual SPEI-12 values encountered by the species in any given location. In other words, averaging SPEI-12 values across the combined breeding and non-breeding range includes many SPEI-12 values from locations that the species does not occupy at that time of year.

Even if an AND filter was applied, there are two important caveats. First, it should be noted that this analysis effectively only represents breeding ranges. Second, because SPEI-12 is a 12-month cumulative index, the values may not fully reflect the actual climate exposure for

migratory species.

For these reasons, we restricted our similar analysis to year-round resident species, for which the approach more reliably captures annual climate exposure (https://onlinelibrary.wiley.com/doi/10.1111/gcb.70272).

Response: We appreciate this vital feedback regarding the filtering of geographic ranges. We acknowledge that our original "breeding OR native" filter did not account for the temporal mismatch in migratory species (e.g., calculating annual SPEI-12 for a location a bird only visits briefly). To ensure our results are robust, we have re-run all analyses using only the geographic ranges of year-round resident terrestrial vertebrates, following the methodology you referenced. This ensures the climate data accurately reflects the conditions experienced by the species. We have updated the *Method* (L368–371) and all relevant results/figures throughout the manuscript.

Methods (L368–371): *"We used only the ranges of year-round resident terrestrial vertebrates (primarily excluding migratory birds), and omitted vertebrates in both marine and terrestrial habitats (e.g., Laridae, Phocidae, and Cheloniidae), to maintain consistency in drought exposure assessments*[39]*."*

Revised results (Excerpt): "*The estimates indicate that an average of 22.6% of geographic range in BHs for threatened terrestrial vertebrates has been exposed to drought severity exceeding their historical extremes in recent decades (Fig. 3a). .... Overall, 22.5% of threatened terrestrial vertebrates in BHs have recently experienced drought severity exceeding their historical extremes across at least half (≥ 50%) of their current geographic range, ...*"

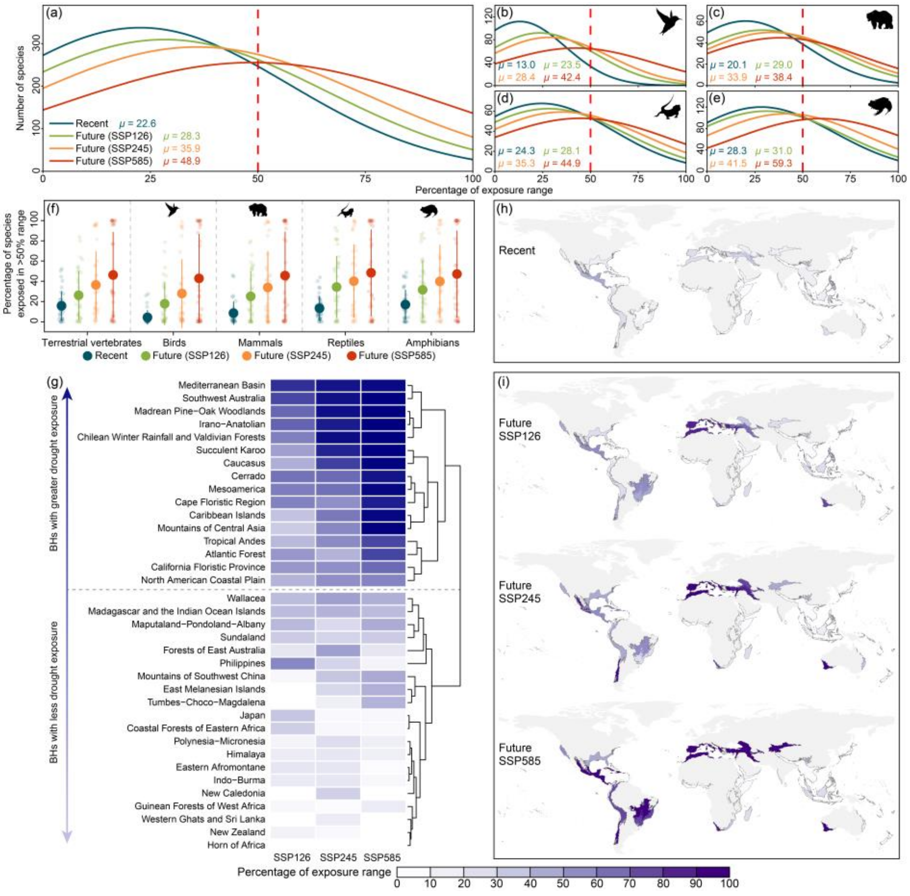

Fig. 3 Species geographic range exposure to drought severity exceeding historical extremes in BHs. ***a-e***, *Distribution of percentage of exposure range to drought severity exceeding historical extremes for threatened terrestrial vertebrates (**a**), birds (**b**), mammals (**c**), reptiles (**d**), and amphibians (**e**) in BHs under recent and three future scenarios, with μ denoting the overall mean of the distribution.* ***f***, *The percentage of species exposed to drought severity exceeding historical extremes in ≥ 50% of their geographic range for threatened terrestrial vertebrates, birds, mammals, reptiles, and amphibians in BHs under recent and three future scenarios, with dots representing each BH and whiskers representing standard deviation.* ***g***, *Average percentage of exposure range to drought severity exceeding historical extremes for threatened terrestrial vertebrates in each BH under three future scenarios, clustering all SSPs into greater and less drought exposure.* ***h*** *and* ***i***, *Spatial patterns of average percentage of exposure range to drought severity exceeding historical extremes for threatened terrestrial vertebrates in BHs under recent (**h**) and three future scenarios (**i**).*

References

Comment #28: Number 40 and 48 refer to the same article.

Response: Thanks. We have checked and corrected all references.

Figure 5:

Comment #29: I would remove the bottom row of graphics (GBF logo and slogan, conservation community, pyramid…). While these points are interesting to discuss, they are more appropriate for research that explicitly incorporates the human dimensions of conservation. In contrast, this paper focuses on ecological analyses and only touches briefly on societal aspects without contributing novel results in this regard.

Response: We agree. As noted in our response to Reviewer #1, we have removed Figure 5 entirely to maintain focus on the ecological analysis.

I would like to sincerely thank the authors for their hard work revising the manuscript, particularly rerunning analyses with a different threshold for extreme drought as well as extensively improving the clarity and readability of the text. While most comments below relate to minor issues regarding writing and phrasing, I have two major concerns that I believe need to be addressed before publication, and I apologize for not identifying these concerns in my first review. They relate to the treatment of breeding versus non-breeding ranges and the definition of biodiversity hotspots and are detailed below in my line-by-line comments.

Abstract

I'm still unsure about the use of the term 'adaptive' conservation, though other than that, the title and abstract are greatly improved. I note the abstract still mentions the twofold difference between SSP126 and 585 which is not reflected by L106–108.

Introduction

L47: An important point that was not raised in the first round of review concerns the definition of biodiversity hotspots used in the study. The original Myers biodiversity hotspots were delineated based on patterns of endemic vascular plant diversity and therefore do not necessarily capture the best ratio of hotspot land area / vertebrate diversity (see Jenkins et al., 2013).

Ideally, the analyses would be adapted to global vertebrate diversity hotspots as identified by Jenkins et al. (2013); for example, the 36 hotspots used here exclude the Amazon basin, despite it being the most vertebrate-diverse region globally. Given that this issue was not addressed in the initial review round, it may be more appropriate—at the editor's discretion—to acknowledge this mismatch as a limitation in the Discussion.

L52–55: This sentence runs on a bit too long, perhaps omit the last part; 'and triggering…'.

L69: 'weakening their dispersal' sounds odd.

L81: The wording "to address the scarcity of physiological data" may be misleading, as it suggests that this study contributes new physiological data. A formulation such as "given the scarcity of physiological data…" would be more accurate. While the proposed drought exposure quantification represents a methodological advance and may improve our understanding of drought tolerance limits, it is beyond the scope of this research to validate such estimates. This limitation should be acknowledged in the Discussion, along with a clear statement that direct, species-specific physiological measurements remain essential for accurately characterizing drought tolerance.

L110–112: could be rephrased or removed, species persistence could also be impacted if the current warming trajectory closely resembled another SSP.

L142: I would rephrase to 'face greater exposure' due to the specific meaning of drought vulnerability.

L146: 'BH's' is missing at the end of the sentence.

L153–154: more informative if you also mention the SSP245 percentage. Is it still twofold, or is this an artefact from the previous analysis?

L155–156: This sentence is incomplete; some grammatical mistakes are the cause.

L177: rephrase to something like ‘have less than 15% of their area under protection’, or ‘have protected-area coverage below 15%’.

L185: numerous implies a multiple will follow, evidence is singular

L187: disarticulation is not a term I have seen used in this field of research and may confuse readers, I would rephrase

L188: perhaps range shifts are a better example of an adaptive strategy to climate change that may be compromised by habitat loss, as opposed to migratory behaviour.

Discussion

L201: please proofread once again for unnecessary adjectives. For example, ‘quantitatively’ does not really add anything here. For readability, you could opt to fully write out important abbreviations at the start of each article section (e.g. write out Biodiversity Hotspots (BHs) in the first line of the discussion).

L202: ‘niche limits’ is too vague here. What definition of niche? What limits? Perhaps opt for something more concrete, like ‘exceeding the historical extremes experienced by species’ or even ‘tolerable environmental conditions’.

L205: Again some minor writing issues: “recently *been* exposed”, “half of *their* geographic range”, “under a realistic” (the ‘more’ is isolated in this sentence, more than what?).

L208: Many adjectives again, ‘compound extreme thermal events’

L216–220: this is all true, but L212 (this is potentially explained by…) appears to imply this is the reason you found greater drought exposure for some taxa, while apart from range size, none of these elements were directly included in the analysis. In fact, you found these exposure differences between birds/mammals and amphibians/reptiles and, compounding with that, amphibians/reptiles may also be more vulnerable due to their respective ecologies.

L237: than recent *exposure* levels

L242: remove ‘behind’

L297: the ending is stronger without the addition of ‘such as the GBF’

Methods

L302: what do you mean the BHs are funded by CEPF? I would remove the funding part of the sentence.

L306: I also noticed this 2.5% figure on the website of conservation.org, but visually this did not make sense to me as the hotspots appeared to cover a much higher percentage of earth’s land mass. I downloaded the shapefile from their website and the total area of BH seems to be 24 million square kilometers, so closer to 15-16% of terrestrial surface? See also Jenkins et al. 2013 for estimates of how they were able to capture a higher percentage of vertebrate diversity within a lower percentage of earth’s terrestrial surface.

L307-309: as in the previous version, the sudden tie-in of indigenous communities appears quite out of the blue, and it seems misplaced in the methodology section.

L358: remove ‘the’ before Birdlife International

L364–365: I would like to clarify whether the range filters were applied additively (AND) rather than inclusively (OR). Specifically, was the extracted range limited to areas that are simultaneously Breeding **AND** Native, rather than Breeding **OR** Native? If an OR criterion was used, this could be problematic for birds, whose breeding and non-breeding ranges are often separated by thousands of kilometers. In that case, (averaged) SPEI-12 across this range misrepresent actual SPEI-12 values encountered by the species in any given location. In other words, averaging SPEI-12 values across the combined breeding and non-breeding range includes many SPEI-12 values from locations that the species does not occupy at that time of year.

Even if an AND filter was applied, there are two important caveats. First, it should be noted that this analysis effectively only represents breeding ranges. Second, because SPEI-12 is a 12-month cumulative index, the values may not fully reflect the actual climate exposure for migratory species.

For these reasons, we restricted our similar analysis to year-round resident species, for which the approach more reliably captures annual climate exposure (https://onlinelibrary.wiley.com/doi/10.1111/gcb.70272).

References
Number 40 and 48 refer to the same article.

Figure 5:

I would remove the bottom row of graphics (GBF logo and slogan, conservation community, pyramid…). While these points are interesting to discuss, they are more appropriate for research that explicitly incorporates the human dimensions of conservation. In contrast, this paper focuses on ecological analyses and only touches briefly on societal aspects without contributing novel results in this regard.
